# Mass Spectrometry-Based Techniques to Elucidate the Sugar Code

**DOI:** 10.1021/acs.chemrev.1c00380

**Published:** 2021-09-07

**Authors:** Márkó Grabarics, Maike Lettow, Carla Kirschbaum, Kim Greis, Christian Manz, Kevin Pagel

**Affiliations:** † Institute of Chemistry and Biochemistry, Freie Universität Berlin, Arnimallee 22, 14195 Berlin, Germany; ‡ Department of Molecular Physics, Fritz Haber Institute of the Max Planck Society, Faradayweg 4−6, 14195 Berlin, Germany

## Abstract

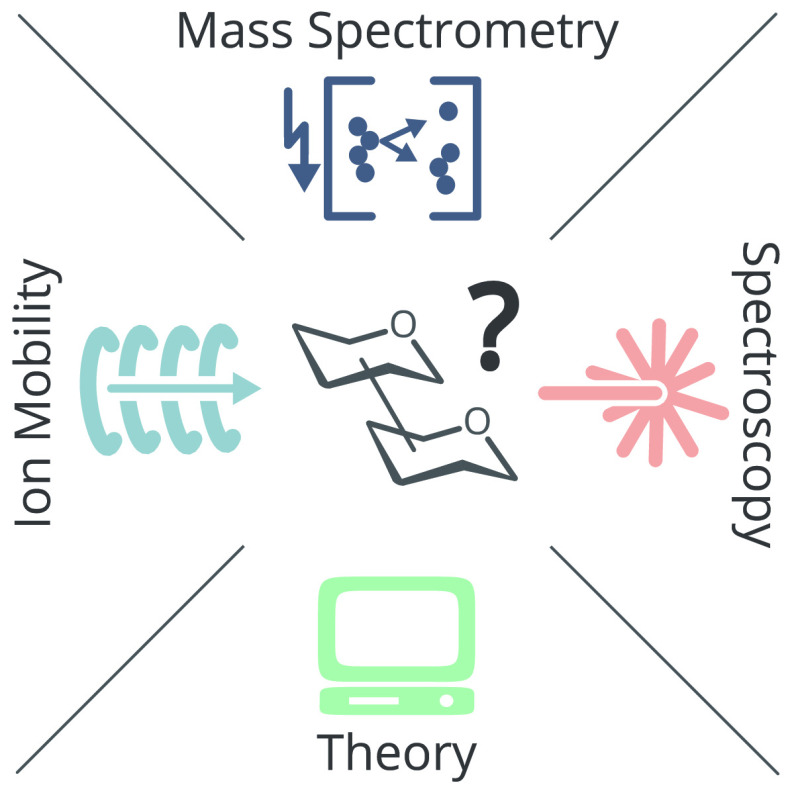

Cells encode information in the sequence of biopolymers, such as nucleic acids, proteins, and glycans. Although glycans are essential to all living organisms, surprisingly little is known about the “sugar code” and the biological roles of these molecules. The reason glycobiology lags behind its counterparts dealing with nucleic acids and proteins lies in the complexity of carbohydrate structures, which renders their analysis extremely challenging. Building blocks that may differ only in the configuration of a single stereocenter, combined with the vast possibilities to connect monosaccharide units, lead to an immense variety of isomers, which poses a formidable challenge to conventional mass spectrometry. In recent years, however, a combination of innovative ion activation methods, commercialization of ion mobility–mass spectrometry, progress in gas-phase ion spectroscopy, and advances in computational chemistry have led to a revolution in mass spectrometry-based glycan analysis. The present review focuses on the above techniques that expanded the traditional glycomics toolkit and provided spectacular insight into the structure of these fascinating biomolecules. To emphasize the specific challenges associated with them, major classes of mammalian glycans are discussed in separate sections. By doing so, we aim to put the spotlight on the most important element of glycobiology: the glycans themselves.

## 1 Introduction

Carbohydrates—often referred to as glycans—are the most abundant organic polymers found on Earth. They are essential to all known living organisms and regulate a variety of vital functions.^1^ Structurally, glycans are composed of monosaccharide building blocks linked together via glycosidic bonds. The certainly most prominent form are polysaccharides: mostly linear polymers with a regular structure and a high degree of polymerization. They are highly abundant and omnipresent in our daily life, for example, in the form of starch during cooking or as cellulose in plant tissues.^2^ In biology, however, smaller but structurally more diverse structures are often found to play an important role. Glycosylation is the most abundant and most complex post-translational modification (PTM) found in proteins.^3,4^ Here, highly branched glycan structures consisting of 10 to 20 monosaccharide building blocks are covalently attached to certain residues in the protein chain. The involved glycans are usually exposed to the exterior of the protein, where they promote protein folding^5,6^ and regulate a variety of often remarkably specific functions, such as immune response^7,8^ and fertilization.^9−11^ Similarly complex structures can also occur as free oligosaccharides, for example in milk, where they serve as crucial constituents to develop and retain a healthy microbiota.^12^ Also the extracellular matrix (ECM) is crowded with sugars, in particular glycosaminoglycans (GAGs), that are attached to proteins as proteoglycans.^13,14^ Even though GAGs are usually linear assemblies of up to 250 repeating disaccharide units, they are diversely sulfated at different sites in the molecule, making them exceptionally complex and heterogeneous.

**Figure 1:**
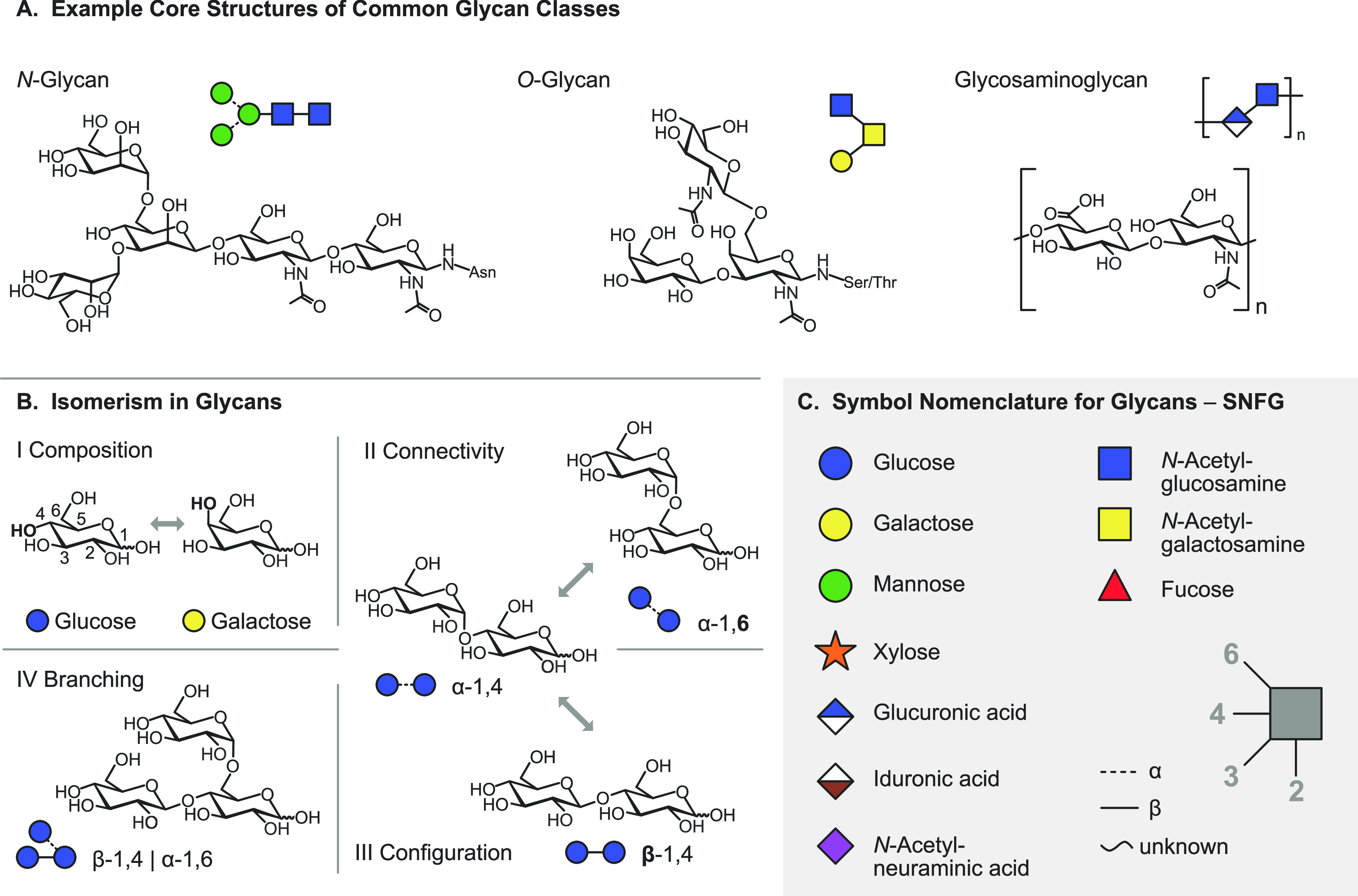
Representation of glycan structures and frequently occurring isomers. (A) Example core structures of *N*-glycans, *O*-glycans, and glycosaminoglycans are represented using chemical structures and equivalent symbol nomenclature. (B) Isomerism in glycans occurs on different levels: glycan composition, connectivity, configuration, and branching. (C) The symbol nomenclature for glycans offers simplified but unambiguous representations of complex glycan structures. Symbols are shown for the most abundant monosaccharides found in vertebrates.

Example structures of abundant glycan classes are shown in Figure 1A. Unlike the biosynthesis of DNA (replication), RNA (transcription), or proteins (translation), glycan biosynthesis is not a template-driven process. Instead, glycans are assembled in a complex cascade of *en bloc* attachment and multiple consecutive de- and reglycosylation steps.^15^ The structural diversity in the resulting oligosaccharides is therefore vast and determined by multiple parameters (Figure 1B). The monosaccharide composition (I) describes the types of building blocks that are connected. Monosaccharides can be depicted either by chemical structures or by employing the simplified symbol nomenclature for glycans (SNFG, Figure 1C). In vertebrates, the number of monosaccharides is limited to d-glucose (Glu), d-galactose (Gal), d-mannose (Man), *N*-acetyl-d-glucosamine (GlcNAc), *N*-acetyl-d-galactosamine (GalNAc), d-glucuronic acid (GlcA), l-iduronic acid (IdoA), d-xylose (Xyl), l-fucose (Fuc), and *N*-acetyl-d-neuraminic acid (Neu5Ac). In invertebrates, plants, fungi, protists, bacteria, or archaea, on the other hand, this number is considerably larger, which tremendously increases structural complexity. The second important parameter is the connectivity (II), i.e., the regiochemistry of the glycosidic bond that connects the individual building blocks. In contrast to proteins and oligonucleotides, monosaccharides bear multiple glycosidic OH groups which all serve as possible linkage sites. Upon condensation of two monosaccharides, a new stereocenter emerges at the linkage site with two possible anomeric configurations (III). Finally, glycans are not necessarily assembled in a strictly linear fashion but may also exhibit one or more branching sites (IV).

All the above-mentioned structural parameters can lead to isomers—molecules that exhibit an identical atomic composition but differ in their structure. An analysis based on mass spectrometry (MS) therefore has inherent limitations to resolve and unambiguously identify the underlying molecular structure. Due to its enormous prevalence in proteomics^16−20^ and its exceptional sensitivity, however, MS is still a widely used and valuable tool for the analysis of glycans.^21−24^ The isomer problem is often addressed by coupling MS with orthogonal techniques.^25−28^ The most obvious of these combinations is certainly a separation of the analytes prior to MS analysis using liquid chromatography (LC)—again an approach that is extremely prevalent in other omics fields. However, even though of great use for certain applications, LC-MS-based approaches often struggle with the high polarity of glycans or the amphiphilic nature of glycoconjugates.

In recent years, a series of promising novel techniques directed at the specific needs of glycan analysis emerged.^29^ Many of these techniques are directly implemented within the mass spectrometer, enabling an improved duty cycle while still providing sufficient diagnostic potential. High energy activation methods such as electron-based dissociation (ExD)^30,31^ or ultraviolet photodissociation (UVPD)^32^ help to disentangle complex oligosaccharide structures based on more informative fragmentation patterns. Approaches such as ion mobility spectrometry (IMS) and gas-phase spectroscopy, on the other hand, are directly sensitive to the structure of the investigated molecule.^33−36^ Here we discuss recent developments in the structural analysis of glycans and glycoconjugates using MS-based techniques. As the present review is focused on the above-mentioned emerging techniques, traditional slow-heating methods such as collision-induced dissociation (CID) are addressed only briefly. The selection of references also reflects the growing importance of electrospray ionization (ESI) in the field, opposed to matrix-assisted laser desorption/ionization (MALDI) that dominated MS-based glycan analysis in the 1990s and early 2000s.

In the first part of the review (Section 2), general concepts and the fundamentals of each of the techniques will be described briefly, while the second part (Sections 3 to 8) addresses specific aspects of individual glycan and glycoconjugate classes. Sections dedicated to the different molecule classes may be read independently of each other, and the reader is encouraged to freely select those in their interest in any desired order.

## 2 Techniques

### 2.1 Mass Spectrometry

Natural glycans often occur in complex matrices and thus require isolation and purification prior to MS analysis. *N*- and *O*-glycans, for example, need to be released from glycoproteins by enzymatic or chemical methods for subsequent glycan analysis. Because glycan enrichment and purification strategies constitute a crucial prerequisite for the quality of MS analyses, they are briefly mentioned here to refer the reader to relevant literature. Commonly employed methods for glycan purification and enrichment include solid-phase extraction, liquid-phase extraction, chromatography, and electrophoresis.^37,38^ Chromatographic and electrophoretic methods routinely employed for glycan separation are reversed-phase high-performance liquid chromatography (RP-HPLC), hydrophilic interaction liquid chromatography (HILIC), porous graphitized carbon (PGC) chromatography, strong anion exchange (SAX) chromatography, and capillary electrophoresis (CE), which are discussed in more detail in excellent recent reviews.^28,39−43^ Solid-phase isolation can be combined with reversible or irreversible chemical coupling strategies, such as boronic acid capturing and hydrazide capturing, or exploits specific noncovalent interactions in lectin-capturing approaches.^44^ Following the extraction from natural sources, glycans are often derivatized to enhance ionization, detectability, and separation. Derivatization strategies for glycans are comprehensively discussed in the related literature.^45−47^

The present review focuses on developments accomplished in the past 15 years in the field of electrospray ionization–mass spectrometry (ESI-MS) based techniques for the structural characterization of glycans. Owing to the development of novel ion activation methods, the commercialization of ion mobility–mass spectrometry (IM-MS), and the introduction of gas-phase ion spectroscopy to the field of glycomics, the amount and specificity of structural information obtainable by MS-based methods has significantly increased in this period. The ESI process and various ion activation methods are discussed herein, followed by a brief description of IM-MS and the emerging gas-phase spectroscopic techniques in the infrared (IR) and ultraviolet (UV) region, which gain increasing importance for the structural analysis of glycans.

#### 2.1.1 Ionization

Mass spectrometry became widely applicable to the analysis of biomolecules with the advent of soft ionization techniques in the late 1980s. Today, the main ionization techniques employed for the transfer of intact glycans into the gas phase are matrix-assisted laser desorption/ionization (MALDI)^48^ and ESI.^49,50^ While around the turn of the millennium MALDI was regarded as the method of choice to generate gas-phase carbohydrate ions, in the past decade ESI has become the dominant ion source in MS-based glycan analysis, which is also reflected by the works selected herein. For the latest developments in glycan analysis with specific focus on MALDI, the reader is referred to excellent recent reviews dedicated to the subject.^51,52^ MALDI is often used to ionize glycans directly from tissue samples, whereas ESI enables ionization of glycans from solution and is thus compatible with liquid chromatography (LC) online coupling. Upon application of a high voltage between the emitter containing the sample solution and the entrance of the mass spectrometer, charge separation leads to the formation of a Taylor cone at the tip of the emitter, from which droplets are released. Evaporation of solvent and charge repulsion lead to a Coulomb explosion, which generates multiple smaller droplets from the original droplet. Finally, the ion is either released from the shrinking droplet (ion evaporation model), or the bare ion is obtained after complete evaporation of the solvent (charged residue model).^53^ Ion evaporation occurs for low molecular weight analytes, whereas the release of large analytes such as native (glyco-)proteins is more accurately described by the charged residue model.

In general, ESI yields even-electron molecular ions, which are typically protonated or metal adducts in positive ion mode. In negative ion mode, deprotonated or anion-adducted species are observed. The occurrence of multiple charge states is common for ESI, especially in large molecules. This can be beneficial for the detection of large molecules in a restricted *m*/*z* range. The charge state depends on the ESI conditions such as pH and the presence of salts or detergents. Glycans usually do not yield abundant ion signals due to their hydrophilicity and the absence of basic sites that can be protonated. The ion yield can be increased by derivatization such as permethylation, peracetylation, or labeling at the reducing end.^46^ Permethylation not only enhances the ionization efficiency but also stabilizes labile moieties such as sialic acid.

**Figure 2:**
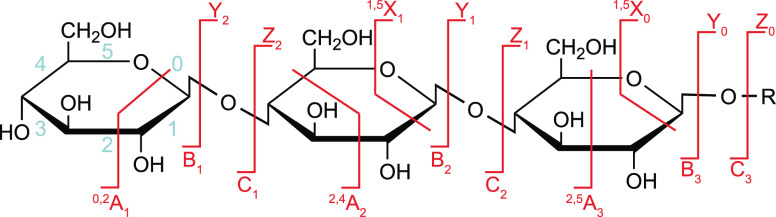
Domon–Costello nomenclature of carbohydrate fragmentation. A- and X-fragments result from cross-ring cleavages, whereas B- and C-fragments and their Y and Z counterparts originate from glycosidic cleavage between two monosaccharide units. The numbering of bonds in the sugar ring, which is indicated as superscript numerals in cross-ring fragments, is exemplarily shown for one monosaccharide. Figure is based on ref (54).

Once in the gas phase, glycans can undergo specific fragmentation upon activation. In principle, two fundamentally different types of cleavage can occur in oligo- and polysaccharides: glycosidic cleavage and cross-ring cleavage. The resulting fragments are commonly designated using the Domon–Costello nomenclature (Figure 2).^54^ The letters A, B, and C designate fragments containing the nonreducing end, whereas X-, Y-, and Z-ions are the complementary counterparts that retain the reducing end or the aglycone. A- and X-fragments result from cross-ring cleavage within the sugar ring of a monosaccharide, while the other ions are produced by glycosidic cleavage between two monosaccharides on either side of the glycosidic oxygen. The position of cleavage within the glycan chain is expressed by subscript numerals, whereas superscript numerals on cross-ring fragments indicate the bonds that are broken within the respective monosaccharide unit. The subscript numerals indicating the number of the glycosidic bond being cleaved depend on whether the fragments contain the nonreducing or the reducing end: in A-, B-, and C-type fragments, the glycosidic bonds are counted from the nonreducing end starting with 1, whereas the position of cleavage leading to X-, Y-, and Z-type fragments is counted from the reducing end or aglycone. The glycosidic bond between the glycan portion and the aglycone in glycoconjugates is by convention numbered 0. Glycosidic cleavage yields information about glycan sequence, whereas cross-ring cleavage is important to deduce information about linkage and branching. Glycosidic B-fragments are also particularly interesting for chemical synthesis of glycans as they are believed to occur as intermediates of S_N_1 reactions in solution, and their reactivity can be predicted by gas-phase studies.^55,56^ Glycosidic B- and C-fragments showed in some cases a memory of the stereoinformation on the glycosidic linkage in unprotected glycosides^57−61^ but not in protected glycosides.^62^

Apart from the intended formation of fragments yielding sequence and linkage information, unexpected rearrangement reactions of protonated glycans have been observed in the gas phase, which can lead to erroneous structural assignment. The process is often compared to peptide scrambling, a well-studied rearrangement reaction in proteomics.^63^ Misleading fragments that do not represent the original glycan structure are formed by so-called internal residue loss, during which a glycan fragment is lost from an internal position of the chain.^64,65^ At the same time, the monosaccharide at the tip of the chain migrates to adjacent or remote positions, which were not occupied in the original structure. This phenomenon of migrating hexoses was mainly observed for the deoxy sugar fucose. Typically, an asterisk in combination with the Domon–Costello nomenclature is used to denote the rearrangement products. Hexose migration must be considered for the structural investigation of protonated glycans and ammonium adducts, whereas metal adducts and negatively charged glycans do not undergo internal residue loss.^66^ Glycan rearrangement reactions will be discussed in more detail in Section 5.6.

#### 2.1.2 Ion Activation Methods

**Figure 3:**
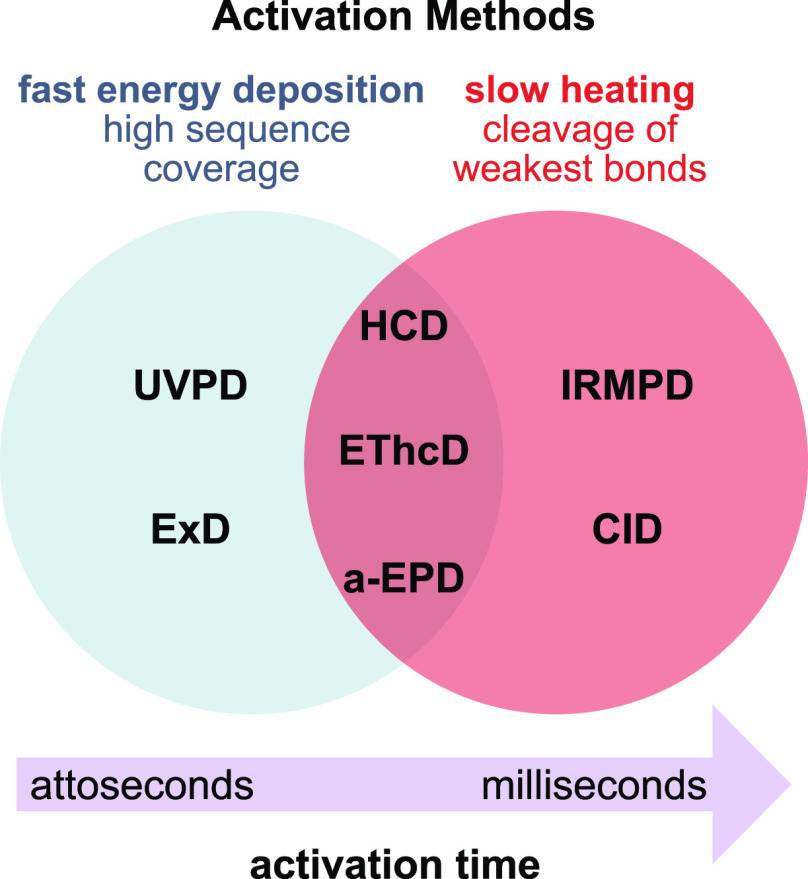
Time scale and specificity of different ion activation methods. Slow heating methods involve multiple activation events, leading to the cleavage of labile chemical bonds. Fast energy deposition occurs in a single activation step and yields complementary, site-specific fragments. UVPD = ultraviolet photodissociation; ExD = electron-mediated dissociation (including ECD, ETD, EDD, and EID/EED); HCD = higher-energy collisional dissociation; EThcD = electron-transfer/higher-energy collision dissociation; a-EPD = activated electron photodetachment; IRMPD = infrared multiple photon dissociation; CID = collision-induced dissociation.

With the advent of soft ionization techniques, which transfer intact ions into the gas phase with little or no fragmentation, a number of ion activation methods have been developed to allow for structural investigation of molecular ions by targeted fragmentation within the mass spectrometer.^67−69^ Ion activation methods differ in the total amount of energy deposited into the precursor ion, as well as in the activation time, i.e., the time scale of this energy deposition (Figure 3). Consequently, different methods lead to substantially different, often complementary, fragmentation mechanisms. In general, dissociation of isolated precursor ions in tandem MS can occur either via collision-, photon-, or electron-mediated activation. The most important ion activation methods with respect to the structural analysis of glycans and glycoconjugates will be briefly presented and compared among each other in the following.

**Figure 4:**
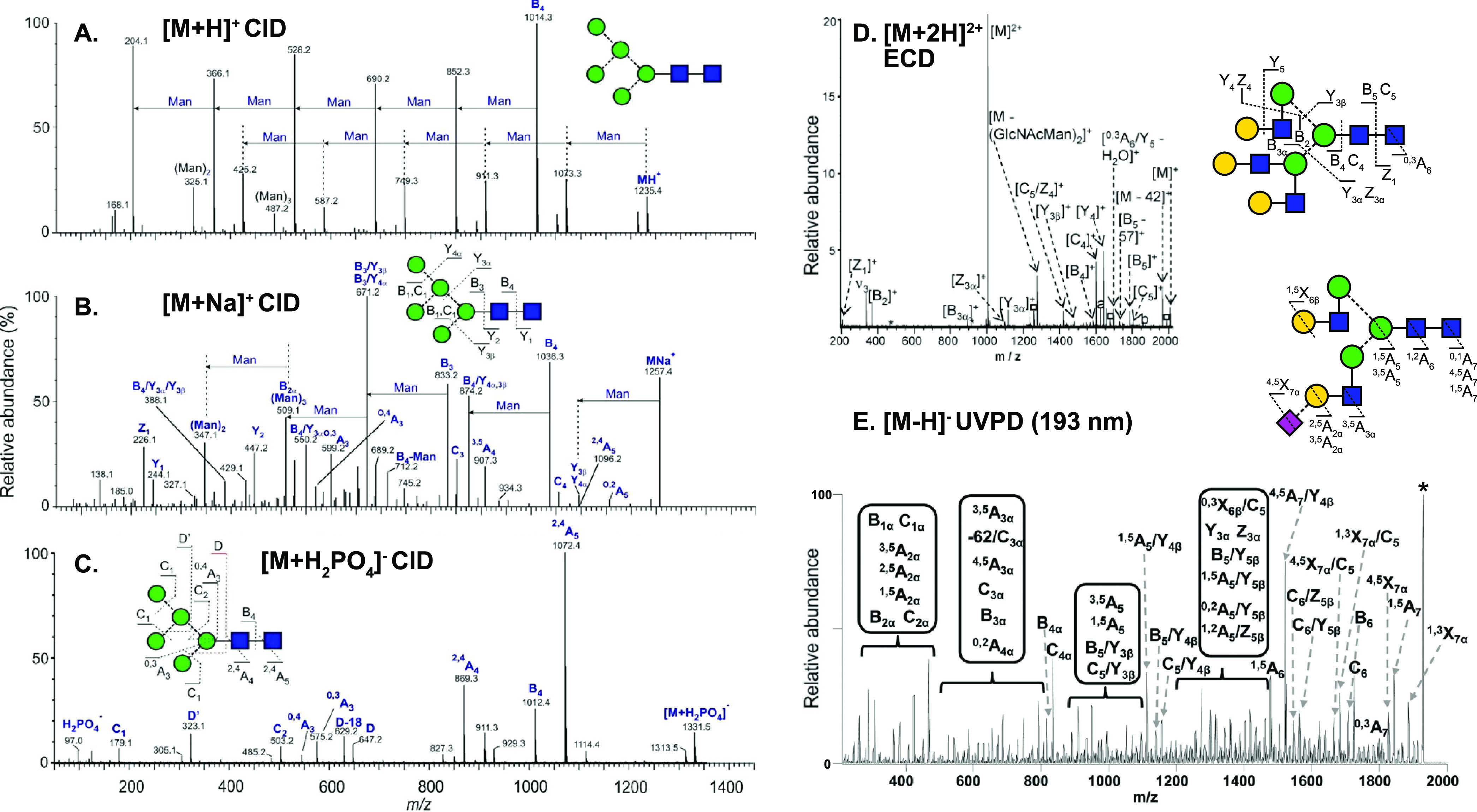
Influence of ion polarity and the ion activation method on the fragmentation pattern of *N*-glycans. (A) CID in positive ion mode mainly yields B-fragments from glycosidic cleavage. The abundance of cross-ring fragments can be increased by the coordination of metal cations (B) or by employing CID in negative ion mode (C). Figure reprinted with permission from ref (95). 2020 Copyright Wiley-VCH. (D) ECD yields both glycosidic and cross-ring cleavage. Figure adapted with permission from ref (85). Copyright 2007 American Chemical Society. (E) UVPD of *N*-glycans yields a wealth of fragments, which are informative but challenging to interpret. Only cross-ring fragments are shown in the accompanying structure for clarity. Figure adapted with permission from ref (76). Copyright 2011 American Chemical Society.

Collision-mediated activation was the first activation method to be employed and is still the most widely used method in modern tandem MS instruments. Precursor ions are accelerated by an electric field and undergo inelastic collisions with a heavy neutral gas, which leads to partial conversion of the kinetic energy into internal energy. Collision-induced dissociation (CID)^70^ employs collision energies in the eV range and usually allows for long activation times during which multiple collisions of the precursor ion and a target gas occur. CID is thus a slow heating method^71^ with sequential increase of internal energy until the weakest bonds break. Those are usually glycosidic bonds in unmodified glycans or labile glycan modifications such as sulfates (Figure 4A–C). Higher-energy collisional dissociation (HCD)^72^ employing collision energies in the keV range results in single or few collisions and fast ion activation. The technique is less established but can yield a multitude of fragments from direct bond cleavage that are inaccessible by CID, such as abundant fragmentation of both the glycan and peptide backbone in glycopeptides.^73^

Photon-mediated fragmentation induces ion activation by irradiation with photons from different regions of the electromagnetic spectrum. Infrared multiple photon dissociation (IRMPD)^74^ is a slow heating method based on the sequential absorption of IR photons, which are usually provided by a CO_2_ laser (λ = 10.6 μm, 117 meV). After each photon absorption event, the increase in internal energy in the precursor ion is redistributed over all vibrational modes by intramolecular vibrational redistribution (IVR). After multiple absorption–IVR cycles, the dissociation threshold is reached, and the weakest bonds are cleaved as in CID.^71^ Using a tunable light source instead of a fixed wavelength, IRMPD can be employed to record gas-phase IR spectra, as discussed in more detail in Section 2.3. Ultraviolet photodissociation (UVPD)^75^ employs UV photons of wavelengths between 10 and 400 nm, which are sufficiently energetic for single-photon dissociation. UVPD is thus a fast activation method occurring via electronic excitation of the precursor ion. It yields abundant fragmentation including A- and X-fragments resulting from cross-ring cleavage and site-specific cleavage on sialic acids (Figure 4E).^32,76^ A prerequisite for UVPD is a suitable UV-absorbing chromophore on the precursor ion. UVPD of multiply charged anions can trigger a radical fragmentation mechanism by electron detachment (activated electron photodetachment; a-EPD).^77^

Electron-mediated ion activation methods (ExDs) are classified as fast activation methods inducing specific bond cleavage and are particularly important for structure analysis of glycoconjugates but also free glycans.^78^ They include electron capture dissociation (ECD),^79^ electron transfer dissociation (ETD),^80^ electron detachment dissociation (EDD),^81^ electron-induced dissociation (EID),^82^ and electronic excitation dissociation (EED). Except for EID and EED, these methods are only applicable to multiply charged cations (ECD and ETD) or anions (EDD), as they induce charge state reduction by adding or detaching an electron, respectively. ECD and ETD induce very similar fragmentation mechanisms but differ by the technical implementation of the initial electron transfer and thus require different instrumentation. ECD employs a low-energy electron source and requires the magnetic fields of an FTICR cell for trapping electrons. ETD, on the other hand, is also compatible with linear and 3D quadrupole ion traps because trapped electrons are not employed for ion activation. Instead, electron transfer is mediated by stable odd-electron anions generated in a separate ion source. Both ECD and ETD transform multiply charged cations into radical cations, which subsequently fragment. Importantly, the fragmentation mechanism depends on the location of the captured electron rather than dissociation barriers. As a result, weak bonds and even noncovalent interactions that are preferentially cleaved in CID or IRMPD can remain intact. Therefore, ECD and ETD are extensively harnessed for the investigation of labile post-translational modifications in peptides and proteins, such as *O*-glycosylation,^83^ but also for sequence analysis of oligosaccharides, which can yield informative cross-ring fragments upon electron capture (Figure 4D).^84,85^ EDD is the negative ion analogue to ECD, transforming multiply charged anions into radical anions by electron detachment, employing electrons of moderate kinetic energy. The method is particularly useful for the analysis of sulfated oligosaccharides such as GAGs, which yield informative fragments from both glycosidic and cross-ring cleavages that locate sites of sulfation.^86^ However, EED, as well as ECD, rely on FTICR instrumentation because electrons and ions must be trapped simultaneously. Contrary to the ExD methods described before, EID and EED can be applied to singly charged positive or negative ions and are thus suited for the investigation of small glycoconjugates such as glycopeptides and -lipids that are only observable as singly charged species.^87^ EED results in extensive cross-ring cleavage and therefore holds much potential for linkage analysis.^88^

A drawback of many ExD methods is their low fragmentation efficiency, making long reaction/interaction periods necessary to achieve sufficiently intense fragment ion peaks. Since a single successful ion–electron encounter usually deposits enough energy to induce fragmentation, ExD activation times are short. However, such successful ion–electron encounters are of low probability due to the small interaction cross sections, leading to inefficient fragmentation. Ion–electron interaction periods ranging from hundreds of milliseconds to seconds—significantly longer than those employed in CID—have hindered the straightforward online coupling of many ExD methods to chromatographic and electrophoretic separations. As electrostatic repulsion between polyanions and electrons renders EDD particularly inefficient, the development of LC-ExD-MS/MS workflows for the analysis of acidic glycans as negative ions proved to be especially challenging. However, the introduction of NETD, a technique relying on more efficient anion–cation interactions and requiring shorter interaction periods, opened the way recently for such multidimensional workflows.^89,90^

Several mass spectrometer designs allow for multiple fragmentation of ions in time or in space. Multistage MS or MS^*n*^ in time is provided by mass spectrometers based on ion trapping, which can repeatedly isolate and fragment ions. This approach is useful for detailed structure elucidation of fragments^91^ and can reveal information about glycan branching patterns.^92^ Due to considerable ion losses in each round of isolation and fragmentation, a sufficient precursor ion intensity is required. Different ion activation methods can also be combined in space to yield complementary information in a single experiment.^93^

In summary, ion activation methods can be classified either by the source of activation (collision, photon, electron) or by the activation time, which is long for multiple discrete activation events or short if the activation occurs quickly relative to the unimolecular dissociation. For example, IRMPD and UVPD are both classified as photon-mediated activation methods but are located at opposite sites on the activation time and specificity scale. In general, long activation times lead to fragmentation according to bond dissociation energies, whereas short activation times induce direct bond cleavage before the energy is redistributed over the whole molecule. Each ion activation method has its own area of application, and their combination can yield a more comprehensive picture of complex glycan and glycoconjugate structures.

#### 2.1.3 Ion Polarity

The nature of fragments generated in tandem MS is not only dependent on the choice of ion activation method but also crucially influenced by ion polarity and the type of coordinating cations or anions.^94,95^ In positive ion mode, CID of protonated oligosaccharides mainly yields B-fragments resulting from glycosidic bond cleavage (Figure 4A). Coordination of monovalent and divalent metal cations was shown to increase the abundance of diagnostic cross-ring cleavage in positive ion mode in numerous studies (Figure 4B).^69^ Contrary to protons, metal cations can coordinate to multiple oxygen atoms simultaneously, and their site of coordination influences the type of fragment ions. Metal cations coordinating to the ring oxygen were suggested to prevent glycosidic cleavage by localizing the oxygen’s electrons while not impeding cross-ring fragmentation.^96^ Negative ion mode CID provides details on linkage by yielding abundant A-fragments from cross-ring cleavage (Figure 4C).

In order to obtain more comprehensive linkage information, orthogonal techniques can be coupled to MS to allow for in-depth structural investigation of glycans.^35^ IMS and gas-phase action spectroscopy coupled to MS are increasingly employed for glycan analysis and will be detailed in the following.

### 2.2 Ion Mobility Spectrometry

Ion mobility spectrometry (IMS) is a gas-phase electrophoretic separation technique, widely employed as a stand-alone method for the detection of drugs, explosives, and chemical warfare agents.^97−99^ It has a long history as part of ion mobility–mass spectrometry (IM-MS) couplings, with applications in fields as diverse as molecular physics and structural biology.^100−114^ Since the commercialization of the first integrated instruments, IM-MS has gained remarkable popularity in bioanalytical chemistry^115−126^ and become a key element of the glycomics toolbox.^23,29,34,35,127−130^ Glycan isomers often exhibit identical fragment ion spectra, impeding their tandem MS-based distinction, while the presence of multiple isomers in a mixture calls for efficient separations. IMS proved to be extremely useful for the distinction, postionization separation, and relative quantification of glycan isomers, helping to overcome key challenges associated with MS-based glycan analysis.^131−134^ In addition, as comprehensive IMS analyses take place generally on the millisecond time scale, they fit perfectly between condensed-phase separations and fast MS experiments, further increasing the peak capacity of multidimensional workflows.^135^

In general, IMS separates ions based on differences in their gas-phase mobilities *K*, a transport property related to the ions’ mass, charge, size, and shape. *K* is also influenced by the nature and number density of the buffer gas and by the effective temperature at high reduced fields, as will be discussed later in relation to field asymmetric IMS. As they traverse a suitable gas-filled cell under the influence of an electric field, ions undergo binary collisions with the neutral gas particles. Larger, extended species collide more frequently, reaching lower velocities upon their electrophoretic motion than more compact ions. This simplified picture reveals the aptitude of IMS to separate isomers and conformers, species having identical *m*/*z* ratios but often differing in size and shape.

Within the low-field limit, ion–neutral collisions are essentially thermal, and *K* is basically independent of the electric field strength *E*:
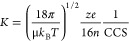
1Equation 1 is the fundamental low-field ion mobility equation, also called the Mason–Schamp equation.^136−138^ Here, μ is the reduced mass of the ion–neutral collision complex, *k*_B_ the Boltzmann constant, *T* the buffer gas temperature, *z* the ionic charge state, *e* the elementary charge, *n* the buffer gas number density, and CCS the rotationally averaged collision integral, often referred to as the collision cross section. CCSs are related to the size and shape of the collision partners but also depend on interaction potentials.^101,139,140^ They serve as effective areas, generally expressed in units of Å^2^, reflecting momentum transfer between the colliding particles.^141^ The larger the CCS of an ion–neutral pair, the more efficient the momentum transfer between the ion and the gas particles in IMS. CCSs are independent of gas pressure or number density and vary generally less with temperature than mobilities, making them suitable molecular descriptors. They are comparable across different IMS platforms and can be readily stored in databases, facilitating the identification of analytes. Besides experimental determination in suitable IMS experiments, CCSs may also be calculated by computational methods, addressed in more detail at the end of this section.

**Figure 5:**
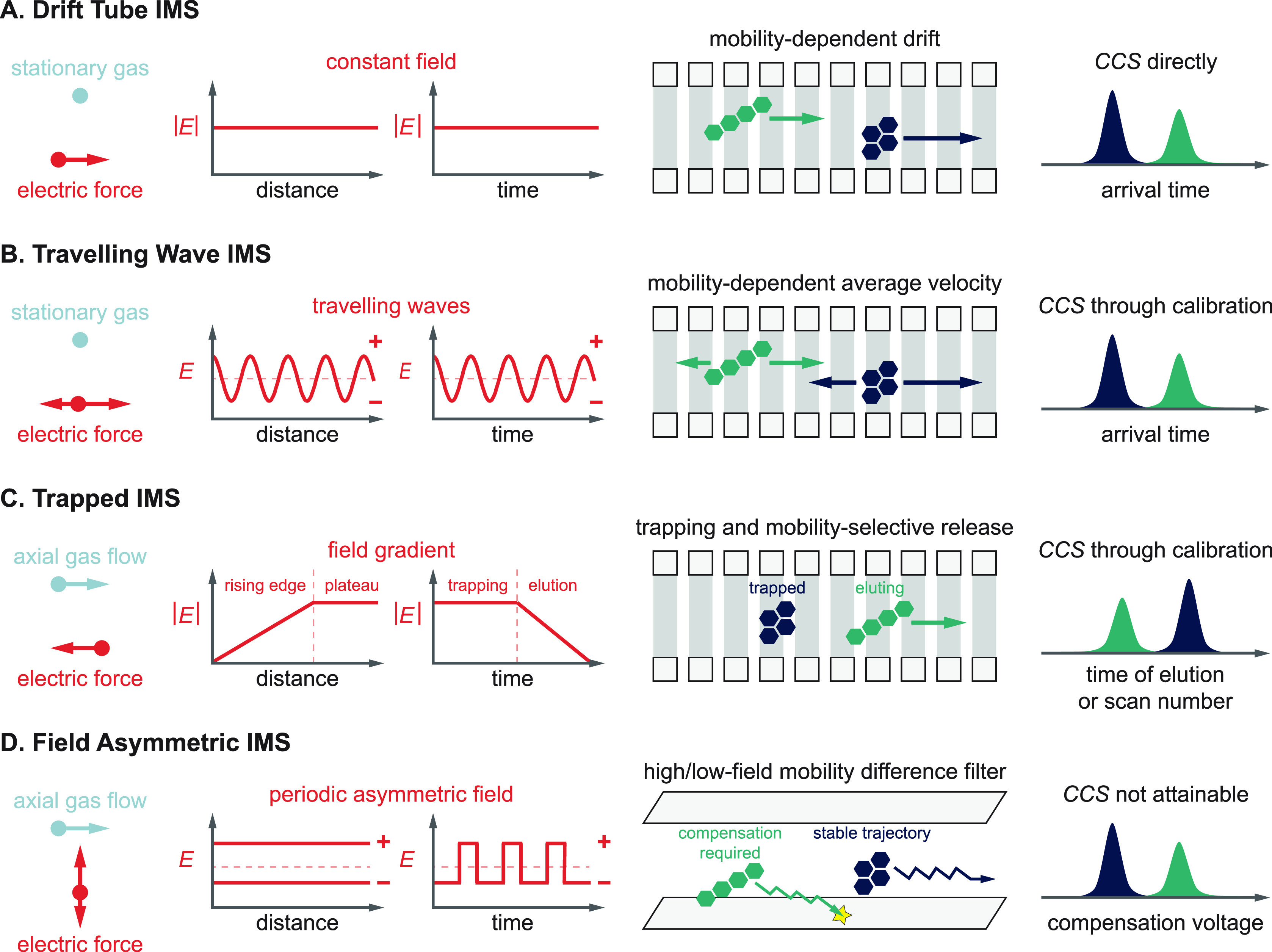
Overview and basic principles of ion mobility spectrometry techniques commonly applied in glycan analysis.

Various IMS techniques have been developed and commercialized in recent years that efficiently harness electric forces for separating ions in gases.^142−144^Figure 5 provides an overview of the methods applied most successfully in glycan analysis, accompanied by a brief description of each technique below.

#### Drift Tube Ion Mobility Spectrometry (DTIMS)

Ions are propelled through a stationary buffer gas by a constant electric field, invariable in both space and time. Ions with higher mobilities traverse the drift cell faster and reach the detector at shorter arrival times, making DTIMS a time-dispersive method.^145^ In the constant electric fields applied, drift velocities are directly proportional to mobilities, enabling determination of the latter directly from first principles. When experiments are performed within the low-field regime, obtaining CCSs from mobilities using eq 1 is straightforward. To date, the stepped-field method using DTIMS/IM-MS represents the most accurate way of determining *K* and CCS values experimentally, with expanded uncertainties reported as low as 0.5%.^146^ Although these features make the technique very attractive, enhancing resolving power requires increasingly long pathways and short injection pulses combined with high DC voltages, which may become impractical or difficult to achieve beyond a certain limit.^144^

#### Traveling Wave Ion Mobility Spectrometry (TWIMS)^147−149^

TWIMS is a time-dispersive method where ions are driven through a gas-filled cell by the eponymous traveling potential waves. In brief, the approximately sinusoidal waves traverse the mobility cell with velocities generally between 100 and 500 m s^–1^, while the ions follow their movement in a mobility-dependent manner. Analytes with higher mobilities are able to “surf” longer on each wave, experiencing fewer discrete roll-over events and reaching higher average velocities. The electric field itself changes sign between the leading and trailing edges of each electric potential wave, causing the ion velocity vectors to fluctuate. This results in a characteristic “*n* steps forward, one step back” motion of the analytes. Despite remarkable improvements in TWIMS theory,^150,151^ to date no complete and general analytical model has been developed that would allow for inferring mobilities directly from transit times under a broad range of practical operating conditions. Thus, obtaining CCSs from TWIMS measurements generally requires calibrant ions whose CCS values had previously been determined in low-field DTIMS experiments. An advantage of TWIMS technology over DTIMS is the flexibility it enables in instrument geometry: cyclic arrangements or structures for lossless ion manipulations (SLIMs) with serpentine routes provide extremely long separation pathways within compact architectures.^152,153^ Such instruments are capable of achieving resolving power values currently unattainable in linear drift tubes, allowing for the separation of glycan anomers in the gas phase.^154,155^

#### Trapped Ion Mobility Spectrometry (TIMS)^156−158^

TIMS represents an alternative concept to time-dispersive methods for separating ions according to their gas-phase mobilities. Here, ions are held stationary along a linear electric field gradient by two counteracting forces: a fast axial gas flow (>100 m s^–1^) drags the ions toward the exit, while the electric field propels them in the opposite direction. The position along the axial field gradient where the two forces exactly balance each other, focusing the ions into steady-state zones, depends on the ions’ mobility. Ions with high *K* are trapped close to the entrance (lower fields), while species with low *K* occupy the region closer to the exit (higher fields). Having reached their mobility-dependent stationary positions, the ions are eluted selectively by gradually decreasing the magnitude of the electric field. The higher their mobility, the later the ions elute, causing the species to reach the exit in an order of increasing mobility. Due to the high linear gas velocities in the tunnel, the effective path length covered by ions during their elution can be orders of magnitude higher than the physical dimensions of the TIMS tunnel, enabling outstanding resolving powers (*R*_P_ > 250) to be reached in a compact device.^144^ Although TIMS in principle enables the direct determination of *K* and CCS values, measuring the gas velocity and pressure with sufficient accuracy is extremely difficult. Thus, establishing calibration curves using ions of known CCS is preferred in practice.^159^ Finally, as radial focusing is achieved using radiofrequency fields in the TIMS tunnel, ion heating during long trapping times is worth being considered when analyzing labile species.

#### Field Asymmetric Ion Mobility Spectrometry (FAIMS)^160−162^

In all three techniques above, ion transport and separation are dictated by the absolute value of mainly low-field mobilities. In stark contrast, FAIMS devices filter ions based on the shift in their mobility between low and high fields. The dependence of an ion’s mobility on the reduced field strength *E*/*n* is considered negligible below the low-field limit but becomes significant once this limit is exceeded. To harness this dependence, FAIMS employs a periodic, highly asymmetric field perpendicular to a gas flow that propels ions uniformly toward the exit of the planar device. The field changes between positive and negative values, generating high-field conditions for a shorter time and low-field conditions at the opposite polarity for a proportionally longer time (the time integrals of the two fields are equal). This causes the ions to oscillate along the field axis, experiencing deflection toward the top or bottom electrode if the low- and high-field mobilities are different. To force ions on stable trajectories, a compensation voltage is applied between the electrodes. Scanning this DC voltage enables ions with different mobility shifts to traverse the cell without hitting the electrodes and pass through the exit. Being essentially a mobility shift filter, FAIMS does not allow for comprehensive analysis in a single run. Instead, it can provide a continuous beam of a selected species at 100% duty cycle, making it highly compatible with slower mass analyzers, such as FTICR-MS instruments.^163^ Although FAIMS does not allow for inferring absolute *K* or CCS values from the experiments, it is superior to the above three IMS techniques in terms of orthogonality to MS. Shifts in mobilities upon moving from low to high fields are difficult to predict, and they show generally weaker correlation with *m*/*z* ratios than low-field mobilities.

Although experimentally determined CCSs are highly useful to distinguish and identify analytes, they ultimately reduce molecular structures to a single value: an effective area. These areas are linked to the overall shape of ions but do not carry direct, atomic level information about the underlying molecular structure. Without prior knowledge and based merely on experimentally determined CCSs, it is extremely difficult—if not impossible—to tell unambiguously whether two separated isomers differ in their conformation, configuration, or constitution. To obtain atomic level structural details with the help of CCSs, complementing theoretical approaches are indispensable. The strategy, in principle, is simple: experimentally determined CCSs are compared to those calculated for structural candidates generated by computational methods, and the model structures are evaluated on the basis of CCS agreement. This approach proved to be very helpful for the structural analysis of charged clusters^164−166^ and has been successfully adopted for biomolecular ions.^167,168^

To calculate CCSs, the first, computationally less expensive methods treated the colliding partners as hard spheres, ignoring details of the interaction potentials.^164,169^ The development of the trajectory method (TM) applying realistic interaction potentials put CCS calculations on a more solid physical basis, albeit at the expense of significant computational cost.^139^ Since then, continuous improvements in theoretical methods and computational tools have manifested in increasingly fast and accurate CCS calculations in atomic and molecular gases.^170−179^ Today, the main bottleneck for glycans is not the calculation of CCSs for given model structures with satisfying accuracy (at least in common buffer gases) but the generation of reliable structural candidates. It requires advanced quantum chemical methods, most prominently density functional theory calculations.^35^ As isolated carbohydrates represent an immense challenge in theoretical chemistry, Section 2.5 is dedicated entirely to the subject, focusing on electronic structure calculations and their merging with methods to explore the vast conformational space of glycans.

### 2.3 Gas-Phase Infrared Spectroscopy of Mass-Selected Ions

**Figure 6:**
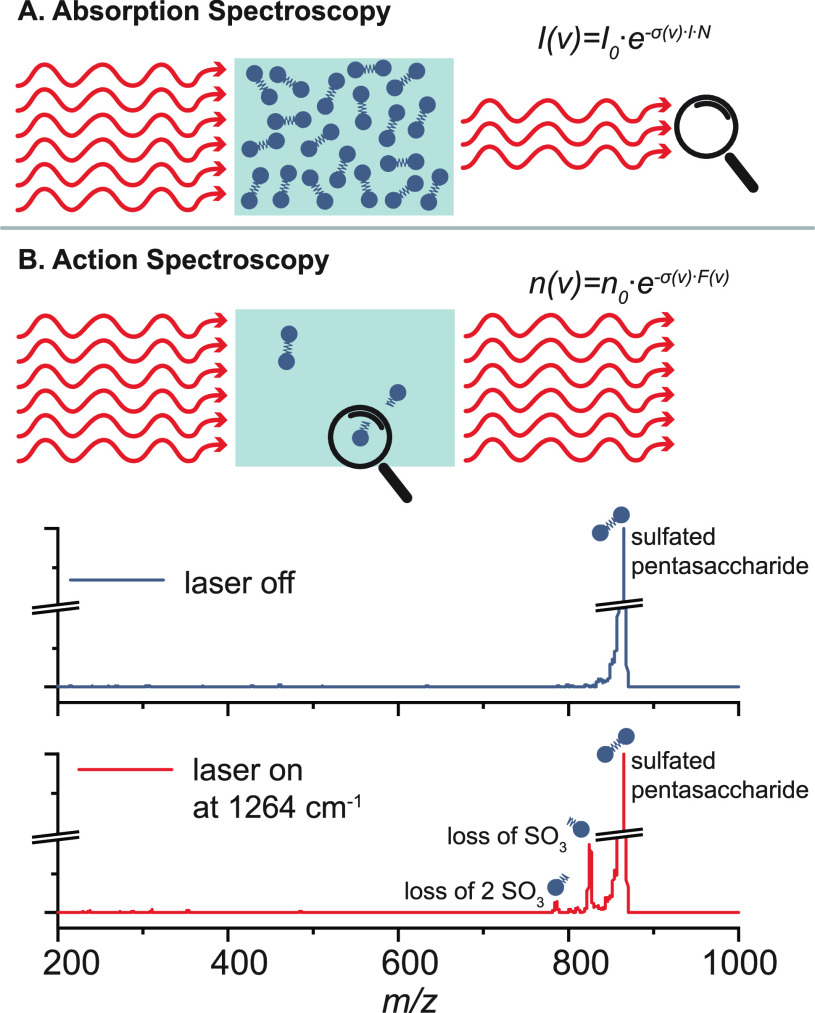
Direct absorption vs action spectroscopy. (A) Concept of direct absorption spectroscopy. The Lambert–Beer law relates the intensity of transmitted light *I* at a specific frequency ν to the intensity of the incident light *I*_0_, the absorption cross section σ, the path length *l*, and the particle density *N*. (B) Concept of action spectroscopy (upper panel). The equation is derived from the Lambert–Beer law and relates the number of unaffected ions *n* at a specific frequency ν to the number of precursor ions *n*_0_, the absorption cross section σ(*ν*), and the photon fluence *F*(*ν*). IRMPD time-of-flight mass spectra of a *m*/*z*-selected highly sulfated pentasaccharide without (middle panel) and with (lower panel) resonant IR irradiation. Two fragments with a sequential loss of neutral SO_3_ are observed upon multiple photon dissociation at 1264 cm^–1^.

Infrared (IR) spectroscopy is a powerful tool used to identify unknown molecules and deduce information on their structure, such as functional groups, intra- and intermolecular interactions, as well as molecular conformations. Electromagnetic radiation in the IR range can excite molecules if the frequency of the incident radiation is in resonance with IR active vibrational transitions. IR radiation expands from the edge of the visible spectrum to the microwave range and can be further divided into the higher energy, near IR (>4000 cm^–1^), mid-IR (4000–400 cm^–1^) and lower energy, far IR (400–10 cm^–1^) regime. Fundamental vibrations are typically found in the mid-IR range. Classical IR spectroscopy techniques are based on direct absorption spectroscopy, which measures the attenuation of light after passing through a solid, liquid, or gaseous sample. The absorbance as a function of the frequency is derived from the Lambert–Beer law. The concept of direct absorption spectroscopy is depicted in Figure 6A. For *m*/*z*-selected ions in the high vacuum of a mass spectrometer, the sample density is usually limited to 10^6^ singly charged ions per cubic centimeter due to Coulomb repulsion and the resulting space-charge limit.^180^ The attenuation of light after passing through the low-density sample is so low that it is difficult or even impossible to measure. Therefore, vibrational spectroscopy of ions in the gas phase has evolved and is today typically performed as action spectroscopy, as addressed in recent reviews.^181,182^ Here, the absorption of photons is measured indirectly by following an action, e.g., the dissociation of the intact ion or electron detachment (see Section 2.4). A schematic concept of action spectroscopy can be found in Figure 6B. The number of unaffected ions *n* at a specific frequency ν can be expressed as a function of the number of precursor ion *n*_0_, the absorption cross section σ(ν), and the photon fluence *F*(ν) in the following equation:^181^
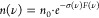
2The challenge is that IR light is usually much lower in energy than the threshold to dissociation; therefore, the analytes must have a low barrier to dissociate, or the laser system must be intense enough to enable multiple photon absorption. With the advance of tunable and powerful laser systems such as IR free-electron lasers (FELs)^183−185^ and benchtop laser systems such as optical parametric oscillators and optical parametric amplifiers (OPO/OPA) in the early 2000s, the interest in gas-phase IR spectroscopy of *m*/*z*-selected ions for their structural analyses started growing. Early works by the Simons group^186,187^ investigated small, neutral glycans in a UV-IR double-resonance experiment from a free jet expansion. Limited to the presence of UV chromophores and the harsh ionization method, the method was only applicable to small glycans. Combined with ESI sources, the field of IR spectroscopy finally opened up for the investigation of larger glycan ions.^188−190^

#### Infrared Multiple Photon Dissociation (IRMPD) Spectroscopy

One of the most widely used types of IR action spectroscopy is IRMPD spectroscopy, which induces fragmentation of weakly bound clusters or ions with energetically low fragmentation barriers upon irradiation.^181,182^ An IR spectrum is recorded by measuring the fragmentation yield as a function of the wavelength. The IRMPD mechanism is a nonlinear process involving the sequential absorption of a large number of IR photons (typically tens to hundreds) in which the energy of a single photon is distributed throughout the ion via IVR; i.e., the original vibrational mode relaxes via anharmonic coupling to vibrational background states. The sequential absorption of single photons, hence, takes place via the same fundamental vibrational level; yet, the internal energy of the ion rises with vibrational excitation until the dissociation threshold is reached and the ion fragments. With the IVR-coupled excitation process, the vibrational excitation is randomized throughout the ion so that the fragmentation occurs statistically, and usually the weakest bonds dissociate. For the above reason, IRMPD fragmentation patterns closely resemble those obtained by CID. Inherent to the process, red-shifting of absorption bands and spectral congestion can leave an imprint on the IRMPD spectrum. Additionally, glycans typically populate a large number of coexisting conformers at room temperature due to their conformational flexibility, which further increases spectral congestion and limits IRMPD to mostly mono- and disaccharides.^188,191−193^

Various designs of IRMPD instruments are published, yet in the most basic approach an interaction region with electrodynamic ion optics is sufficient to radially define the ion cloud and achieve an efficient overlap of the ions with the laser. Ion storage proves beneficial for slow dissociation processes so that the ions can be irradiated for a longer time. Online coupling of IRMPD with LC-MS workflows is challenging because the time required to record an IRMPD spectrum usually exceeds 10 min, which is not compatible with the time scale of LC peaks. However, efforts are underway to generate IRMPD spectra from chromatographically separated glycans.^194^

#### Messenger-Tagging Spectroscopy

Another approach that does not require multiple photon excitation steps is messenger-tagging spectroscopy which follows the detachment of a weakly bound, noninteracting messenger atom or tag upon resonant irradiation. Typical tags for the investigation of glycans are helium and nitrogen, and for other (bio)molecules the use of argon and hydrogen has been explored. Contrary to IRMPD spectroscopy, the IVR-mediated detachment mechanism of the tag in messenger-tagging spectroscopy is a linear single-photon process overcoming red-shifting and reducing spectral congestion. Furthermore, the power of the tunable IR laser required for messenger-tagging spectroscopy is significantly lower and thus renders the approach more compatible with benchtop lasers. Prerequisites for the attachment of a tag are temperatures close to the boiling point of the tag. Coincidently, this limits the numbers of glycan conformers present, therewith further reducing spectral broadening. Several IR spectroscopic studies on glycans, small building blocks up to oligosaccharides,^189^ using messenger-tagging spectroscopy are reviewed in the respective chapter of their glycan classes.

**Figure 7:**
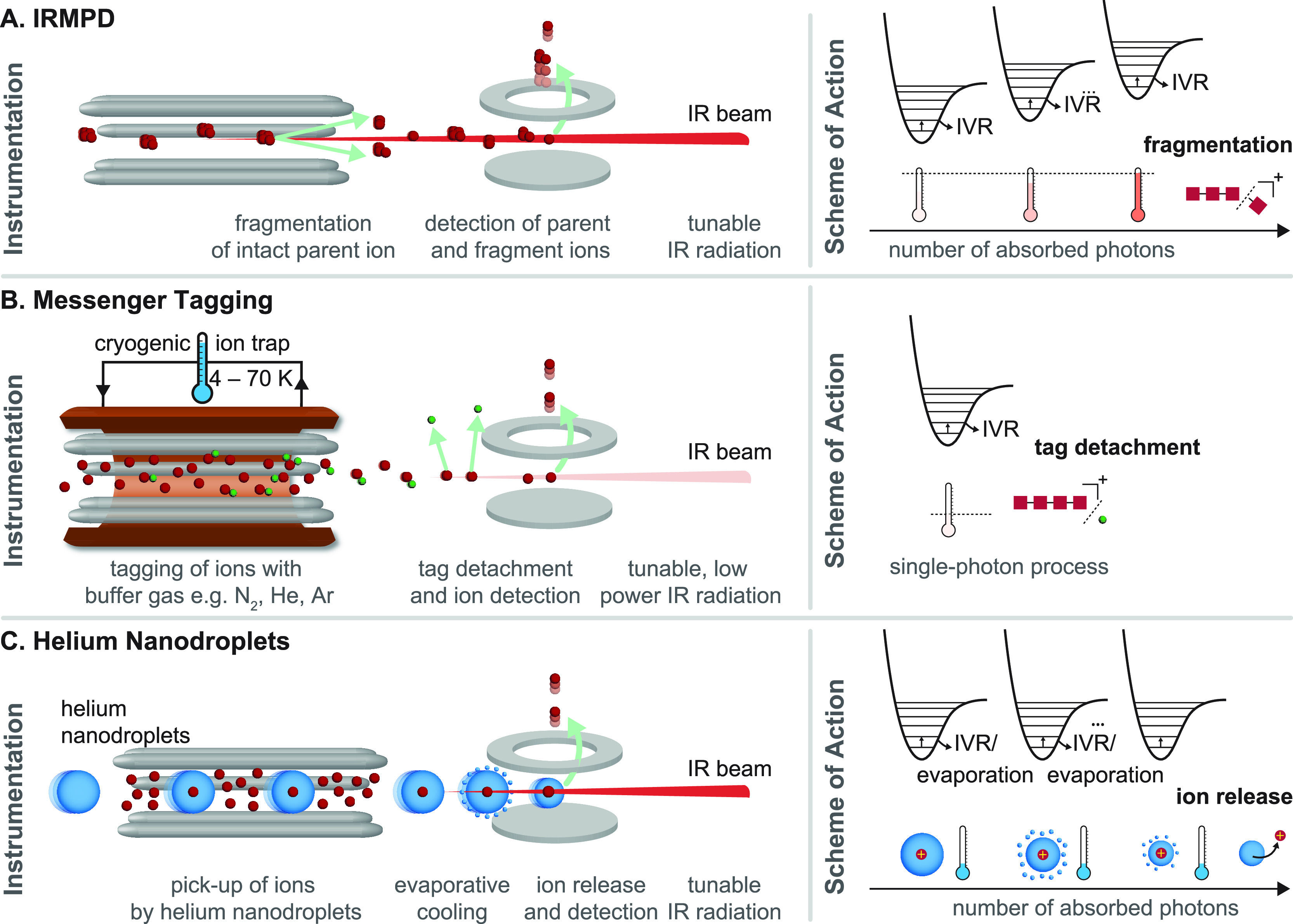
Comparison of types of IR action spectroscopy. (A) Instrumentation and scheme of action for infrared multiple photon dissociation (IRMPD) spectroscopy. With resonant irradiation, multiple IR photons excite the intact parent ion until the fragmentation threshold is reached. Fragment and parent ions are detected. (B) Instrumentation and scheme of action for messenger (tagging) spectroscopy. Ions are tagged with buffer gas atoms or molecules, e.g., N_2_, He, or Ar, in a cryogenic ion trap. With resonant irradiation typically with a single photon, the tag is detached, and the bare ion is detected. (C) Instrumentation and scheme of action for cryogenic spectroscopy in helium nanodroplets. The ions are picked up by helium nanodroplets in an ion trap and cooled to 0.4 K. With resonant irradiation, the ion is excited and immediately cooled again by evaporative cooling. After several iterations, the ion is released from the nanodroplet and detected.

Already in the 1980s, messenger-tagging spectroscopy was developed as a concept using a supersonic jet expansion followed by electron ionization or corona discharge. With the low temperatures in the supersonic jet, ions or clusters formed weakly bound complexes with coexpanded hydrogen. The detachment of the hydrogen tag upon irradiation was detected in a quadrupole mass analyzer (Figure 7). Since the mid-1990s, the development of cryogenic ion traps allowed the combination of this technique with soft ionization sources.^195,196^ The ions are transferred to the cryogenic ion trap, cooled by collisions, and eventually tagged with buffer gas at temperatures between 3 and 70 K. Various designs for cryogenic ion traps serve different purposes, such as temperatures below 3 K, long storage times, spatial spread of the ion cloud, or space focusing of ions with the ejection from the trap. As the first designs, multipole ion traps were developed,^197−199^ followed by planar multipole ion traps^200−202^ and ring ion guide traps.^199^ It was recently published^203^ that a wire quadrupole ion trap, with a linear quadrupole geometry where each of the rods is approximated by six copper wires, reaches temperatures below 3 K and is attached to a commercial mass spectrometer. In all recently published designs, the interaction region with the laser is a cryogenic ion trap.

#### IR Action Spectroscopy in Helium Nanodroplets

IR action spectroscopy in helium nanodroplets is currently applied in basic research only, yet the spectral quality which is achievable for glycan ions sets new benchmarks in the field.^190^ The superfluid helium nanodroplets are produced from a precooled reservoir of helium with the opening of a valve into the mass spectrometer, subsequent evaporative cooling, and formation of nanodroplets of a defined size (typically 10^5^ helium atoms). Before irradiation, the trapped ions are picked up by the traversing helium nanodroplets and cooled to the equilibrium temperature of the droplet of 0.37 K. Upon irradiation with a resonant photon, the ion is vibrationally excited and immediately cooled again to its ground state by evaporation of helium from the shell of the droplet. After several iterations, the ion is eventually released from the droplet and detected background-free. With this approach, a unique resolving power and distinct spectral fingerprints have been recorded for various classes of glycans up to pentasaccharides.^55,204,205^

### 2.4 Gas-Phase Ultraviolet Spectroscopy of Mass-Selected Ions

Besides IR spectroscopy that probes vibrational transitions, molecular ions may also be studied using UV spectroscopy inside a mass spectrometer.^206^ UV spectra reflect the electronic structure of analytes. They are sensitive to the local environment of chromophores and thereby to the constitution, configuration, and conformation of molecules. When performing UV spectroscopy on isolated ions, one needs to rely on action spectroscopic approaches, similarly to gas-phase IR spectroscopy discussed above. On one hand, the attenuation of UV radiation by a low-density ion cloud of absorbing species is extremely weak, which makes it unfeasible to record spectra by measuring changes in light intensity. On the other hand, absorption of energetic UV photons leads to electronic excitation and initiates a variety of traceable processes in isolated ions. By monitoring such a photoinduced process, serving as the *action*, one can readily detect photon absorption. Following electronic excitation, the ions can undergo various relaxation pathways, often leading to dissociation or also to electron photodetachment (EPD)^207^ in polyanions. Because both UVPD and EPD are accompanied by changes in the *m*/*z* ratio, they can be readily monitored by mass spectrometers in action spectroscopy schemes. Plotting the UVPD and/or EPD yield as a function of irradiation wavelength yields UV spectra for *m*/*z*-selected ions.

UVPD and EPD are usually single-photon processes, enabling experiments to be performed on cold ions using cryogenic ion traps. Cooling analytes can greatly reduce thermal broadening, leading to less congested electronic spectra, often with vibrational resolution.^208^ UV ion spectroscopy requires bright, tunable UV sources, with optical parametric oscillators (OPOs) and dye lasers being commonly applied. UV optical spectra of biomolecular ions are usually recorded in ranges between 200 and 400 nm (3.1–6.2 eV or 25 000–50 000 cm^–1^). For analytical purposes, simpler one-color experiments are more suited, requiring only a single laser and less sophisticated optical layouts.

**Figure 8:**
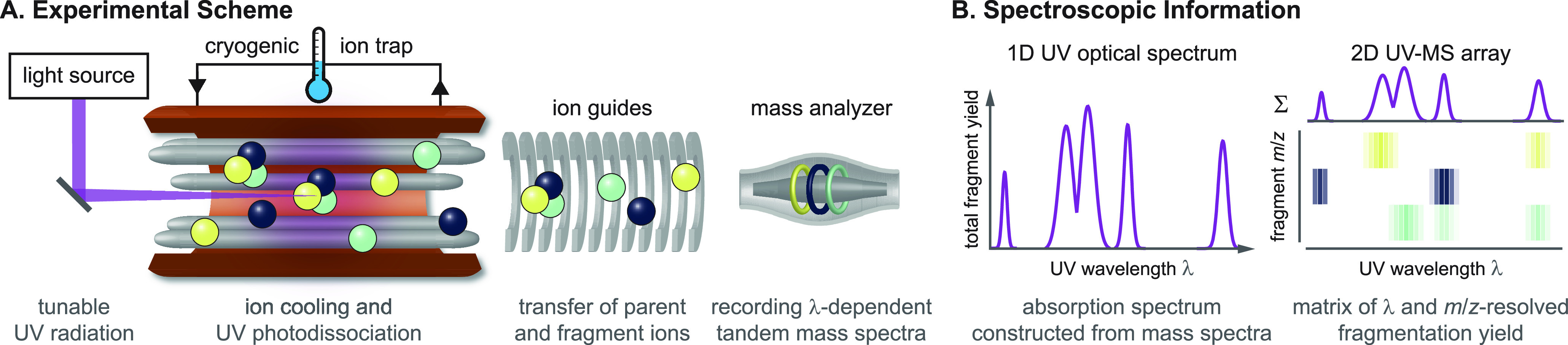
Schematic overview of cold-ion UV spectroscopy. (A) Simplified representation of an experimental scheme for recording UV spectra of cold ions. *m*/*z*-Selected parent ions, represented by three joint spheres, are cooled in a cryogenic ion trap and probed by UV radiation from a tunable laser, inducing photodissociation and/or photodetachment (latter not shown). The parent species and its photofragments (lone spheres) are extracted from the trap and transferred to a simultaneous mass analyzer, such as a ToF or FTMS device. (B) Plotting the overall fragmentation yield as a function of photon energy yields the UV optical spectrum of the parent ion. 2D UV-MS fingerprints correlate UV optical spectroscopic information and MS data. The fingerprints display entire fragment ion spectra as the function of excitation wavelength, condensing more analytical information into UV-MS matrices.

In contrast to slow-heating IRMPD, fragmentation patterns in UVPD may depend markedly on the irradiation wavelength. Dissociation pathways are affected by the initial site of excitation, i.e., the location of electrons involved in the electronic transition induced by photon absorption. This fundamental aspect of UVPD is utilized for analytical purposes in 2D UV-MS, an alternative way of handling and representing UV ion spectroscopic data.^208,209^ Instead of plotting the overall fragmentation yield as a function of photon energy, 2D UV-MS deals with 2D data arrays depicting entire fragment ion spectra as a function of irradiation wavelength. Figure 8 provides a graphical overview of gas-phase UV ion spectroscopy, its methodology, and the different ways of representing spectroscopic information.

Various biomolecular ions have been studied by UV action spectroscopy, including amino acids,^198^ peptides,^210−212^ and nucleic acids.^207^ Although still in its infancy, recording UV spectra of *m*/*z*-selected glycan ions has gained momentum in recent years. Glycans generally lack strong chromophores, which makes their UV spectroscopic analysis challenging. Species containing, for example, C=C double bonds or −COOH/–COO^–^ groups (with corresponding π → π* or n → π* transitions) may be analyzed directly,^213,214^ but many carbohydrates require alternative strategies. Boyarkin and co-workers introduced a method where glycans (analytes) were attached to protonated aromatics (chromophore reporters) through noncovalent interactions.^215^ The complexes formed spontaneously in the ESI process and allowed for the UV-spectroscopy-based distinction of carbohydrate isomers with weak or no inherent UV absorption.

Recently, UV ion spectroscopy has been performed using circularly polarized light, resulting in mass-resolved electronic circular dichroism spectroscopy of oligonucleotides (monitoring EPD)^216^ and amino acids (tracking UVPD).^217^ Although not yet applied for oligosaccharides, the method holds potential for MS-based glycan analysis in the future.

### 2.5 Theoretical Methods to Study Glycan Structure *in Vacuo*

Using gas-phase spectroscopy and IM-MS discussed above, detailed information may be obtained on the structure of isolated glycan ions, including the overall shape of the analytes, their functional groups, and the spatial orientation thereof. With computational chemistry, glycan structures can be modeled and then correlated with the experimentally obtained IR spectra or collision cross sections (CCSs). Subsequently, experimental CCSs and absorption bands can be matched with their computed counterparts, leading to an assignment of the structure to the probed glycan ion and their vibrations. The challenges lie in finding the correct structure by modeling the glycans’ conformational space and then extracting the computational IR spectra and CCSs out of that structure with the most efficient method.

The computational chemist’s toolbox usually comprises three different methods to tackle modeling of glycans: empirical force field and semiempirical and first-principles methods. Commonly, accuracy increases with computational expense that in turn is rising with the size of the system. Thus, the size of the system is the limiting factor for the accuracy, and each calculation is a trade-off between accuracy and computational expense.

A widespread approach to sample isolated carbohydrate ions is to employ empirical force fields.^218−220^ While promising force fields have been parametrized for glycans, they were commonly not developed for sampling isolated charged molecules in the gas phase and are therefore often limited to the analysis of neutral carbohydrates in the condensed phase.^35^ Contrary to first-principles methods, force field methods do not have their origin in quantum mechanics but in Newtonian physics. Therefore, force field methods are outperformed by first-principles methods that consider all electrons of the system or its electron density without an empirical bias.^221^ From all first-principles methods, mainly density functional theory (DFT) methods are used for carbohydrates. Promising results in terms of accuracy and reasonable computational expense have been obtained with hybrid DFT functionals^221^ such as PBE0^222,223^ or B3LYP,^224^ including dispersion correction^225,226^ and triple-ζ basis sets. Usage of basis sets from the Ahlrichs^227^ family is recommended, although accurate results can also be obtained with Pople^228^ or Dunning^229^ basis sets. However, the high accuracy of DFT comes at the cost of computational expense. Here, semiempirical methods that are based on DFT could fill the gap. Some integrals are replaced by empirical parameters leading to a significant increase in computation speed. Although some of these methods performed underwhelmingly for carbohydrates,^221^ several new methods are more promising.^230^

Each method can be used for sampling of glycans to generate a high number of structures, from which the most promising candidates are subsequently used for reoptimization at a higher level of theory (usually DFT). Commonly used methods for generating conformers from a reasonable starting structure are (replica exchange) molecular dynamics, genetic algorithms, or Monte Carlo simulations. It has been suggested that ideally the conformational search should start with DFT from the very beginning as the usage of a force field with a strong bias might lead to the exclusion of the most stable conformers, which cannot be recovered at a later stage of the process.^35^ As electrons are not considered in force field calculations, the initial connectivity of a molecule is rigid, and bond-breaking/-formation processes are not considered. It is a major limitation of methods relying on empirical force fields since rapid charge migration may occur in isolated carbohydrate ions. First-principles methods, in turn, can correctly determine structural features, such as ring puckering, glycosidic bond geometry, charge migration processes, or hydrogen bonding. For sampling, using a GGA-DFT functional with a small basis set is reasonable. The genetic algorithm FAFOOM^231^ can be interfaced with various software packages for DFT optimizations and yielded promising results for carbohydrate sampling.^55,56,221,232,233^ Other DFT-based approaches using molecular dynamics^234^ or Monte Carlo simulations^235−237^ have been implemented to sample carbohydrates. Tools based on semiempirical methods, such as CREST,^238^ were successfully used for modeling the conformational space of glycans.^239,240^

To get a reliable computed IR spectrum or CCS from a sampled structure, the structure needs to be reoptimized at a high DFT level of theory (such as PBE0+D3/def2-TZVP). The structure can subsequently be used to compute harmonic frequencies and Merz–Singh–Kollman (MK) charges. After scaling, harmonic frequencies often agree well with the experimental spectra. However, in various cases, certain absorption bands are strongly anharmonic. The issue can be circumvented by calculating anharmonic frequencies, albeit at an increased computational expense. The MK charges, on the other hand, are very reliable for calculating theoretical CCSs.

To get coordinates of a structure to theoretical CCSs, no quantum chemical calculation needs to be performed. Thus, computing CCSs is comparably fast, even for larger structures. Historically, CCSs were computed with the projection approximation^164^ (PA) or the exact hard-sphere scattering model^241,242^ (EHSS). For both methods, a mere structure is sufficient to compute the corresponding CCS. The PA differentiates only between hits and misses of the ion and the drift gas. In EHSS, also scattering is considered.^141^ The most reliable method is the trajectory method^243^ (TM) that has been implemented in the MobCal^179^ and hpccs^178^ packages. Here, partial charges ideally derived from a quantum chemical calculation are used for the calculation to predict accurate trajectories of the gas, leading to more reliable computed CCS values. The MK charges are recommended among all charge schemes.^179^

In summary, the available computational methods are fully capable of reliably calculating reasonable structures of isolated carbohydrate ions in the gas phase, and the presented workflow can also be applied to other classes of biomolecules, such as lipids^244^ or nucleotides.^245^ In most cases, it is recommended to use DFT methods not only for computing the energetics, spectra, and charges of the promising structural candidates but also for conformational sampling. Accuracy remains a question of computational expense, and for most mammalian glycans, not exceeding more than eight monosaccharide units, DFT approaches are feasible. In the long run, computational resources will increase, and sampling of larger carbohydrate ions at a higher accuracy will become possible. In the meantime, promising new semiempirical methods can fill the gap.

## 3 Human Milk Oligosaccharides

### 3.1 Structure and Analytical Challenges

The facts that breast-fed infants enjoy a variety of health benefits compared to their bottle-fed peers and that infant physiology is strongly influenced by intestinal bacteria have been known for over a century. After a connection between breast feeding and the composition of infant gut microbiota had been established, intense research led to the discovery of human milk oligosaccharides (HMOs) as the milk fraction responsible for promoting the growth of certain desired bacteria.^12^ HMOs are unconjugated glycans that represent the third largest fraction in colostrum and human milk (5–25 g L^–1^), following lactose and lipids.^12,246^ In contrast to lactose that serves as a caloric nutrient for infants, HMOs are digested and absorbed minimally, their functions being more diverse and complex.^247−249^ Besides helping to establish a healthy gut microbiota as prebiotics, HMOs also serve as antiadhesive antimicrobials. By resembling cell surface glycan epitopes, they act as soluble decoy receptors for enteric pathogens relying on glycan-mediated attachment to mucosal surfaces for infection. In addition, HMOs also perform regulatory roles, modulating intestinal epithelial cell and immune responses in infants.^250,251^

**Figure 9:**
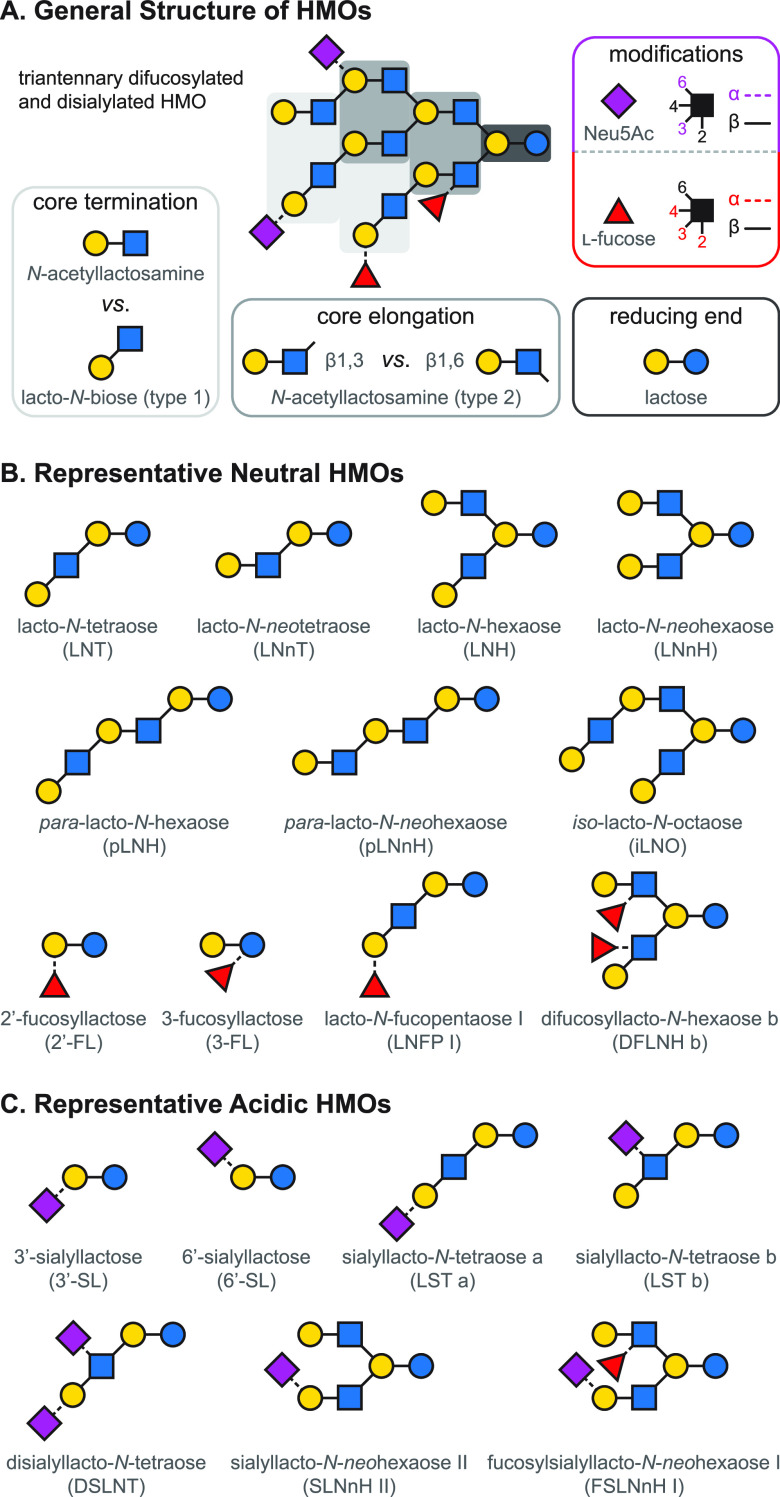
Overview of the structure of human milk oligosaccharides. (A) The five monosaccharide building blocks and basic structural blueprint of human milk oligosaccharides (HMOs), shown on the example of a hypothetical triantennary glycan. (B) Typical examples of neutral HMOs, highlighting both unmodified and fucosylated structures. (C) Representative acidic HMOs carrying one or more sialic acid residues.

Members of the HMO family can be derived from five monosaccharide building blocks: d-glucose (Glu), d-galactose (Gal), *N*-acetyl-d-glucosamine (GlcNAc), l-fucose (Fuc), and *N*-acetyl-d-neuraminic acid (Neu5Ac), the only sialic acid identified in human milk to date. The structure of HMOs follows a strict general blueprint, highlighted in Figure 9.^12,252−254^ During assembly of the core, a lactose (Lac) moiety—found at the reducing end of all HMOs—can be extended by *N*-acetyllactosamine (LacNAc, type 2) and lacto-*N*-biose (LNB, type 1) disaccharide units. While further disaccharide units may be attached to type 2 LacNAc residues, addition of a type 1 LNB unit terminates the respective antenna. Branching points in the core are introduced through β1,6-glycosidic linkages, leading to *iso*-HMOs with multiple antennae. The absence of such branching points results in *para*-HMOs with unbranched core structures, containing exclusively β1,3-linkages between the disaccharide units. Terminal and internal residues of the HMO core may be fucosylated via α1,2-, α1,3-, or α1,6-linkages. In addition, Neu5Ac residues may be attached to the core via α2,3- or α2,6-glycosidic bonds, leading to acidic oligosaccharides. The majority of HMOs is fucosylated, while 5–20% carry one or more sialic acids.^12,246^ As a result of these modifications, HMOs often display a diverse array of Lewis and blood group epitopes, frequently observed across various classes of glycans.

Despite the limited number of building blocks and the strict logic of their assembly, HMOs show remarkable structural diversity, with well over 100 different structures identified to date.^246,255^ Many of these oligosaccharides are isomers, differing only in their branching, fucosylation, or sialylation patterns. Although the anomeric configuration of Gal, GlcNAc, Fuc, and Neu5Ac residues is invariable in HMOs and determining the composition is straightforward owing to mass differences between the building blocks, elucidating branching and connectivity are major challenges in traditional MS-based HMO analysis. Thus, the present section focuses on recent developments in the field that facilitate isomer distinction and provide information on linkage positions, a crucial element of HMO structure.

### 3.2 Electron-Based Dissociation Methods in HMO Analysis

B-, C-, Y-, and Z-type fragment ions, resulting from glycosidic bond cleavages, carry essential information on the sequence and composition of oligosaccharides. A- and X-type cross-ring fragments, on the other hand, are indispensable for the assignment of linkage positions. As branching and connectivity are key aspects of HMO structure, considering both the core architecture and the attachment of modifications, dissociation methods capable of generating diverse and abundant cross-ring fragments have been at the center of MS-based HMO research. Low-energy CID and IRMPD are based on the gradual heating of ions and lead primarily to cleavage of the most labile bonds. This manifests in extensive glycosidic cleavages, while cross-ring fragments are generally scarce, especially in the case of protonated species.^256,257^ In addition, Fuc and Neu5Ac residues may be lost upon vibrational excitation of the analytes, impeding structural assignment.^23^ Through removing mobile protons that facilitate glycosidic bond cleavage, permethylation and adduction of metal ions may enhance the formation of cross-ring fragments in slow-heating methods.^258−260^ Despite these advances, information provided by CID and IRMPD on oligosaccharides is rather limited, inspiring scientists to explore gas-phase ion chemistry and alternative ion activation methods better suited for the specific needs of HMO research.

Electron-based or electron-mediated dissociation (ExD) methods, adopted from MS-based peptide and protein analysis,^79,81,261−265^ have proven to be extremely useful for the structural characterization of HMOs. Briefly, ExD methods are based on ion–electron interactions in the gas phase, inducing either rapid electronic excitation of analytes, the formation of charge-reduced radical ions, or both. Fragmentation pathways involving such radicals and excited electronic states lead to markedly different product ion spectra than those observed upon CID or IRMPD. In principle, ExD methods accompanied by charge reduction require multiply charged precursors: ECD and ETD are applied to polycations, while EDD and negative (N)ETD are suited for polyanionic species. In contrast, EED and EID do not involve electron transfer and are generally performed on singly charged precursors, irrespective of ion polarity.^88,266,267^ Although ETD and NETD utilize gas-phase ion–ion reactions instead of interactions between ions and cotrapped electrons, they are generally included into the broader family of ExD methods owing to similarities of the resulting fragmentation patterns and will be discussed as such herein.

The first ECD MS experiments on a milk oligosaccharide were performed by Adamson and Håkansson in 2007, employing an FTICR-MS platform and the unbranched *para*-lacto-*N*-hexaose (pLNH, see Figure 9) as the model compound.^85^ To facilitate the formation of doubly charged precursors and systematically investigate the influence of metal ion adduction on ECD, native pLNH was cationized with divalent alkaline earth and transition metal ions, such as Mg^2+^, Ca^2+^, Ba^2+^, Mn^2+^, Co^2+^, and Zn^2+^. In general, ECD provided complementary information to IRMPD, generating several cross-ring fragments not observed upon slow-heating photodissociation of pLNH dications. Subtle differences observed in the ECD patterns of the various pLNH adducts were attributed to the impact of metal ion coordination site on fragmentation and to that of the second ionization energy influencing the electron capture of analytes.

**Figure 10:**
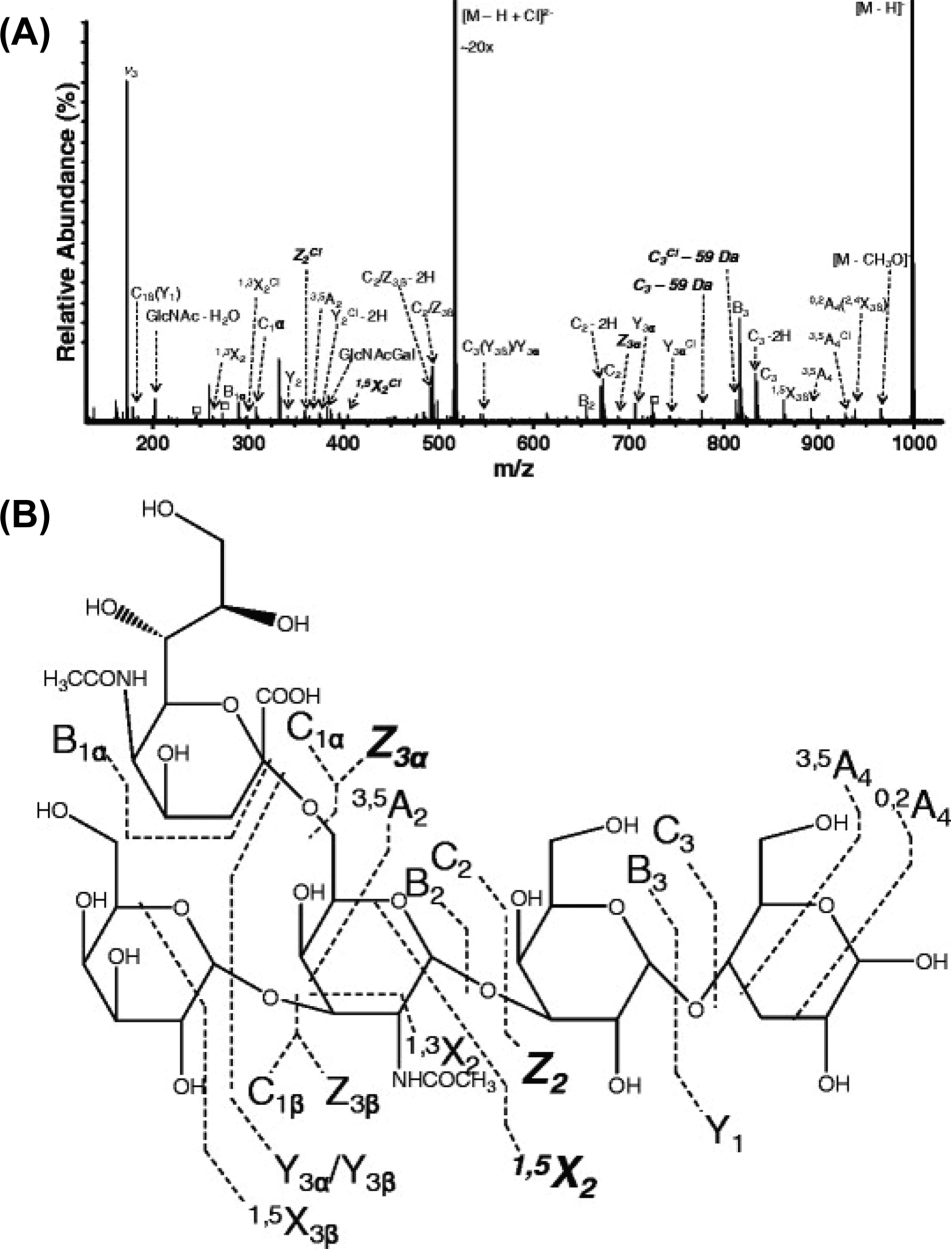
Electron detachment dissociation (EDD) of human milk oligosaccharide dianions. (A) EDD tandem mass spectrum of LST b as [M – H + Cl]^2–^, along with (B) the corresponding fragmentation pattern. Product ions depicted in bold appeared uniquely for the chloride-adducted species and were not observed upon EDD of the doubly deprotonated analogue. Fragments resulting from multiple cleavage sites are designated with a slash. Reprinted with permission from ref (269). Copyright 2012 American Society for Mass Spectrometry.

Following the introduction of ECD to the analysis of neutral HMOs in positive ion mode, the same authors employed its negative ion counterpart, EDD, for the structural characterization of sialylated structures.^268^ Disialyllacto-*N*-tetraose (DSLNT) and the sialyllacto-*N*-tetraose isomers LST a and LST b (see Figure 9) were studied as doubly deprotonated ions using FTICR-MS. Electron detachment, induced by electrons of moderate (20–30 eV) kinetic energy, led to charge-reduced anions that readily underwent radical-driven dissociation pathways, producing singly charged fragments. For all three model compounds, EDD led to a more diverse set of cross-ring fragments than CID or IRMPD of the respective [M – H]^−^ and [M – 2H]^2–^ species. In the case of LST b, as an example, ^0,4^A_2_ and ^3,5^A_2_ ions confirmed the 2,6-linkage of Neu5Ac, while the ^1,3^X_2_ fragment indicated 1,3-linkage of the terminal Gal residue, in accordance with the lacto-*N*-tetraose (LNT) core of the analyte. In a subsequent study employing the same underivatized model compounds, EDD of acidic HMOs was extended to their chloride adducts with the general formula [M – H + Cl]^2–^. In general, EDD spectra of chloride-adducted HMOs were dominated by singly charged product ions and contained a more diverse array of glycosidic and cross-ring fragments than that previously observed for doubly deprotonated species. While generating DSLNT chloride adducts by ESI proved to be challenging, LST isomers readily formed singly deprotonated chloride adducts with sufficient abundance. Figure 10 shows the EDD spectrum of [LST b – H + Cl]^2–^. Linkage positions of the Gal and Neu5Ac residues at the nonreducing termini may be unambiguously determined, owing to diagnostic fragments arising from cross-ring cleavages of the GlcNAc pyranoside.

**Figure 11:**
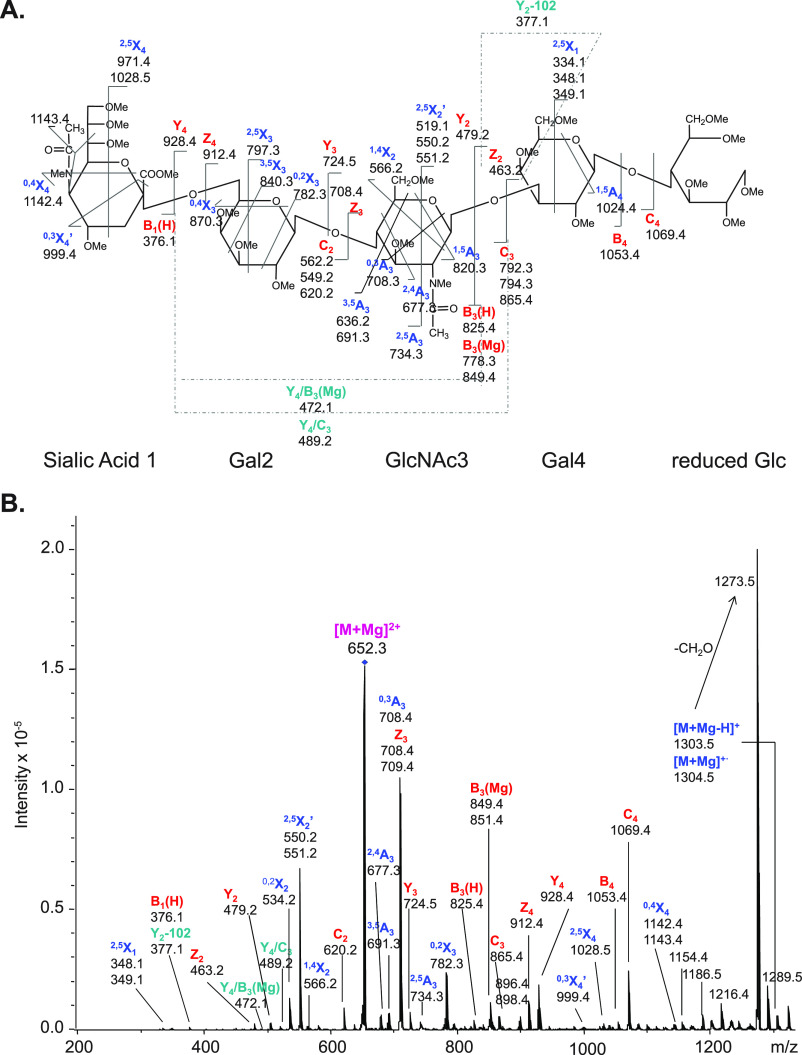
Electron transfer dissociation (ETD) of metal-ion-adducted human milk oligosaccharides. (A) ETD fragmentation pattern of reduced, permethylated LST c as [M + Mg]^2+^ (*m*/*z* 652.3). Note the diagnostic cross-ring fragments enabling the assignment of the LNnT core and the Neu5Ac linkage position. (B) Corresponding ETD tandem mass spectrum. Fragments between *m*/*z* 1142.2 and 1216.4 are attributed to cleavages within the sialic acid residue. Reprinted with permission from ref (257). Copyright 2011 American Society for Mass Spectrometry.

In 2011, Han and Costello employed ETD for the first time for the MS-based structural characterization of glycans.^257^ ETD is based on electron transfer from a suitable radical anion, generated usually from fluoranthene, to polycationic analytes. As ETD exploits ion–ion chemistry and does not require trapping electrons like most other ExD methods, it is readily compatible with linear and 3D quadrupole ion traps. The neutral tetrasaccharide LNT, its linkage isomer lacto-*N*-neotetraose (LNnT, see Figure 9), and three monosialylated LST isomers with either a LNT or LNnT core were studied as reduced, permethylated species. Systematic analysis of Na^+^, K^+^, Mg^2+^, and Ca^2+^ adducts revealed a strong dependence of ETD patterns and fragmentation efficiencies on the nature of the metal ion chosen. Alkali metal adducts of HMOs gave rise to a very limited number of fragments, proving unsuitable for ETD experiments. In contrast, Ca^2+^ and especially Mg^2+^ adducts yielded highly informative product ion spectra: numerous cross-ring fragments and extensive glycosidic cleavages enabled distinction of isomeric HMOs and assignment of the underlying structures. Figure 11 shows the ETD spectrum of reduced, permethylated LST c as a Mg^2+^ adduct, along with the assignment of product ions, among which both odd- and even-electron species are present. The 2,6-linkage of Neu5Ac in LST c could be confirmed based on ^3,5^X_3_ and ^0,4^X_3_ fragments, while the ions ^3,5^A_3_, ^0,3^A_3_, and ^2,4^A_3_ indicated a 1,4-linkage between the Gal and GlcNAc residues, verifying the LNnT core of the monosialylated analyte. In cases when MS^2^ with ETD did not allow for an unambiguous structural assignment, the authors utilized the MS^*n*^ capabilities of the quadrupole ion trap instrument to combine ETD and CID in a sequential manner. While the linkage position of the nonreducing end Gal in LNT and LNnT could not be determined by single stage ETD, subsequent CID of a singly charged internal ETD fragment provided the information required to decipher connectivity in each isomer, demonstrating the potential of MS^*n*^ strategies to combine complementary ion activation methods.

The latest ExD method introduced to MS-based HMO analysis is EED. EID and EED refer to closely related processes and are sometimes used interchangeably in the literature, with the former acronym applied as a more general term. To avoid confusion, we always use the term favored by the authors in the respective studies. Unlike the techniques discussed so far in the present section, EED is not accompanied by charge reduction and may be readily performed on singly charged species. Taking advantage of this feature and building on the work of Gao et al.,^270,271^ Lin and co-workers performed EDD on five LNFP isomers derivatized with the *N*-methylated form of a sequestered proton reagent for acid-catalyzed glycan sequencing (Me-PRAGS).^272^ Me-PRAGS is a quaternary ammonium compound with a fixed positive charge that can be readily attached to the reducing end of glycans. EDD of Me-PRAGS-derivatized LNFP cations in an FTICR cell generated primarily fragments retaining the charge at the reducing end. Upon irradiating the analytes with 12 eV electrons, complete sets of sequence-informative ^1,5^X-, Y-, and Z-type ions were observed. Employing electrons with 16 eV kinetic energy led to more efficient fragmentation, to the formation of doubly charged product ions, as well as to a higher number of linkage-informative cross-ring and secondary fragments. The study reveals the capabilities of EED MS to sequence neutral, unmethylated glycans following charge tagging and to distinguish isomers based on differences in their product ion spectra. We hope the selected works above not only reflect the utility of ExD methods in HMO analysis but also demonstrate the importance of basic research to develop techniques that provide information previously unattainable by conventional MS-based methods.

### 3.3 Ultraviolet Photodissociation Mass Spectrometry of HMOs

Another ion activation method that has been successfully employed to gain information on branching and connectivity in HMOs is UVPD. The rapid increase of internal energy, electronic excitation, and occasional EPD accompanying the absorption of energetic UV photons open up diverse fragmentation pathways in glycan ions, manifesting in abundant cross-ring and internal fragments.^32^ Unlike charge-reducing ExD methods, such as (N)ETD or ECD/EDD, photodissociation may be readily performed on singly charged species. In addition, UVPD is compatible with a variety of MS platforms, provided suitable optical access is established for a sufficiently bright UV source, most commonly an excimer or solid-state Nd:YAG laser.

In general, native HMOs lack strong UV chromophores, exhibiting relatively low absorption cross sections at lower UV photon energies. Thus, the first UVPD MS study on HMOs was performed using 157 nm vacuum ultraviolet (VUV) radiation, produced by a nanosecond F_2_ excimer laser.^273^ Following these initial experiments on native and reducing end-modified oligosaccharides, Reilly and co-workers systematically studied the VUV photofragmentation of acidic HMOs as positive ions in a linear ion trap.^274^ The analytes—permethylated to avoid the loss of labile Neu5Ac residues—showed extensive fragmentation upon interaction with 7.9 eV VUV photons, resulting in numerous cross-ring fragments. Isomeric 3′- and 6′-sialyllactose (3′- and 6′-SL, see Figure 9) could be unambiguously distinguished based on their markedly different product ion spectra. In addition to mere distinction, the sialic acid linkage position could be confidently assigned in both species, owing to diagnostic cross-ring fragments whose formation involves the cleavage of the C3–C4 or C4–C5 bond in the Gal residues. Following the assignment of the sialic acid linkage position in the simplest acidic HMOs, the authors demonstrated the capabilities of 157 nm UVPD MS for the structural characterization of larger sialylated species. In the monosialylated biantennary lacto-*N*-neohexaose SLNnH II (see Figure 9), the combination of ^0,4^A_4_, ^1,5^X_3β_, ^1,5^X_3β_, and ^2,4^A_4_/^1,5^X_3α_ fragments confirmed the location of Neu5Ac being on the β3 antenna of the heptasaccharide.

**Figure 12:**
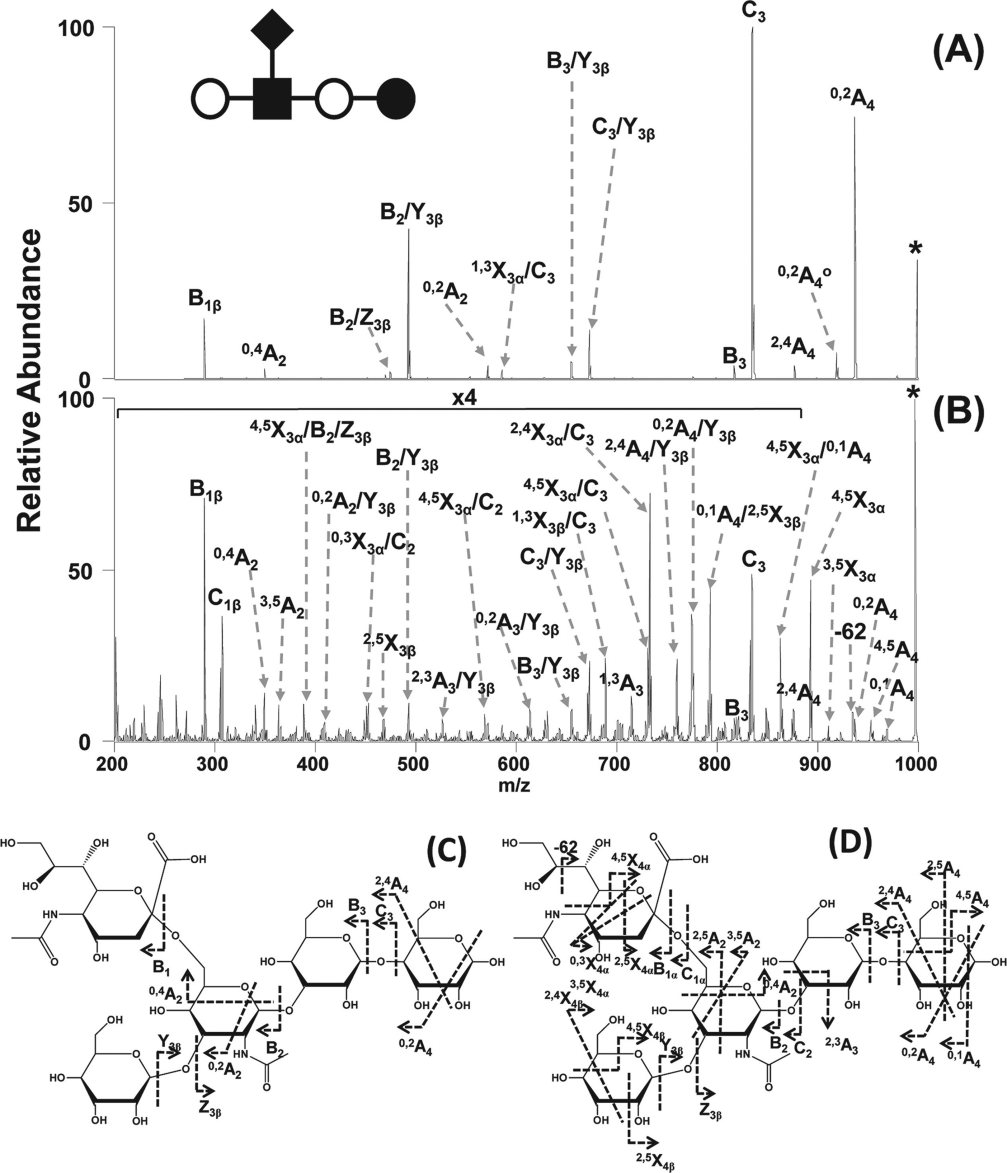
193 nm ultraviolet photodissociation (UVPD) of deprotonated human milk oligosaccharides. (A) Product ion spectra of singly deprotonated LST b (*m*/*z* 997) employing (A) collision-induced dissociation (CID) and (B) 193 nm UVPD. The corresponding (C) CID and (D) UVPD fragmentation patterns and assignments. The precursor ion is labeled with an asterisk. Reproduced with permission from ref (76). Copyright 2011 American Chemical Society.

Ko and Brodbelt analyzed acidic HMOs (dp3–dp7, dp being the degree of polymerization) in their native form by UVPD MS in negative ion mode.^76^ Deprotonated ions were irradiated in a linear ion trap using a 193 nm (6.4 eV photon energy) ArF excimer laser, and the resulting fragmentation patterns were compared to those obtained by CID on the same MS instrument. The ArF laser with 8 mJ pulse energy provided efficient photofragmentation, allowing for short UVPD interaction periods on the low millisecond time scale. In general, UVPD provided not only better sequence coverage than CID but also more extensive cross-ring cleavages, as highlighted in Figure 12, on the example of LST b. Abundant A- and X-type photofragments carry information on connectivity, e.g., on the linkage position of the terminal Gal in LST b, that could not be assigned based on CID experiments. Interestingly, UVPD of dianions resulted mainly in singly charged fragments, while CID mass spectra were rich in doubly charged product ions. This phenomenon could be attributed to structural differences between photo- and CID fragments, retaining in general one vs two sialic acids, respectively. Charge-reduced EPD products among the photofragments of multiply charged anions were of relatively low abundance.

An inventive approach to make HMOs more suitable for UVPD MS at longer wavelengths utilizes covalent attachment of aromatic chromophores to the glycans. In a proof-of-concept study, four isomeric lacto-*N*-fucopentaoses (LNFP I, II, III, and V) and three lacto-*N*-difucohexaose isomers (LNDFH Ia, Ib, and II) were derivatized with various fluorophores via reductive amination.^275^ The labels, such as 6-aminoquinoline (6-AQ) and 7-aminomethylcoumarin (AMC), are commonly applied in LC-MS glycomics workflows to facilitate fluorescent detection. Here, the tags served to increase the absorption cross section of the analytes at 355 nm (3.5 eV), corresponding to the third harmonic of a Nd:YAG laser. While CID of singly sodiated species generated abundant Y-type ions retaining the fluorophore at the reducing end, photodissociation at 355 nm led mainly to A- and C-type fragments. In general, photodissociation pathways were largely unaffected by the nature of the fluorophore, whereas UVPD efficiencies appeared to be strongly influenced by the label, with 6-AQ providing the most efficient fragmentation. Although UVPD in general provided superior isomer differentiation compared to CID, LNFP II and III—both carrying a Fuc on their subterminal GlcNAc residue, only at different linkage positions—could not be distinguished based on their photofragments. In addition to direct UVPD of singly sodiated species, the authors performed electron photodetachment dissociation—also termed activated (a-)EPD—on LNDFH II anions. HMOs derivatized with 7-amino-1,3-naphthalenedisulfonic acid (AGA), a fluorophore with high solution- and gas-phase acidity, readily form multiply deprotonated species when electrosprayed in negative ion mode. Upon photon absorption at 355 nm, part of the [(LNDFH II + AGA) – 2H]^2–^ ion population underwent EPD, resulting in charge-reduced radical anions. CID of the singly charged radical species and of its even-electron analogue led to remarkably different fragmentation patterns, the former displaying a more diverse array of product ions. Thus, a-EPD has the potential to complement more widespread ion activation techniques and become a powerful hybrid dissociation method in MS-based glycan analysis.^276^

**Figure 13:**
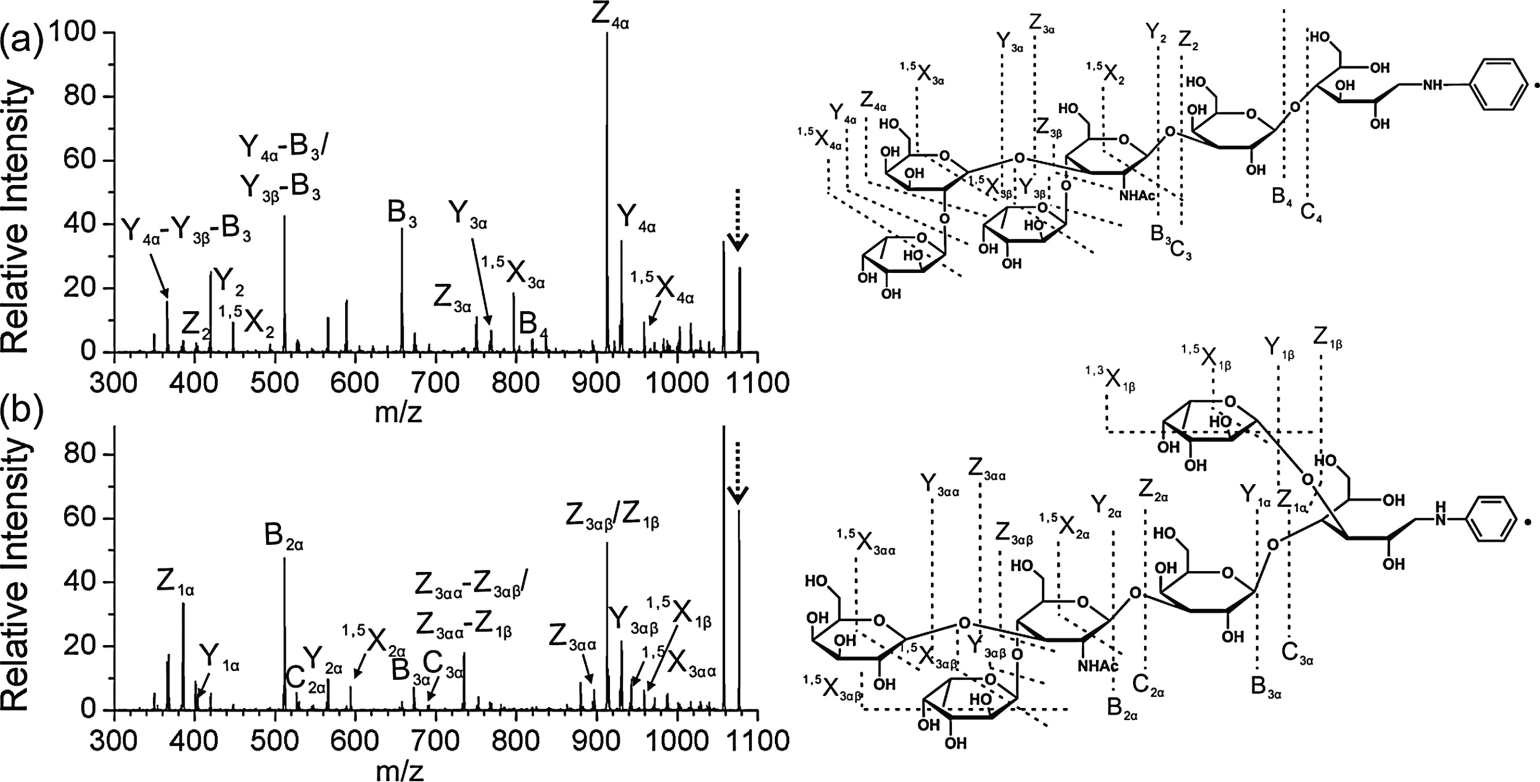
Radical-directed dissociation (RDD) of protonated human milk oligosaccharides at 266 nm. (a) RDD tandem mass spectrum and product ion assignment of singly protonated LNDFH I labeled with 4-iodoaniline. (b) RDD of the singly protonated LNDFH II isomer labeled with the same radical precursor. Reprinted with permission from ref (277). Copyright 2014 Elsevier B.V.

In the Julian lab, an alternative strategy was developed to enable efficient UVPD MS analysis of HMOs at lower photon energies.^277^ The method utilizes radical chemistry and relies on labeling glycans with the aromatic radical precursor 2-iodoaniline at their reducing end. Homolytic cleavage of the carbon–iodine bond is induced upon absorption of 4.7 eV photons, provided by a Nd:YAG laser (266 nm). The photocleavage generates hydrogen-deficient oligosaccharide radical ions, which readily undergo radical migration and dissociation upon collisional activation. This radical-directed dissociation (RDD) approach enabled unambiguous distinction of two pairs of fucosylated HMO isomers. The extensive set of glycosidic and cross-ring fragments generated from singly protonated parent ions by RDD is shown in Figure 13, on the example of LNDFH I and II. Although the most abundant, ^1,5^X-type cross-ring fragments do not reveal the underlying branching and connectivity, several diagnostic signals can be observed that appear uniquely for only one species, demonstrating the utility of RDD for the distinction of isomeric HMOs.

### 3.4 Ion Mobility–Mass Spectrometry in HMO Analysis

With the advent of commercial IM-MS instruments, the separation of isomeric HMOs based on their gas-phase mobilities has gained significant momentum. In general, the CCS and mobility of molecular ions strongly depend on the three-dimensional structures they adopt in the gas phase. As differences in composition, configuration, connectivity, or branching often manifest in different size and shape, isomeric glycans may be efficiently distinguished, separated, identified, and quantified by IM-MS.^128,129^

In a comparative study, Williams et al. employed a custom-built DTIM-MS and a commercial TWIM-MS device to study pairs of LNFP and LNDFH isomers as singly charged cations.^278^ Each HMO Na^+^ adduct displayed a sole, symmetric arrival time distribution (ATD) on both IM-MS platforms. This observation was in accordance with molecular modeling calculations, revealing a single family of low-energy conformers for all four sodiated glycans ions. Experimental ^DT^CCS_He_ values were generally in good agreement with those obtained by TM calculations, further verifying the low-energy structural candidates. The rather small difference in the mobilities of isomeric HMOs, however, limited the efficiency of separations, which underlines the importance of resolving power and selectivity in IMS experiments.

In comparison to chromatography or condensed-phase electrophoretic techniques, the possibilities to improve selectivity in IMS through altering the separation medium are rather restricted. Although changing the buffer gas composition may lead to better separation in certain cases, the effects are not comparable to those resulting from tuning a myriad of experimental parameters over a broad range, as possible in HPLC. Therefore, various strategies were developed in IMS to influence the conformation of analytes, with the aim of increasing the relative CCS difference (ΔCCS/CCS_avg_) of isomers and ultimately improving the resolution between critical peaks.

Fenn and McLean applied an in-house developed DTIM-MS instrument to study an extensive set of carbohydrate standards and recorded over 300 ^DT^CCS_He_ values for intact and fragment ions generated though in-source dissociation.^132^ Among the 31 model compounds, four LNFP and two LNDFH isomers represented HMOs. In accordance with previous works on smaller carbohydrates,^131^ metal ions had a significant impact on the measured mobilities and CCSs of larger glycans. These findings highlight the role metal ions play in determining the gas-phase structure of HMOs, an effect successfully harnessed in various studies to improve isomer separations by IMS.

**Figure 14:**
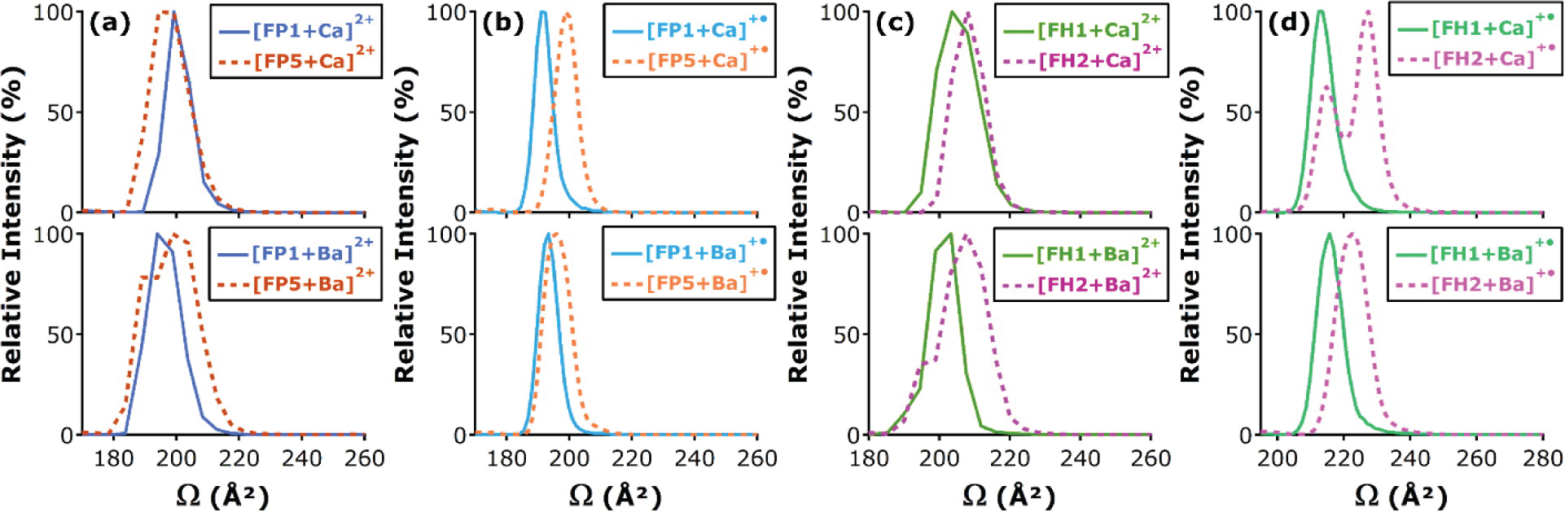
Nondissociative electron transfer (ETnoD) combined with traveling wave ion mobility (TWIM) separations for the analysis of fucosylated milk oligosaccharides. (a) Collision cross section (CCS) distributions of LNFP I and V isomers as calcium and barium ion adducts, along with (b) the CCS distributions of the respective singly charged radical ETnoD products. (c) CCS distributions of LNDFH I and II isomers as calcium and barium ion adducts and (d) that of the respective ETnoD products. Reproduced with permission from ref (280). Copyright 2015 American Chemical Society.

In a series of closely related experiments, Huang and Dodds investigated group I–II metal ion adducts of fucosylated HMOs, employing a commercial TWIM-MS device with N_2_ as the drift gas. First, two LNFP and two LNDFH isomers were analyzed as singly charged cations formed with Li^+^, Na^+^, K^+^, Rb^+^, and Cs^+^ ions.^279^ To convert the measured arrival times to ^TW^CCS_N2→He_ values, protonated polyalanine ions with known ^DT^CCS_He_ were used as calibrants (the notation to designate the buffer gas and IMS technique of choice as super- and subscript follows widely accepted recommendations^138^). Despite the buffer gas mismatch, the ^TW^CCS_N2→He_ values of the alkali-metal-adducted glycans formed a consistent set. CCSs of LNDFH I and II metal adducts varied in parallel, first increasing with increasing metal ion radius and then stagnating at around 224 and 220 Å^2^, respectively. In stark contrast, the CCSs of LNFP V ions increased steadily with increasing metal ion size, while the measured CCS values of LNFP I adducts decreased in the following order: [M + Cs]^+^ > [M + Na]^+^ > [M + K]^+^ > [M + Rb]^+^ > [M + Li]^+^. Owing to the remarkably large CCS of [LNFP I + Na]^+^, the best separation for LNFP isomers was achieved using Na^+^ adduction. The results clearly demonstrate the possibility to improve selectivity in IM separations of HMOs through the careful choice of adduct-forming metal ions. Building on these findings, the authors combined gas-phase nondissociative electron transfer (ETnoD) with subsequent TWIM separation of Ca^2+^- and Ba^2+^-adducted HMOs.^280^ Employing the same four model glycans as previously and the 1,4-dicyanobenzene radical anion as the electron donor, the separation of isomers with and without prior electron transfer to the analytes was compared. Figure 14 shows that charge-reduced [M + Ca]^+•^ radical ETnoD products show better separation than their even-electron counterparts. In contrast, no significant improvement could be achieved for Ba^2+^ adducts with this strategy, due to comparable shifts in the CCS of the isomers upon ETnoD. Although the aforementioned electron-transfer-induced CCS shifts are rather unpredictable, they appear to be both isomer-specific and metal-ion-dependent, revealing the clear analytical potential of the method. The ETnoD-TWIM-MS approach was successfully extended to additional fucosylated compounds, in combination with the full set of nonradioactive alkaline earth metals.^281^ Complete rationalization of these experimental observations would require high-level theory. Although the sheer size and complexity of the analytes make such attempts extremely challenging, state-of-the-art theoretical methods may reveal the underlying atomic level structure of oligosaccharide metal ion adducts in the foreseeable future.

The utility of metal ion adduction to improve HMO isomer separations was further demonstrated by Baker and co-workers, employing DTIM-MS with N_2_ as a buffer gas.^282^ While singly sodiated LNT and LNnT were inseparable due to nearly identical mobilities, K^+^ and singly deprotonated Zn^2+^ adducts of the same ions showed increasingly better separation, enabling their distinction. Interestingly, [LNT + 2Na]^2+^ and [LNnT + 2Na]^2+^ were baseline resolved, with the isomers exhibiting reverse migration order compared to K^+^ and Zn^2+^ adducts. Another important finding of the study concerns the effect of ion polarity. While small glycan isomers displayed in general better separation as Na^+^ adducts, dp4–dp6 oligosaccharide isomers exhibited larger differences in their mobilities when analyzed as deprotonated species. As an example, lacto-*N*-hexaose (LNH) and lacto-*N*-neohexaose (LNnH, see Figure 9) showed only partial separation as sodiated ions, but the same compounds could be baseline resolved when analyzed as singly deprotonated species, in accordance with previous findings.^283^

**Figure 15:**
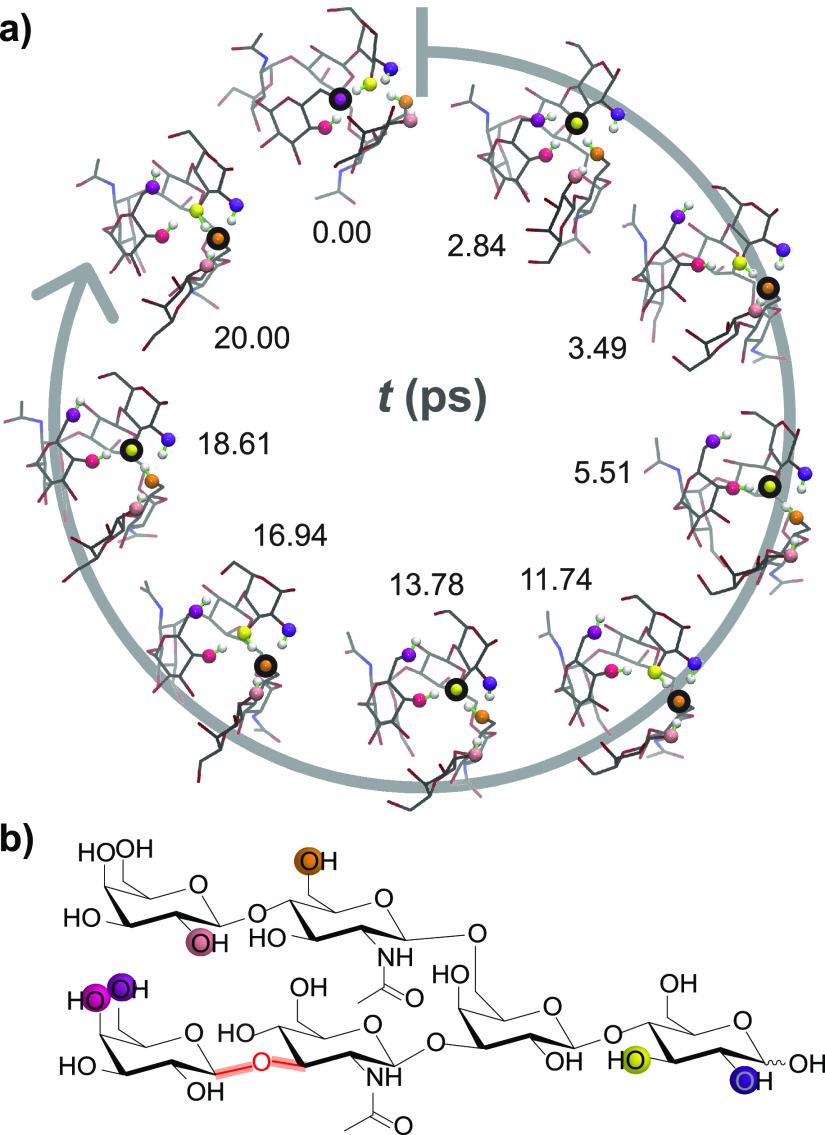
*Ab initio* molecular dynamics reveals rapid charge migration in a deprotonated milk oligosaccharide. (a) Snapshots of singly deprotonated LNH at different simulation times. Colored spheres indicate the OH groups involved in charge migration. The position of the charge within each structure is highlighted by a black circle. (b) Chemical structure of LNH; the colored spheres correspond to the OH groups in the upper panel that are deprotonated over the 20 ps simulation. Reprinted from ref (283). Published by The Royal Society of Chemistry. Copyright 2016 Struwe et al. (Creative Commons Attribution 3.0 Unported License).

Inspired by the efficient IM separation of deprotonated carbohydrate isomers,^133^ Struwe et al. studied the influence of ion polarity and adduct formation on the gas-phase structure of glycans, combining TWIM-MS experiments with DFT and *ab initio* molecular dynamics (MD).^283^ LNH and LNnH showed significantly improved separation in N_2_ as deprotonated species compared to their chlorinated, sodiated, and protonated counterparts. To rationalize the observations, experimental ^TW^CCS_N2→He_ values were compared to those calculated for DFT-optimized candidates. In general, experiment and theory showed a good match for sodiated ions; [LNT + Na]^+^ and [LNnT + Na]^+^ adopt similar, compact gas-phase structures, governed mainly by charge solvation. Interestingly, ^TW^CCS_N2→He_ of the respective deprotonated ions matched those calculated for neutral species. The *ab initio* MD simulations performed on exemplary conformations of [LNH – H]^−^ revealed rapid charge migration on the picosecond time scale, blurring the negative charge over the molecule during the millisecond-long IM separation (Figure 15). This charge delocalization provides a rationale for the above-mentioned unusual agreement: the experimental CCS of the deprotonated species represents an average of several, rapidly interconverting deprotonation site isomers, thereby approaching the CCS of the respective neutral species.

Although selectivity is a key aspect of IM separations, resolving power is equally important in determining the extent to which two peaks are separated. To demonstrate the role of resolving power, Pu et al. combined TIMS with EED on an FTICR-MS platform to separate and identify the linkage isomers LNT and LNnT as permethylated, singly sodiated ions.^284^ The method, termed selected accumulation (SA-)TIMS,^285^ enables the hyphenation of millisecond-long IM separations to slow mass analyzers and ExD techniques requiring comparably long interaction periods. Briefly, mobility-selected ions were accumulated on the electric field plateau of the TIMS tunnel, in front of a small potential barrier near the exit funnel. After reaching a sufficiently large population of mobility-selected ions, the barrier may be lowered to extract ions for subsequent fragmentation and mass analysis. The tetrasaccharides were baseline resolved by TIMS, and the linkage position of the terminal Gal could be determined in each isomer owing to diagnostic cross-ring fragments previously not observed in CID, ECD, or ETD spectra. Thus, TIMS-EED-MS/MS enabled both rapid distinction and structural assignment of HMO linkage isomers. ^TIMS^CCS_N2_ values obtained through calibration agreed with those determined by a commercial DTIM-MS device, providing further structural information on the analytes.

**Figure 16:**
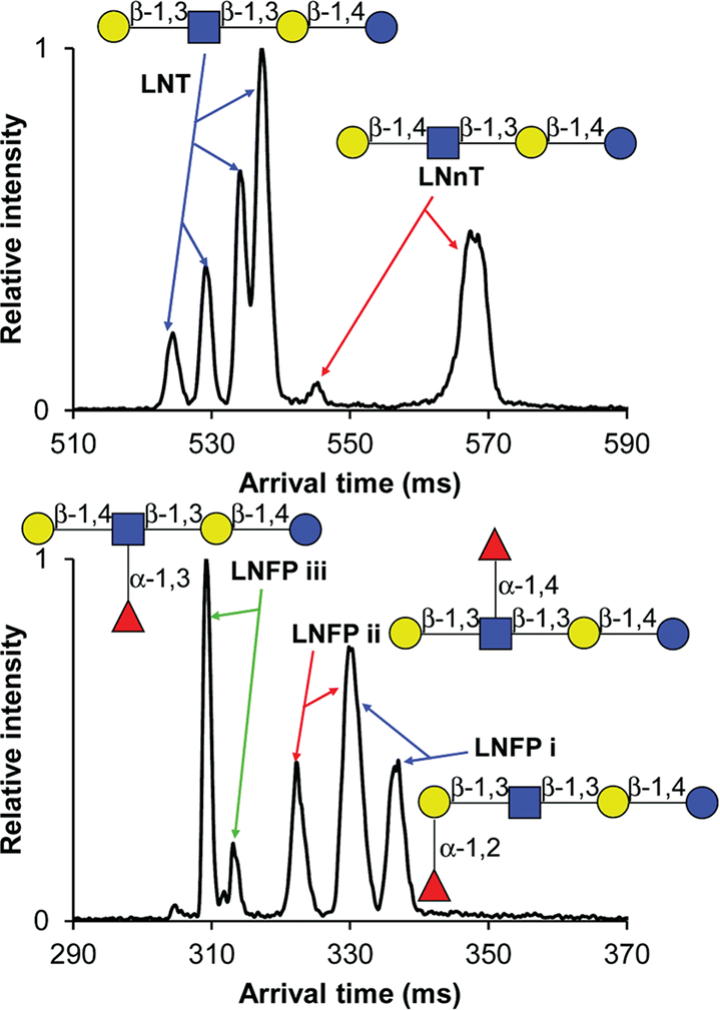
Ion mobility separation of human milk oligosaccharides in structures for lossless ion manipulations. The upper panel shows the arrival time distributions (ATDs) of singly protonated LNT and LNnT, resulting from a 31.5 m separation. The lower panel depicts the ATDs of three LNFP isomers as the outcome of a 45 m separation. LNFP isomers were measured as doubly charged [M + H + K]^2+^ species. Reprinted with permission from ref (134). Copyright 2018 The Royal Society of Chemistry.

Another platform that enabled a significant increase in the resolving power of IM separations is termed structures for lossless ion manipulations (SLIMs), developed by Smith and co-workers.^286−289^ SLIM technology utilizes printed circuit boards (PCBs) to pattern electrodes on planar surfaces, and ion manipulation is achieved in a suitable buffer gas by a combination of RF and DC fields. SLIM-based devices have been successfully applied for TWIM separations at low pressures, offering the possibility to create extremely long separation pathways through serpentine routes and multipass devices. Employing a 13 m long serpentine SLIM module with N_2_ buffer gas, singly sodiated LNFP I and II ions could be fully resolved within 550 ms.^290^ In a SLIM device enabling multiple passes through its 13.5 m long separation route, [LNH + H + K]^2+^ and [LNnH + H + K]^2+^ were baseline resolved already after a single pass.^152^ After accomplishing nine rounds and thereby covering a total of 121.5 m, an additional feature appeared in the ATD of [LNnH + H + K]^2+^. This feature had remained unnoticed in previous IM-MS experiments but appeared here as a distinct peak owing to the extremely high resolving power provided by the multipass SLIM technology. Building on these intriguing findings, the authors set out to unravel hidden features in the ATDs of various glycan ions.^134^ Upon high-resolution SLIM-based TWIM-MS analysis, four distinct peaks could be observed in the ATD of singly protonated LNT and two in that of its isomer LNnT. Similarly, several well-resolved features appeared in the ATD of LNFP I, II, and III ions, analyzed as [M + H + K]^2+^. These features, highlighted in Figure 16 may correspond to α/β-anomers, protomers, or conformers that do not interconvert on the subsecond time scale of the separations. Gaining more information on the nature of these species would require the hyphenation of SLIM-based IM-MS to orthogonal analytical methods, such as IR ion spectroscopy, which is addressed in Section 3.5.

At last, an interesting study by Li and co-workers—dealing with the integration of IMS into a multidimensional glycomics workflow—needs to be mentioned.^291^ The authors coupled capillary zone electrophoresis (CZE) to TWIM-MS and successfully analyzed three LST isomers labeled with a carbonyl-reactive aminoxy tandem mass tag (aminoxyTMT) for quantitation. Although LST b and LST c comigrated in CZE, they could be readily separated by TWIMS as [M + H + Na]^2+^ ions. The orthogonality observed between the two electrophoretic techniques demonstrates that “size-to-charge ratios” of glycans are governed by markedly different effects in solution and in the gas phase.

An important aspect of MS-based glycan analysis is fucose migration, which also affects the structural characterization of HMOs. Owing to their importance and relevance to a variety of glycan classes, rearrangement reactions of gas-phase oligosaccharide ions will be addressed separately in Section 5.6.

### 3.5 HMO Analysis by Gas-Phase Ion Spectroscopy

**Figure 17:**
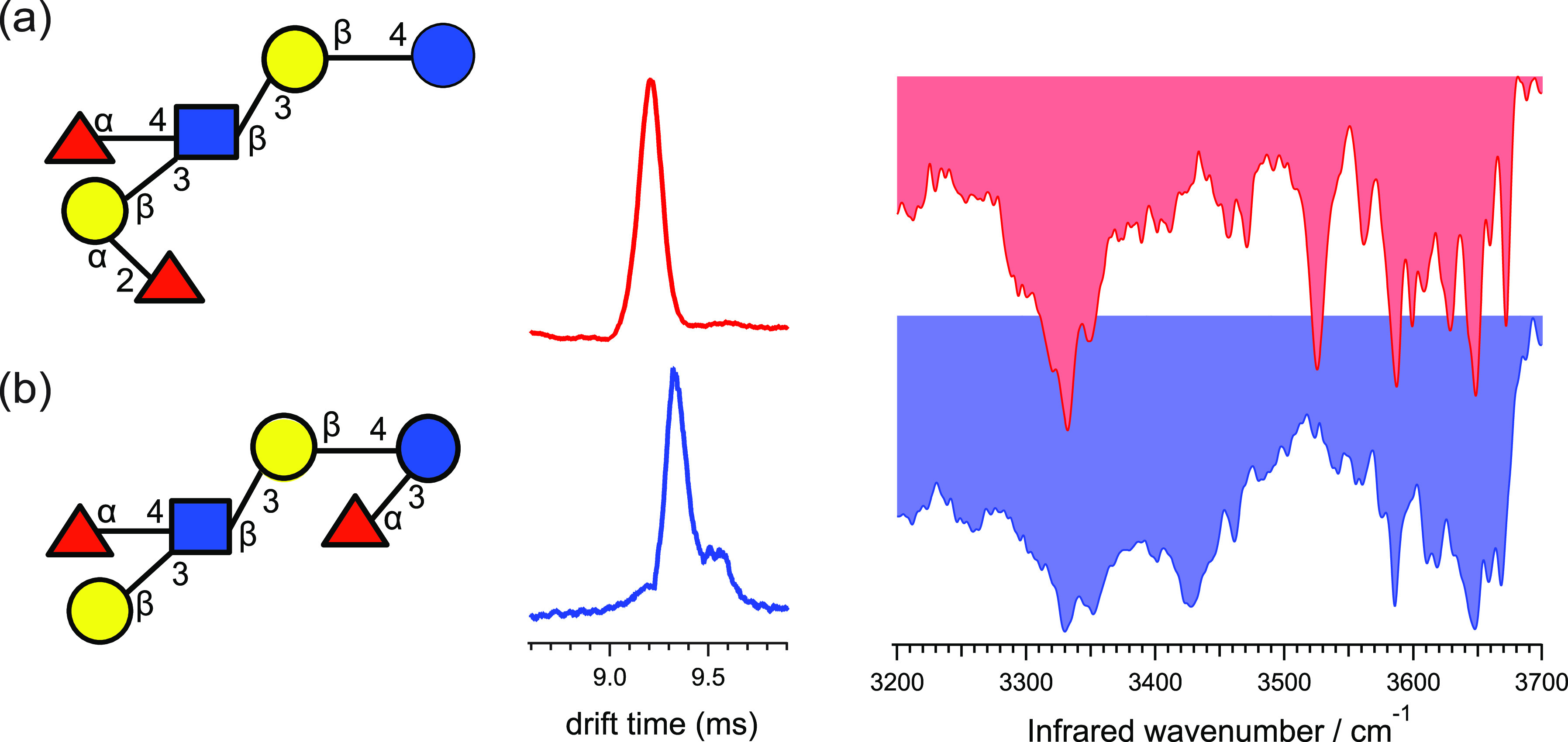
Gas-phase IR spectroscopy of human milk oligosaccharides. Arrival time distributions and vibrational spectra of sodiated (a) LNDFH I and (b) LNDFH II, each tagged with one molecule of N_2_. The drift times and peak widths are (a) 9.21 ms, fwhm = 0.13 ms; and (b) 9.32 ms, fwhm = 0.13 ms. Reprinted with permission from ref (292). Copyright 2018 The Royal Society of Chemistry.

Gas-phase ion spectroscopy in the UV and IR region for the characterization of HMOs has been the subject of recent research and shows great potential.^33,36,208^ In a more general approach across different glycan classes, the first IR spectrum using messenger-tagging spectroscopy in the OH-stretching region (here 3200 to 3700 cm^–1^) of *N*-acteyllactosamine, a core termination in HMOs, as a sodium adduct was published.^189^ The IR spectrum is well-resolved and distinguishable from the IR spectra of five isomeric disaccharides. The approach was challenged with the identification of HMOs up to hexasaccharides which yielded highly resolved IR spectra that are shown in Figure 17.^292^ A high-resolution IMS stage prior to messenger-tagging serves as additional separation in the case where multiple conformers and anomers are present.^201,293^

Conformer-selective IR spectroscopy can, furthermore, be performed in IR-IR double resonance spectroscopy in which the first pump laser is fixed to a certain wavelength which is specific to one conformer only, and the second probe laser is scanned through the wavenumber range. For *N*-acetyllactosamine, the IR signature is identical to the IR signature using high-resolution IMS prior to IR spectroscopy,^294^ yet both approaches require advanced instrumental setups. Recently, the first cryogenic UV spectra of HMOs were recorded as 2D UV-MS spectra which is an alternative way of representing the spectroscopic data in 2D data arrays of fragment ions, as described in Section 2.4.^295^ The lack of a chromophore in HMOs is circumvented by mixing the glycans in solution with aromatic molecules which ionize as noncovalent protonated complexes. Gas-phase ion spectroscopy in combination with rich databases is capable of identifying glycans in cases in which LC, tandem MS, and IMS reach their limits.

## 4 *N*-Linked Glycans

### 4.1 Structure and Analytical Challenges

Glycosylation is the most important post-translational modification of proteins.^26^*N*-Glycans are branched oligosaccharides that are bound, most commonly, via GlcNAc to an Asn residue of the protein backbone. Protein glycosylation generally occurs only to Asn-Xxx-Ser/Thr sequons, where Xxx can be any amino acid except for proline.^26,296^ In rare cases, the third amino acid of the sequon can also be cysteine.^297^*N*-Glycans can be found in all living beings and viruses and exhibit various important physiological roles. *N*-Glycoslyation influences several properties of glycoproteins, such as, for example, “their conformation, solubility, antigenicity, activity, and recognition by glycan-binding proteins”.^298^ Furthermore, several human diseases, such as arthritis, can be linked to *N*-glycosylation.^299,300^

**Figure 18:**
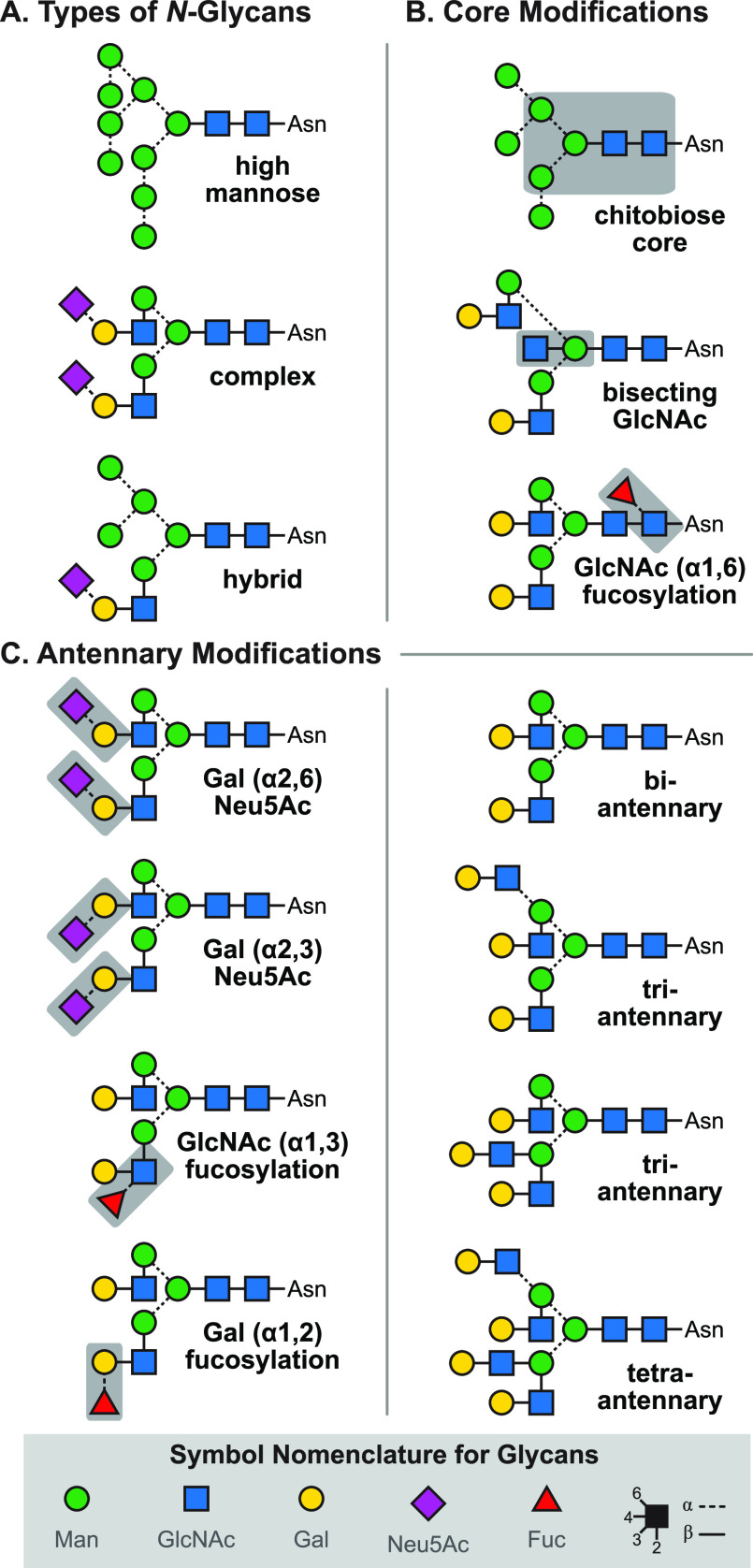
Most common types and modifications of *N*-glycans found in mammals. (A) Exemplary structures for three types of *N*-glycans: high-mannose (sometimes called oligomannose), complex, and hybrid. (B) Common core modifications of mammalian *N*-glycans and chitobiose core, a subunit common to all *N*-glycans. (C) Common antennary modifications.

Here, we will focus on *N*-glycans that can be found on mammalian proteins. Commonly, *N*-glycans are enzymatically cleaved from proteins or peptides using peptide *N*-glycosidases (PNGase) prior to analysis. It is also possible to directly analyze glycopeptides, which will be discussed later. A common motif of all *N*-glycans is the chitobiose core (Figure 18B), composed of three mannose and two GlcNAc moieties, which is commonly attached to the protein backbone via GlcNAc. The mannose residue is branched and connected via α1,3- and α1,6-glycosidic linkages to the two other mannose building blocks. Based on the chitobiose core, there are three types of *N*-glycans: high mannose, complex, and hybrid (Figure 18A). In high-mannose *N*-glycans, the mannose antennae are extended by further mannose building blocks, whereas in complex *N*-glycans the mannose antennae are extended by GlcNAc and subsequently other monosaccharide units (e.g., Gal). In hybrid *N*-glycans, the C6-antenna is extended by mannose building blocks and the C3-antenna similarly to complex antennae. The core can also be extended by a bisecting GlcNAc (β1,4) at the intermediary mannose, and its GlcNAc residues can be α1,6-fucosylated (Figure 18B). Commonly, *N*-glycans have two, three, or four antennae (Figure 18C). In complex *N*-glycans, the mannose residues can be extended by a GlcNAc residue by a β1,2-, β1,4-, or α1,6-glycosidic linkage. The GlcNAc residues are in turn commonly substituted by β1,4-linked Gal that can subsequently be substituted with *N*-acetylneuraminic acid via an α2,6- or α2,3-glycosidic linkage. The antennary Gal and GlcNAc residues can be α1,2- or α1,3-fucosylated, respectively. Further modifications of the antennae are possible, such as, for example, the substitution with multiple LacNAc (PolyLacNAc) moieties that can in turn also be branched. Furthermore, other fucosylation and sialylation patterns, substitution with blood group epitopes, as well as sulfations are possible.^301^

The analysis of *N*-glycans by means of chromatography often coupled to mass spectrometry is possible and established in many laboratories. Experimental retention times can be matched to values stored in databases and structures assigned.^302^ Although this technique has proven to be reliable, it does not always yield sufficient structural information about the analytes. In the past decades, the usage of mass spectrometry-based techniques for structural characterization of *N*-glycans has become a valuable tool both coupled to chromatography and as a stand-alone technique. Although it is possible to ionize the *N*-glycopeptide analogues of *N*-glycans by fast-atom bombardment (FAB) mass spectrometry,^54^ it was not feasible to ionize underivatized *N*-glycans efficiently using this technique. This changed when ESI and MALDI were introduced, which exhibit an increased sensitivity toward both native and derivatized carbohydrates.^303−305^

Mass spectrometry yields mass-to-charge ratios of ionized *N*-glycans. From the resulting masses, it is commonly possible to estimate the number of monosaccharide units that constitute the *N*-glycan. However, monosaccharide units such as Man and Gal or GlcNAc and GalNAc exhibit identical masses, and discrimination purely based on mass spectrometry is therefore usually not possible. Furthermore, information about branching and the configuration of glycosidic linkages cannot be obtained. By combining MS and various fragmentation techniques, IMS, and spectroscopic techniques, multidimensional information can be obtained to resolve the aforementioned issues. Here, we will give a brief overview of recent works employing CID, MS^*n*^, ExD, UVPD, IM-MS, and IR spectroscopy for in-depth structural analysis of *N*-glycans.

### 4.2 Collision-Induced Dissociation of *N*-Glycans

CID is the most common fragmentation method. It is a slow activation method, where the weakest bonds are cleaved first. Based on the formed fragments, information about the connectivity of the monosaccharide units can be gained. Depending on whether cross-ring fragments (A and X) are formed, additional information about linkage and branching can be obtained.^306^ However, the fragmentation patterns are strongly dependent on whether the spectra are recorded in positive or negative ion mode. Moreover, the information content obtained from CID of *N*-glycans depends on the type of adducts that are fragmented (protonated ions, metal adducts, or phosphate adducts) and whether the *N*-glycan is derivatized or labeled. It must be noted that activation by IRMPD essentially yields the same fragments as CID.^307^

An early report shows that CID of sodiated isomeric and permethylated high-mannose *N*-glycans leads to distinct fragmentation patterns upon which some of the isomers can be discriminated.^305^ It was shown that cross-ring fragments can be formed, and increased branching of *N*-glycans leads to wealthier fragmentation patterns. However, in complex or hybrid *N*-glycans, diagnostic cross-ring fragments are formed at a much lower abundance. Harvey studied CID of derivatized and underivatized *N*-glycans as protonated ions and sodium adducts generated by ESI-MS. CID of [M + H]^+^ mainly yields glycosidic cleavage fragments, whereas for [M + Na]^+^ additional diagnostic cross-ring fragments can be detected. Also, the degree of linkage information by cross-ring fragments is higher in mass spectra of high mannose than of complex *N*-glycans. Complex *N*-glycans preferentially fragment at the glycosidic linkage of GlcNAc residues instead of yielding cross-ring fragments.^94,308,309^ CID of silver-adducted underivatized *N*-glycans, formed during ESI-MS, promotes the cleavage of glycosidic bonds. However, contrary to [M + Na]^+^ adducts, no diagnostic cross-ring fragments are formed.^310^

In positive ion mode CID, cross-ring fragments (A/X) that are diagnostic for the structure can only be obtained for certain metal adducts, usually at low abundance. The issue can be circumvented in negative ion mode CID. Harvey showed that CID of derivatized *N*-glycosidic [M – H]^−^ ions leads to diagnostic A-, Y-, and Z-fragments, which enable unambiguous identification of the type of *N*-glycan and certain structural motifs. The abundance of cross-ring fragments in these spectra is rationalized by competitive loss of a hydrogen from a hydroxy group.^311^ The fragmentation behavior in negative and positive ion modes was confirmed for derivatized *N*-glycans using MALDI-MS by Wuhrer et al.^312^ In contrast to other anionic adducts, [M + NO_3_]^−^ adducts, formed by ESI, tremendously increased the sensitivity, and CID yielded diagnostic C- and A-fragments, contrary to the less diagnostic B- and Y-fragments that are commonly observed in the positive ion mode.^313−315^ A similar fragmentation behavior has been determined for [M + H_2_PO_4_]^−^ adducts.^313,316^ Domann et al. found that ionization of *N*-glycans in the negative mode by MALDI does not readily occur. By using a 2,4,6-trihydroxyacetophenone (THAP) matrix, they were able to efficiently form [M – H]^−^ ions, and subsequently LIFT-CID yielded comparable spectra to ESI/CID.^317^

It is not straightforward to ionize acidic *N*-glycans (e.g., sialylated or sulfated) in positive ion mode. Generally, sialic acids (in mammals only neuraminic acid) are lost during ionization in positive ion mode, which can, however, be circumvented by prior esterification.^318^ Clean MS and MS^2^ spectra of sialylated species in positive ion mode were obtained by MALDI-MS.^319^ Reiding et al. showed that α2,3- and α2,6-sialylated complex *N*-glycans can be distinguished by MALDI in positive ion mode after esterification.^320,321^ Ionization of these species is facilitated in negative ion mode, but does it help to elucidate *N*-glycan structures? Wheeler and Harvey were able to discriminate two isomeric complex disialylated *N*-glycans based on the MS^2^ (and MS^3^) CID fragmentation patterns of the [M – 2H]^2–^ ions and their C_4_ fragments generated via ESI-MS.^322^ Seymour et al. emphasized the difference in CID spectra obtained from sialylated and asialylated *N*-glycans in negative ion mode ESI-MS. Due to charge localization, the MS^2^ spectra of sialylated *N*-glycans are much less comprehensive than their asialylated counterparts.^323^ Using a similar approach based on PGC-LC-ESI-MS/MS and CID combined with the Skyline software,^324^ Ashwood et al. were able to identify the linkage and branching positions of sialic acids on *N*-glycans ionized as [M – H]^−^.^325^ Sulfated *N*-glycans exhibit similar fragmentation patterns as sialylated compounds, due to the localized charge in their [M – H]^−^ ions.^315,326^

Wuhrer et al. determined that Fuc and several other hexoses in *N*-glycans can migrate under tandem MS conditions in both ESI- and MALDI-MS, which may lead to potentially erroneous structure assignments. The phenomenon only occurs for protonated ions and ammonium adducts in positive ion mode.^327,328^ Fuc and bisecting GlcNAc moieties can be reliably determined by the fragmentation pattern observed in negative ion mode CID of [M + NO_3_]^−^ and [M + H_2_PO_4_]^−^ adducts.^315,329^ Sulfated and fucosylated complex *N*-glycans were characterized in positive and negative ion modes via MALDI-MS/CID.^330^ Zhou et al. analyzed permethylated *N*-glycans as [M + H]^+^ using PGC-LC-ESI-MS/MS. Based on the collisional fragmentation patterns, a diagnostic ion for core fucosylation is reported, and β1,3- and β1,4-linked Gal could be discriminated.^331^

CID of *N*-glycans in the positive and the negative ion mode studied by ESI- and MALDI-MS has been extensively reviewed.^95,332,333^

### 4.3 Characterization of *N*-Glycans by MS^*n*^ Methods

MS^*n*^ essentially yields the same information as CID, but controlled sequential collisional fragmentation provides more details about the parent ion. Sheeley et al. and Weiskopf et al. showed that branching patterns and linkage data can be obtained by MS^*n*^ of high-mannose and complex sodiated and permethylated *N*-glycans generated by ESI-MS.^334,335^ AP-MALDI experiments led to sodiated *N*-glycans [M + Na]^+^ and showed that multistage experiments are possible without permethylation or other derivatizations.^336^ Harvey et al. showed that high-mannose, complex, and hybrid [M + Na]^+^*N*-glycans can be generated by MALDI. Here, MS^*n*^ helps the understanding of the mechanisms leading to fragmentation, facilitating interpretation of tandem mass spectra.^337^ Lapadula et al. presented an algorithm for processing MS^*n*^ spectra of oligosaccharides, as shown for permethylated *N*-glycans, that helps to reconstruct glycan structures based on the MS^*n*^ spectra, without biosynthetic constraints or comparing against previously reported structures.^338−340^ Ashline et al. showed that MS^*n*^ of permethylated sodium-adducted *N*-glycans leads to the same fragments as chemically synthesized epitope precursors.^341^ Fucosylation positions and fragments indicative for polylactosamine could be identified by MS^*n*^.^342^ In conclusion, MS^*n*^ leads to a higher degree of information than simple fragmentation via CID. Reliable structural assignment based on algorithms interpreting MS^*n*^ data is possible. However, recording MS^*n*^ spectra comes at the cost of higher sample consumption and is therefore not always a viable option.

### 4.4 Electron-Based Dissociation Methods in *N*-Glycan Analysis

ExD often delivers complementary information to CID or IRMPD for carbohydrates. While in slow activation methods (e.g., CID and IRMPD) usually the weakest bonds are cleaved first, ExD methods are fast activation methods, where a bond in close proximity to the site of electronic excitation is broken. Commonly, the techniques involve multiply charged species that still carry a charge after an electron is attached to or detached from the precursor ion.

Adamson et al. compared the IRMPD and ECD fragmentation spectra of a complex *N*-glycan. In ECD, electrons from a low-energy electron source are captured by a multiply charged cation, leading to fragmentation. For *N*-glycans, the fragmentation efficiency was observed to be very weak so that vibrational activation by IRMPD was necessary before ECD fragmentation. It is suggested that the low fragmentation efficiency is connected to the secondary structure of the *N*-glycan, as ECD fragmentation was not an issue in linear glycans. Because most of the fragments obtained by activated ECD are identical to those obtained by IRMPD, the authors expected them to be formed rather by vibrational excitation. Metal-adducted *N*-glycans, e.g., [M + Co]^2+^, exhibited richer fragmentation patterns than their protonated counterparts.^85^ Zhou et al. compared ECD and IRMPD spectra obtained from Ca^2+^-, Co^2+^-, and Mg^2+^-adducted sulfated hybrid *N*-glycans. IRMPD generally leads to cleavage of the labile sulfate group, while it is retained after ECD, which allows us to determine the sites of sulfation.^343^ Zhao et al. probed permethylated high-mannose and complex *N*-glycans (sialylated and asialylated) with hot ECD, where the electrons are higher in energy than in common ECD. Triply and doubly sodiated adducts mainly yielded C- and Z-ions but also A- and X-fragments, which helped to determine branching and sialylation patterns.^344^ Yu et al. compared ECD (1.5 eV), hot ECD (9 eV), and EED (14 eV) for a high-mannose *N*-glycan as [M + 2Li]^2+^ adducts. In EED, the cations are irradiated with electrons (>9 eV), leading to much richer fragmentations than ECD. In the study, most cross-ring fragments were obtained from EED, so that it was possible to find the positions of five out of six glycosidic linkages.^345^ Wei et al. used a PGC-LC-EED MS/MS workflow for the *de novo* analysis of glycan structures from isomeric high-mannose *N*-glycans with a software called GlycoDeNovo.^346^ The method can also be used for quantification.

**Figure 19:**
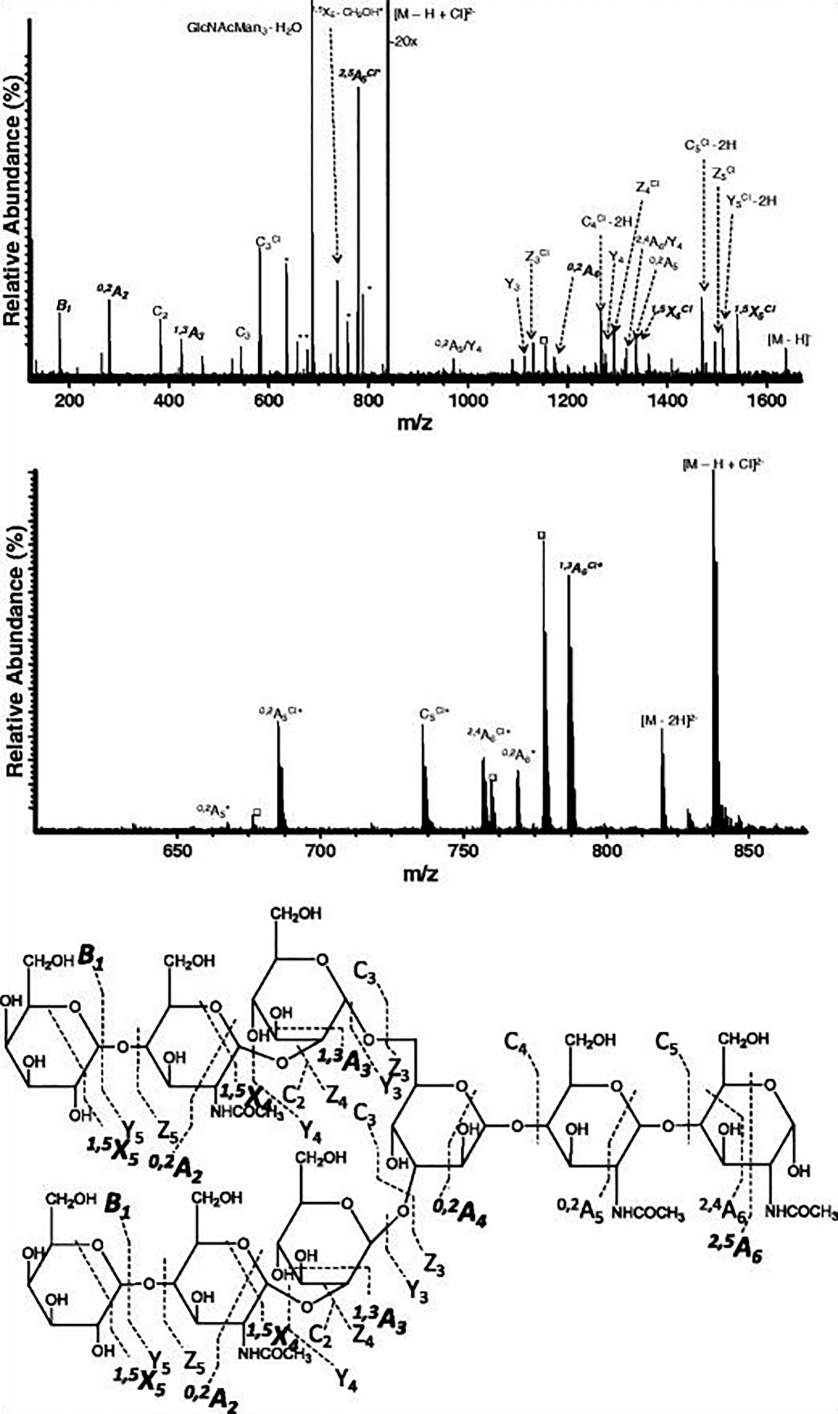
Fourier transform ion cyclotron resonance tandem mass spectra of a deprotonated and chlorinated asialylated complex biantennary *N*-glycan, [M – H + Cl]^2–^. Mass spectrum employing (top) EDD and (middle) CID on the precursor ion [M – H + Cl]^2–^. Product ions shown in bold are unique to fragmentation of [M – H + Cl]^2–^ compared to the doubly deprotonated species [M – 2H]^2–^. An asterisk indicates doubly charged product ions, whereas squares indicate water loss from adjacent product ions. Product ions bearing a chloride anion are highlighted with superscripted Cl. (bottom) observed fragmentation pattern after EDD of [M – H + Cl]^2–^. Reprinted with permission from reference.^269^ Copyright 2012 American Society for Mass Spectrometry.

Another promising technique for *N*-glycan analysis is EDD of multiply charged anions. Similar to cations in ECD, the anions are irradiated by low-energy electrons, leading to electron detachment and subsequent fragmentation. Besides the commonly obtained B/C/Y/Z-fragments, this technique also yields more diagnostic A- and X-fragments, such as ^1,5^A, ^3,5^A, ^1,5^X, and ^3,5^X (contrary to ^0,2^A and ^2,4^A observed in negative ion CID/IRMPD), in high abundance. Both asialylated and sialylated complex *N*-glycans were probed as doubly charged deprotomers. Based on the fragments, core fucosylation could be reliably determined.^268^ In acidic-labeled sialylated complex *N*-glycans, the EDD spectra are less rich in fragments, probably due to the location of the charge, altered by the label.^347^ EDD of a chloride-adducted, deprotonated asialylated complex *N*-glycan [M – H + Cl]^2–^ leads to extensive fragmentation compared to CID (Figure 19).^269^

### 4.5 Ultraviolet Photodissociation Mass Spectrometry of *N*-Glycans

In UVPD, the probed ions are electronically excited by UV photons (10–400 nm), leading to fast activation with subsequent fragmentation. Devakumar et al. analyzed permethylated high-mannose and (asialylated and core fucosylated) complex *N*-glycans as [M + Na]^+^ ions (Table 1). Here, 157 nm UVPD leads to a considerable amount of A- and X-fragments by which connectivity of the monosaccharide units can be determined. However, also B-, C-, Y-, and Z-fragments can be observed.^348^ In a second study, the same authors showed that 157 nm UVPD in combination with ion trap MS^*n*^ is able to analyze isomeric *N*-glycans released from ovalbumin due to extensive cross-ring fragments.^349^ Ko et al. analyzed mono- and disialylated complex *N-*glycans as [M – H]^−^ and [M – 2H]^2–^, respectively, with CID and 193 nm UVPD. In contrast to their CID counterparts, the obtained UVPD fragmentation patterns are generally very rich and contain a lot of signals for cross-ring A- and X-fragments, on the basis of which sialylation patterns can be analyzed.^76^

**Table 1:** Fragments and Informational Content Delivered by Applying Diverse Fragmentation Techniques in MS to *N*-Glycan Ions

technique (ion mode)	species	fragments	diagnostic information	references
CID (+)	[M + H]^+^	B/Y	composition, sequence	b,^308^; a,^94^
CID (+)	[M + Na]^+^	(A)/B/Y	composition, sequence, (connectivity, branching)	b,^308,309^; a,^94^
CID (+)	[M + Ag]^+^	B/Y	composition, sequence	a,^310^
CID (−)	[M – H]^−^	A/Y/Z	composition, sequence, connectivity, branching	b,^311^
CID (−)	[M + NO_3_]^−^	A/C	composition, sequence, connectivity, branching	a,^313,315^; b,^314^
CID (−)	[M + H_2_PO_4_]^−^	A/C	composition, sequence, connectivity, branching	a,^313,316^
IRMPD (+)	[M + 2H]^2+^	B/C/Y/Z	composition, sequence	a,^85^
IRMPD (+)	[M + Co]^2+^	A/B/C/Y/Z	composition, sequence, connectivity, branching	a,^85,343^
IRMPD (−)	[M – 2H]^2–^	A/B/C/Y/Z	composition, sequence, connectivity, branching	a,^268^; b,^347^
ECD (+)	[M + 2H]^2+^	(A)/B/C/Y/Z	composition, sequence, (connectivity, branching)	a,^85^
ECD (+)	[M + Co]^2+^	A/B/C/X/Y/Z	composition, sequence, connectivity, branching	a,^85,343^
hot ECD (+)	[M + 2Na]^2+^/[M + 3Na]^3+^	A/B/C/X/Y/Z	composition, sequence, connectivity, branching	c,^344^
ECD (+)	[M + 2Li]^2+^	A/B/C/Y/Z	composition, sequence, connectivity, branching	c,^345^
EED (+)	[M + 2Li]^2+^	A/B/C/X/Y/Z	composition, sequence, connectivity, branching	c,^345^
EDD (−)	[M – 2H]^2–^	A/B/C/X/Y/Z	composition, sequence, connectivity, branching	a,^268^; b,^347^
UVPD (+)	[M + Na]^+^	A/(B/C)/X/(Y/Z)	composition, sequence, connectivity, branching	c,^348^
UVPD (−)	[M – H]^−^	A/B/C/X/Y/Z	composition, sequence, connectivity, branching	a,^76^

a Not derivatized.

b Labeled

c Permethylated.

All fragmentation techniques yield valuable information on the structure of *N*-glycans. However, there are some limitations. Analysis of mass spectra of isomeric *N*-glycan mixtures is still difficult, which is the reason why separation by chromatography prior to MS is still necessary. Although some isomers yield distinct fragmentation patterns, databases and/or expert knowledge are furthermore required for structural assignments.^350^ Separation by chromatography can, however, be replaced with a separation technique in the gas phase, namely, IMS. The technique can be easily coupled to various ionization sources and MS instrumentation.

### 4.6 Ion Mobility–Mass Spectrometry in *N*-Glycan Analysis

**Figure 20:**
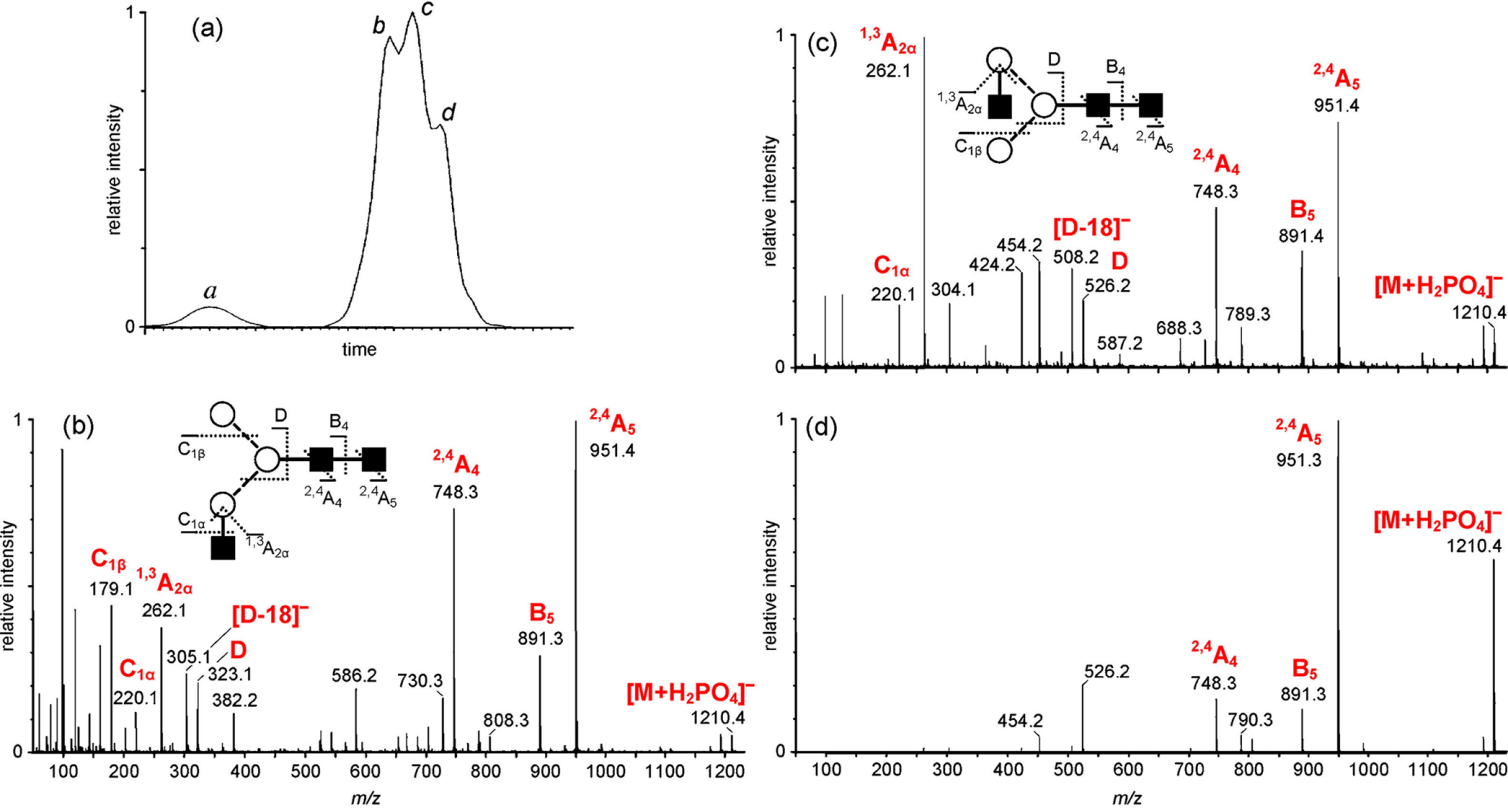
Ion mobility separation with subsequent collision-induced dissociation (CID) of a mixture of complex *N*-glycans as [M + H_2_PO_4_]^−^ ions. (a) TWIM-MS ATD for GlcNAc_1_Man_3_GlcNAc_2_ released from chicken albumin. CID tandem mass spectra for the features (b) *b*, (c) *c*, and (d) *d* in the ATD. Reprinted with permission from ref (278). Copyright 2009 Elsevier B.V.

Preliminary studies showed that compositional, configurational, and linkage isomers of glycans can be distinguished by IM-MS.^133,351−353^ The advantage of IM-MS over MS is that ions are separated not only by their mass and charge but also by their size and shape. How can these results be transferred to *N*-glycans? Isailovic et al. provided the first evidence that it is possible to separate permethylated high-mannose and complex *N*-glycan isomers as [M + Na]^+^ ions using DTIM-MS.^354^ Plasencia et al. were able to resolve and assign structural isomers of permethylated high-mannose and hybrid *N*-glycans from ovalbumin ionized as [M + Na]^+^ and [M + 2Na]^2+^ adducts using DTIM-MS.^355^ Similar approaches were used by others to separate labeled and nonlabeled *N*-glycans released from biological samples with^356^ or without^357−361^ prior chromatographic separation. Here, the IM separation made it possible to measure peptides and glycans from the same sample by “cleaning up” the mass spectra. Thus, IM-MS can reliably separate different groups of molecules with similar *m*/*z*. Williams et al. characterized [M + Na]^+^ and [M + H_2_PO_4_]^−^ adducts of high-mannose and complex *N*-glycans using DTIM-MS and TWIM-MS. It is shown that for the same *N*-glycan released from different proteins the same drift times are obtained. Complex isomeric *N*-glycans were separated and subsequently analyzed via MS/MS (see Figure 20). Thus, depending on which antennae the GlcNAc residue is located, different mobilities are obtained.^278^

Harvey et al. used TWIM-MS to yield glycan profiles of underivatized *N*-glycans (high-mannose, hybrid, complex, bisecting GlcNAc, core fucosylated, and sialylated) released from various proteins and determined that isobaric *N*-glycans can be separated based on their drift times in positive ion mode as [M + Na]^+^ ions and in negative ion mode as [M + Cl]^−^, [M + NO_3_]^−^, and [M + H_2_PO_4_]^−^ ions followed by CID.^362^ The technique can also be applied to differentiate between molecular ions and in-source fragments. However, they conclude that the resolution of IM-MS needs to be increased to unfold its full potential as an analytical technique for separating isomers.^363^ Yamaguchi et al. were able to separate isomeric labeled *N*-glycans by HILIC-TWIM-MS. They determined that the best separation is observed for [M + 2H]^2+^ ions, contrary to [M + H]^+^, [M + H + Na]^2+^, and [M + H + K]^2+^. Although fucosylated samples were also part of the analysis, fucose migration was not taken into consideration.^364^ Zhu et al. analyzed permethylated isomeric high-mannose (Man7) *N*-glycans from ribonuclease B as [M + 2Na]^2+^ adducts. The DTIM-MS arrival time distribution (ATD) of the selected *m*/*z* value showed four features corresponding to each isomer, respectively.^365^

Pagel and Harvey showed that the ATDs of sodiated *N*-glycans [M + Na]^+^ recorded on a second-generation TWIM-MS instrument are superior to the previous generation. They showed that the collision cross sections (CCSs), an instrument-independent parameter of the ion derived from the ATDs, are clearly differentiable for isomeric *N*-glycans. They emphasize using the obtained CCSs as storable parameters in databases for complex carbohydrate analysis.^366,367^ Another IM-MS study from Harvey et al. showed that conformer resolution of *N*-glycans released with endoH or endoS (no terminal GlcNAc) is smaller than that of *N*-glycans released with PNGase F; however, the molecular ions have a similar fragmentation behavior.^368^ Struwe et al. determined the CCSs of high-mannose *N*-glycans measured as [M + H]^+^, [M + Na]^+^, [M + K]^+^, [M – H]^−^, [M + Cl]^−^, and [M + H_2_PO_4_]^−^ adducts using a combination of TWIM-MS and DTIM-MS. [M – H]^−^ ions exhibited an unusually broad and multimodal ATD, which provided evidence for the coexistence of different conformers.^369^ Reduction of the reducing end to alditol, however, later showed that the broad ATD results from reducing end anomers rather than the presence of multiple conformers.^370^

**Figure 21:**
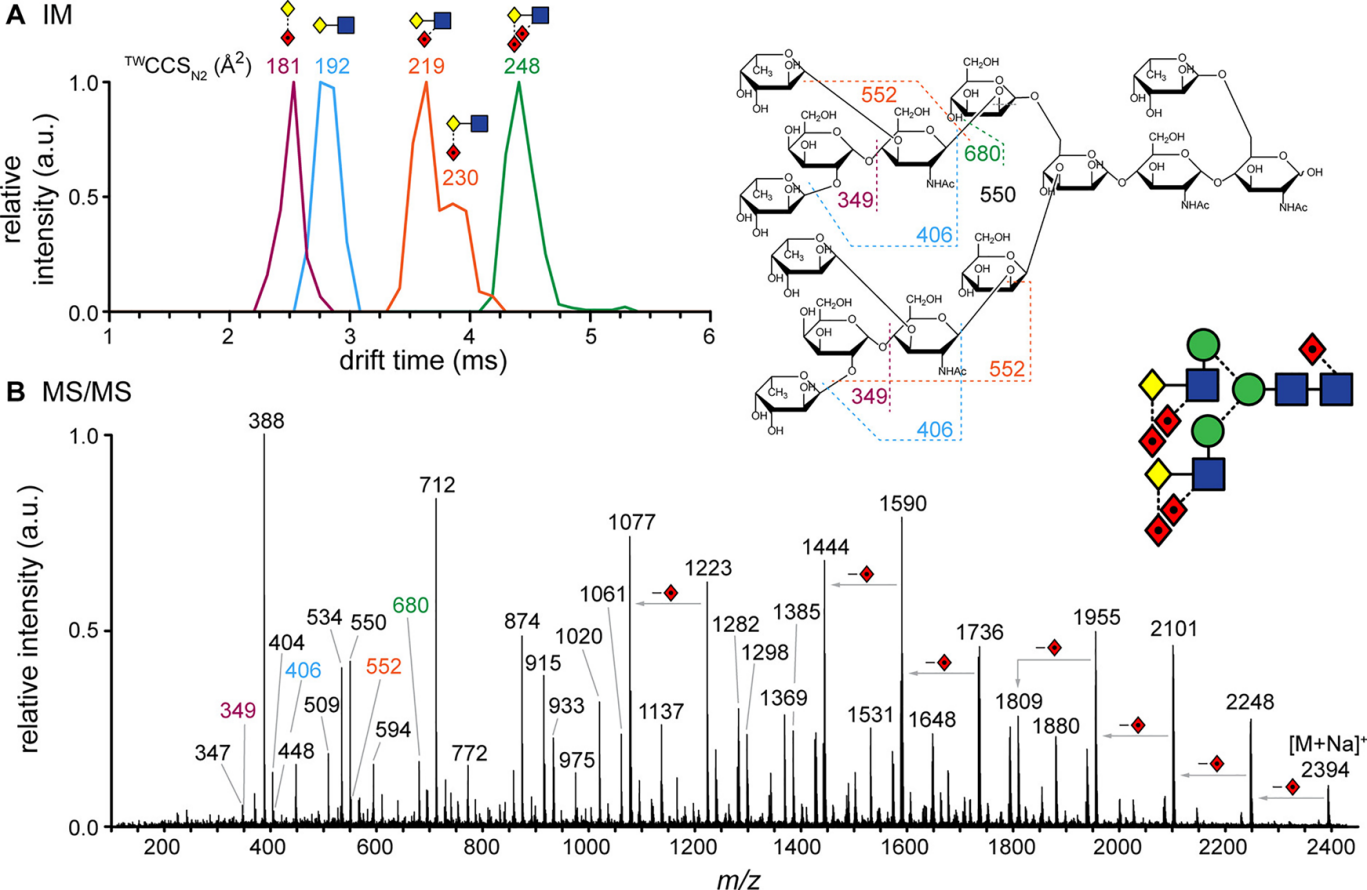
TWIM-MS/MS of sodiated complex *N*-glycan from a human parotid gland. (A) ATDs of diagnostic fragments with ^TW^CCS_N2_. (B) Tandem mass spectrum of [M + Na]^+^, including the representation of the precursor structure and fragment assignments. Glycan structures are represented using the Oxford system (notable differences to SNFG nomenclature: yellow diamond = Gal, red diamond = Fuc). Reproduced with permission from ref (374). Copyright 2017 American Chemical Society.

Zhu et al. measured the CCSs (DTIMS-MS) of doubly charged metalated, permethylated high-mannose *N*-glycans [M + X]^2+^, where X = Mn, Fe, Co, Ni, Cu, Mg, Ca, and Ba. The ATDs suggest the presence of multiple conformers, arising from the metal binding to different sites of the *N*-glycan. Using MS/MS of ATD-selected conformers, the location of the binding site was assessed, leading mainly to B- and Y-fragments. Interestingly, larger cations do not necessarily lead to higher CCSs, as determined for Ba^2+^ and Ca^2+^. The former presumably binds to multiple sites, leading to an overall tighter conformation.^371^ Harvey and Abrahams measured the arrival times (TWIM-MS) of a large set of reduced high-mannose, complex, and hybrid *N*-glycans in the negative ion mode as [M + H_2_PO_4_]^−^ adducts. Compared to nonreduced glycans, two main differences were observed: (1) fragmentation of the chitobiose core was less abundant and (2) the observed ATDs were more symmetric than for nonreduced compounds. The authors therefore concluded that the asymmetry was due to anomer separation.^370^ Harvey et al. determined that isomeric separation (TWIM-MS) of hybrid and complex *N*-glycans is possible in positive and negative ion mode. Although the separation seems to be marginally better in positive ion mode, the tandem mass spectra give more informative in negative ion mode.^372^ In another study, the authors show that the separation of high-mannose *N*-glycans by the same method is less distinct. This issue was resolved using an instrument with higher resolution.^373^ Hofmann et al. analyzed pentafucosylated biantennary complex *N*-glycan in positive ion mode as sodium adducts with IM-MS/MS. Based on the CCSs of the obtained fragments, it was possible to determine that the antennae are substituted with the Le^*y*^ epitope (Figure 21).^374^ Harvey and Struwe reported that TWIM-MS/MS of complex and hybrid fucosylated *N*-glycans measured as [M + H_2_PO_4_]^−^ can reveal the presence or absence of bisecting GlcNAcs, branching patterns of antennae, and the location of fucose.^375,376^ Similar findings were reported by Harvey et al. for [M + H_2_PO_4_]^−^ anions of high-mannose *N*-glycans that produce fragments with unique CCSs under CID conditions that can be used for assignment of isomers.^377^ Re et al. and Toraño et al. correlated the CCSs of complex *N*-glycans as [M + H]^+^ and [M – H]^−^ ions. In the negative ion mode, the sialylation pattern of these ion can be readily distinguished by their unique CCS. Furthermore, they show that *N*-glycans can be sampled via force field molecular dynamics to obtain theoretical CCS values of *N*-glycans and predict their conformational distribution in the gas phase.^219,378^ Pallister et al. present an LC-TWIM-MS/MS approach for high-throughput analysis of labeled complex *N*-glycans in the positive ion mode ([M + Na + H]^2+^). Based on the CCSs of *N*-glycans and their fragments, isomeric *N*-glycans can be comprehensively differentiated.^379^ Wei et al. utilized a TIMS instrument to study a permethylated complex and hybrid *N*-glycans measured as [M + 2Na]^2+^. From the data, it can be concluded that many mobility features of *N*-glycans are due to distinct conformers and not structural isomerism. EED of the conformer-selected *N*-glycans provided many characteristic fragments, showing that the fragmentation mechanism is similar for different conformers of the same molecule.^380^

### 4.7 Gas-Phase Ion Spectroscopy of *N*-Glycans

**Figure 22:**
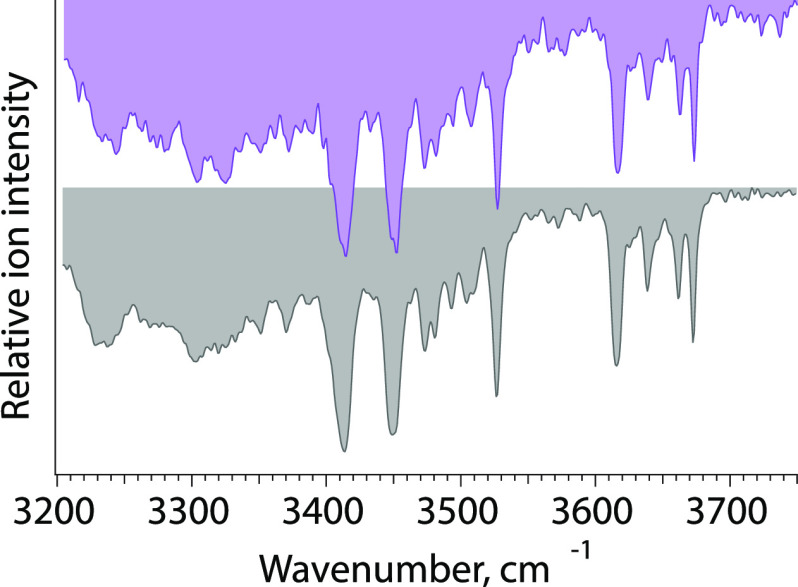
Comparison of IR spectra of protonated complex *N*-glycans. IR spectrum of the GlcNAc_2_Man_3_GlcNAc_2_ reference (purple) and of GlcNAc_2_Man_3_GlcNAc_2_ after enzymatic cleavage from Gal_2_GlcNAc_2_Man_3_GlcNAc_2_ (gray). Figure adapted with permission from ref (382). Copyright 2020 American Chemical Society.

While there are many studies on IM-MS of *N*-glycans, gas-phase spectroscopic studies have remained scarce. UV spectra of mass-selected *N*-glycans have not been reported to date, and their IR action spectroscopic investigation is still in its infancy. Using this method, the vibrational modes of the ions can be excited by IR photons, leading to a characteristic spectrum unique to each ion. Depraz Depland et al. measured gas-phase IR spectra using IRMPD of isomeric sialylated epitopes that can be commonly found on the antennae of *N*-glycans. Infrared spectra were recorded in the 3 μm range of protonated ions and sodium adducts based on which α2,3- and α2,6-sialylated isomers can be clearly distinguished.^381^ Likewise, Mucha et al. used IR spectroscopy in helium nanodroplets in the 5–10 μm range to study fucosylated trisaccharide epitopes, showing that fucose migration in protonated glycans is a universal phenomenon in mass spectrometry. By probing the sodium adducts of these epitopes, it was, however, possible to clearly distinguish epitopes with distinct fucosylation patterns.^205^ Dyukova et al. used messenger-tagging spectroscopy with N_2_ for recording IR spectra of complex *N-*glycans before and after enzymatic cleavage and compared the IR signatures to those obtained from synthetic glycan standards in the 3 μm range. With increasing size of the glycan, the spectra become more complex. However, based on the spectra it can be clearly seen that the signatures of the synthetic standards and the enzymatically cleaved sugars are identical (Figure 22).^382^ Yalovenko et al. combined SLIM-IM-MS and messenger-tagging spectroscopy with N_2_ to study protonated complex (and fucosylated) *N*-glycans in the 3 μm range. With the presented approach, IR signatures with a spectral window of 300 cm^–1^ can be obtained within 55 s, which allows incorporation of this method into analytical workflows.^202^ Based on the findings, the authors emphasize the ultimate goal to compile a database of IR signatures of intact *N*-glycans and their fragments. In a recent study, Dyukova et al. employed the same setup to distinguish complex and fucosylated *N*-glycan positional isomers cleaved from monoclonal antibodies as sodium adducts.^383^

## 5 Mucin-Type *O*-Glycans

### 5.1 Structure and Analytical Challenges

*O*-Glycans are highly abundant in extracellular proteins. The largest number of *O*-glycans occurs on mucins: large proteins that often carry hundreds of *O*-glycosylation sites and which are found throughout the body. Therefore, the involved oligosaccharide structures are often termed mucin-type *O*-glycans. However, *O*-glycosylation is not exclusive to mucins and is also common among other glycoproteins such as immunoglobulins. Mucins are characterized by a large number of tandem repeat domains that are rich in Ser, Thr, and Pro residues, giving rise to a vast number of *O*-glycosylation sites. Extensive *O*-glycosylation in mucins can make up 50 to 80% of the overall mass of the glycoprotein.^384^ This often has a tremendous impact on the overall properties of the molecule. In comparison to the protein backbone, oligosaccharides are generally more hydrophilic and, depending on their terminal modifications, often negatively charged. As a result, *O*-glycans are usually extensively solvated by water and salt ions, which induce a high viscosity and gel-like structure of the mucus. The crucial impact of glycans on the physiochemical properties becomes especially obvious when *O*-glycosylation is altered, for example, in diseases. The certainly most prominent example for such a disease is cystic fibrosis (CF). Here sulfation and sialylation of sputum mucins are significantly increased, while a decreased sialylation/increased fucosylation is observed in membrane-embedded mucins of airway cells.^385^ This drastically increases the viscosity of the mucus in the lungs and leads to obstruction and malfunction of the mucus as a pathogen barrier, which in turn can result in infections with bacteria such as *Pseudomonas aeruginosa*.

*O*-Glycosylation occurs at hydroxyl groups of Ser and Thr residues. It is initiated by enzymatic transfer of an *N*-acetylgalactosamine (GalNAc) residue in the Golgi apparatus. There is a relatively special modification with a single *N*-acetylglucosamine, often simply termed *O*-GlcNAcylation, which will not be covered here as there are no oligosaccharides involved.^386^ In contrast to *N*-glycosylation, no defined sequons are required for *O*-glycosylation. In addition, biosynthesis occurs stepwise and does not include trimming, i.e., cleavage of certain residues by glycosidases during the final processing steps. As a result, *O*-glycans are typically smaller in size compared to *N*-glycans; however, the underlying structural space is vast with multiple sources of isomerism.

**Figure 23:**
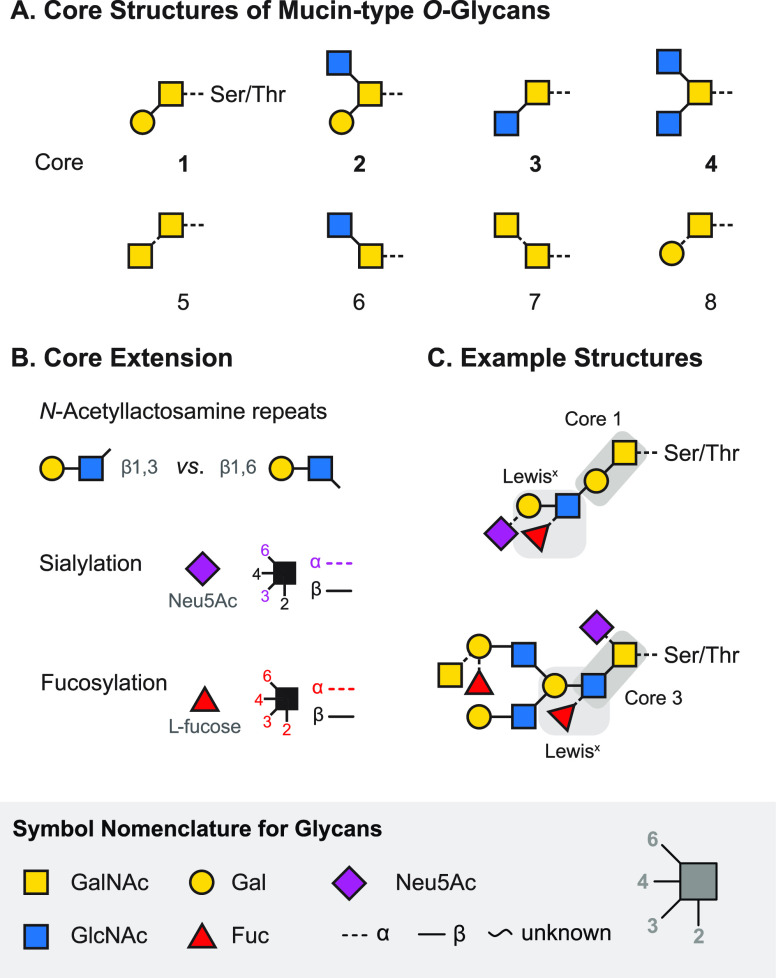
Typical *O*-glycan structures. (A) The most abundant *O*-glycan core structures 1–4 and the four less common cores 5–8 shown using the symbol nomenclature for glycans (SNFG). (B) Typical extensions of glycan cores are the attachment of *N*-acetyllactosamine units, sialylation, and fucosylation. (C) Examples for fully processed *O*-glycan structures based on core 1 (upper structure) and core 3 (lower structure) including their linkage site to the glycoprotein. At the nonreducing end, *O*-glycans often contain antigenic structures such as the ABO and Lewis blood group determinants, which are highlighted by gray boxes.

Generally, *O*-glycans are extended following four major core structures (Figure 23A, first row). Starting from the GalNAc linked to Ser or Thr residues, attachment of β1,3Gal leads to core 1; further attachment of a β1,6-linked GlcNAc to core 1 gives rise to core 2. Both are by far the most common core structures in *O*-glycosylation and are found throughout the body. Cores 3 and 4 are formed by subsequent attachment of a β1,3GlcNAc and a β1,6-linked GlcNAc and are more exclusive to gastric and lung glycoproteins, in particular mucins. In addition, a further four rare core structures have been observed (Figure 23A, second row). However, their underlying biosynthesis and further processing are still poorly understood, and it cannot be excluded that they are arising from enzyme side reactions.^387^ The exceptional structural diversity of *O*-linked glycans is generated by further extension of the core structures, for example, with β1,3- and β1,6-linked *N*-acetyllactosamine (LacNAc) repeats, sulfation, sialylation, and/or fucosylation (Figure 23B). The resulting glycans are highly complex and often contain antigenic structures at their nonreducing termini such as the ABO and Lewis blood group determinants (Figure 23C).

The analysis of *O*-glycosylation is generally more challenging than that of *N*-glycosylation. As mentioned above, *O*-glycosylation does not follow defined sequons. Statistically, prolines are often found adjacent to *O*-glycosylation sites; however, this is a mere statistical effect and is of little analytical use. *O*-Glycosylation positions within a protein are therefore considerably more challenging to identify than *N*-linkages. To make matters worse, there are no specific enzymes, such as PNGaseF in the case of *N*-glycans, that specifically and selectively hydrolyze the glycosidic bond to the protein. Instead, rather harsh chemical procedures are usually employed. The linkage between GalNAc and Ser/Thr residues is labile under basic conditions, such that β-elimination can be used for their release. Usually, this is combined with a reductive workup with NaBH_4_ to reduce side reactions often referred to as “peeling”,^388^ yielding stable oligosaccharides with *N*-acetylgalactosaminitol at the reducing end. As a downside, treatment with NaBH_4_ can lead to partial degradation of the peptide backbone, and the resulting alditols are chemically inactive at the reducing end. In order to reduce the complexity of the resulting mixture, the *O*-glycans can furthermore be trimmed prior to release, for example, by removal of the terminal sialic acid using sialidases. For more details on *O*-glycan release reactions and nonreductive procedures, the reader is referred to recent review articles.^388−390^ Analytical approaches to study intact *O*-linked glycopeptides are briefly discussed in the glycopeptide chapter of this review.

Following the release, *O*-glycans are usually purified and/or separated using chromatography. Reversed-phase (RP) chromatography only shows a poor separation of *O*-glycans due to their high polarity and often negative charge. Hydrophilic interaction liquid chromatography (HILIC)^391^ and porous graphitized carbon liquid chromatography (PGC-LC)^392^ on the other hand are much better suited and often show a remarkable separation even for isomeric structures.^393^ However, all chromatographic techniques have to cope with a fundamental limitation: due to the reductive workup, the released products are alditols, which cannot be labeled with UV and fluorescent-active tags at the reducing end. Nonreductive workup on the other hand shows reproducibility issues and often suffers from unpredictable peeling.^390^ As a result, the workflows established for *N*-glycans are only of limited use for the analysis of *O*-glycosides—one of the major reasons that *O*-linked glycomics is substantially lagging behind the advances made in *N*-linked glycomics. Instead, reductively released and chromatographically separated *O*-glycans are usually analyzed directly as alditols or after permethylation using ESI-MS. The molecular identity of the underlying structures is usually obtained using a tedious but often highly informative fragment analysis.^394^ In the following chapter, we will summarize the recent developments in *O*-glycan tandem MS and IM-MS analysis.

### 5.2 Collision-Induced Dissociation and Higher-Energy Collisional Dissociation of *O*-Glycans

CID and higher-energy collisional dissociation (HCD) are the most widespread and therefore most frequently used dissociation techniques in tandem MS. In this regard, *O*-glycan analysis is no exception. Similarly to *N*-glycans, tandem MS experiments are usually preceded by a chromatographic separation, which is directly hyphenated to the mass spectrometer. In contrast to *N*-glycans, however, even the very early MS studies on released *O*-glycans were mostly focused on ions with negative polarity.^392^ On one hand, this is a result of the higher abundance of negatively charged sialic acid and sulfate moieties in *O*-glycans, which generally lead to considerably increased ionization efficiency for negative ions. Especially in nano ESI, also neutral released *O*-glycans tend to ionize well when derived from a reductive workup, i.e., as alditols.^395^ On the other hand—and similarly to *N*-glycans—negatively charged *O*-glycans tend to form C-type fragments, which in turn can rearrange in a retro-aldol reaction into highly diagnostic A-ions. Albeit less pronounced in negatively charged *O*-glycans, such cross-ring cleavages significantly increase the informational content of the resulting fragmentation spectra and facilitate more confident structural assignments.^394^ As a downside, tandem mass spectra of negatively charged *O*-glycans are often very complex, and their interpretation usually requires expert knowledge—an aspect which so far limits applications to a few specialized laboratories.

Mucins and other hydrogel glycoproteins are usually densely *O*-glycosylated. As a result, they exhibit a characteristic structure in which the glycans are sticking out like the brittles of a bottle brush. These brittles shield the protein backbone from the exterior and prevent proteolysis from enzymes such as trypsin. Generating glycopeptides from fully glycosylated mucins is therefore highly challenging, if not impossible. The majority of MS-based glycomics analyses is therefore limited to a profiling of structural features rather than a full, site-specific structural identification. The probed motifs can include core structures, ABO and Lewis blood group motifs, sialylation, and even sulfation.^396^ Often, *O*-glycan profiling is combined with exoglycosidase digestion prior to analysis to remove certain terminal residues such as sialic acids.

Early works on eggs from *Xenopus* frogs showed that characteristic *O*-glycosylation features can be identified based on the CID mass spectra of glycans released from egg jelly and digested with exoglycosidases.^397^ Using tetradeuterioborate instead of tetrahydroborate during the release and workup procedure was furthermore used for comparison and relative quantification of *O*-glycans from different sources.^398^ Later works showed that similar approaches can also be utilized for the profiling of *O*-glycans on isolated and purified glycoproteins.^399^ The underlying workflows are built on the sequential release of *N*- and *O*-glycans from the glycoprotein as well as exoglycosidase digestion. When combined with glycopeptide analysis,^400^ a detailed and site-specific glycosylation profile can be obtained. Furthermore, an approach for the combined release and labeling of *O*-glycans with 1-phenyl-3-methyl-5-pyrazolone (PMP) has been reported.^401^ This is particularly useful for the analysis of released oligosaccharides in positive ion mode. A very recent work showed the exceptional utility and robustness of *O*-glycan profiling for the analysis of gastric mucin samples from patients.^396^ Extensive *O*-glycosylation features of ten individuals were determined. The results revealed that each individual carries more than 100 glycan structures, with up to 14 of them being unique to the particular patient. Even though the number of probed individuals was low, a consistent increase in the level of sialylation and sulfation on gastric *O*-glycans was observed for cancerous tissue.

Very few works deal with the unique mechanistic aspects of *O*-glycan CID fragmentation. A comparison between *O*-glycan alditols and aldoses revealed distinct fragmentation pathways, some with and some without participation of the charge carrier.^402^ This study furthermore revealed that *O*-linked aldoses are present as α/β-anomers—an aspect that was later also observed in IM-MS for other oligosaccharides.^154^ Further works revealed that HCD fragmentation is mechanistically not that different from CID but can clearly help to identify diagnostic fragments that are not accessible by CID, in particular at lower masses. This, for example, enabled the identification of *O*-glycans from fungi that are exclusively composed of hexose and hexuronic acid.^403^ Likewise, low-mass HCD fragments were recently shown to be diagnostic to the regiochemistry of sulfate modification (3 vs 6) in mucin *O*-glycans.^404^

### 5.3 Characterization of *O*-Glycans by MS^*n*^ Methods

*O*-Glycans are highly branched oligosaccharides. An identification of the branched site is not always straightforward using conventional CID experiments, as fragments from branched and linear chains are often isomeric. A strategy to solve this problem is MS^*n*^ of permethylated glycans using ion trap instruments.^350,405^ Here, all accessible OH and *N*-acetylated amide groups are derivatized using reactive methylating agents such as methyl iodide.^406^ Subsequent sequential fragmentation of fragments reveals the number of methylated sites per monosaccharide and with that the relative position of the building block within the glycan network. As a downside, the time and sample consumption in MS^*n*^ experiments is usually high, and a clear annotation of the spectra is elaborate. MS^*n*^ experiments on permethylated *O*-linked glycans are usually performed on sodium adducts and can yield exceptional structural insights, including the identification of isomers.^341,342,407^ Furthermore, MS^*n*^ experiments on nonderivatized glycans were used to unravel the details of *O*-glycan fragmentation and identify more complex structures.^408,409^ As in conventional *O*-glycan CID, exoglycosidases were used to reduce the sample complexity, and ions of negative polarity were investigated.

### 5.4 Ion Mobility–Mass Spectrometry of *O*-Glycans

In contrast to other glycoconjugates, *O*-glycans are only sparsely studied by IM-MS and related methods. This is likely a result of the tedious release procedure and the lack of well-characterized standards, and not least because of their exceptional structural complexity. A series of synthetic *O*-glycan-like structures were investigated as part of a very extensive study on the IM-MS behavior of oligosaccharide isomers.^282^ Here, the impact of ion polarity and adduct formation on the separation of isomeric structures was focused. Very similar to other glycoconjugates, the investigated *O*-glycans did not show clear trends, which would enable a prediction of the best separation conditions. An optimization of the probed ionic species as well as the experimental conditions are therefore required for each individual separation problem. Moreover, the potential of a combination of IM-MS and UVPD was recently tested.^410^ Two synthetic tetrasaccharides were mobility-separated in a DTIMS cell and subsequently subjected to UV radiation from a 193 nm ArF excimer laser. As metal adducts, both isomers are clearly distinguishable by IMS alone. However, UVPD adds an additional dimension in the form of rich tandem mass spectra. This gain in informational content becomes especially apparent, when the obtained UVPD mass spectra are compared with their counterparts from CID.

**Figure 24:**
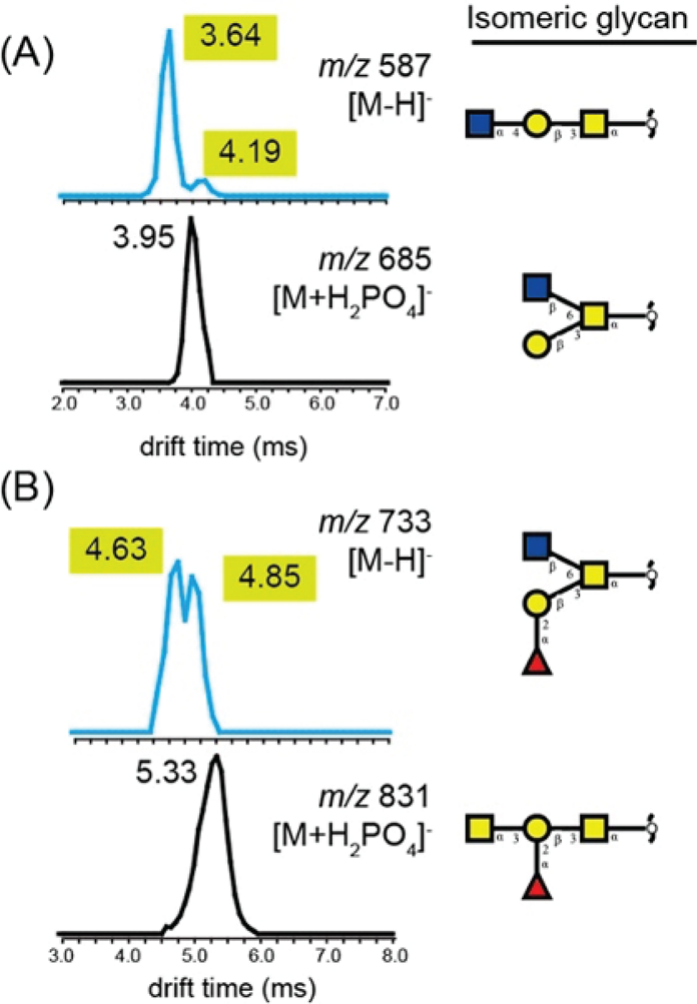
Ion-mobility-based comparison of two sets of *O*-glycan isomers as [M – H]^**–**^ and [M + H_2_PO_4_]^**–**^ ions. (A) Arrival time distributions (ATDs) of two isomers composed of Hex1HexNAc2 measured as deprotonated (*m*/*z* 587, blue) or phosphate adduct (*m*/*z* 685, black) ions. (B) ATDs of two Hex1HexNAc 2dHex1 isomers as deprotonated (*m*/*z* 733, blue) or phosphate adduct (*m*/*z* 831, black) ions. Structures are depicted using the symbol nomenclature for glycans (SNFGs): yellow square = GalNAc, yellow circle = Gal, blue square = GlcNAc, red triangle = Fuc. Figure adapted and modified with permission from ref (411). Copyright 2019 American Chemical Society.

Very recently, IM-MS was extensively used to test the utility of the method in established PGC negative ion LC-MS workflows—the current gold-standard technology for the separation of isomeric *O*-glycans (Figure 24).^411^ In particular, *O*-glycans released from porcine gastric and human salivary mucins were systematically investigated and compared using both methods. The experiments reveal that IM-MS is a promising tool for *O*-glycan structural analysis that is capable of resolving the structural complexity that LC cannot always resolve. The combination of IMS-selected precursor ions and their negative ion MS fragmentation spectra was found to be particularly useful. Moreover, this study presents one of the few examples in which the utility of IMS as a separation technique is directly compared to that of LC. A couple of years ago, this comparison would have been a clear overestimation; however, the resolution of state-of-the-art IMS systems is increasing at a rapid pace, and LC-like separations in IMS are therefore not out of reach any longer.^144^

### 5.5 Bioinformatics Tools

One of the fundamental outcomes of the above-mentioned tandem-MS studies is that the analysis and annotation of glycan fragment spectra are often very tedious and require expert knowledge. A promising approach to tackle this problem is spectral libraries that can be used as references to annotate complex spectra. Due to the universal nature of glycan fragments, this approach is generally not limited to *O*-glycans but rather universal for all glycoconjugate structures that exhibit a comparable CID fragmentation behavior and fragment structure.

The general feasibility of a database approach was exemplified in the LC-ESI-MS/MS analysis over 200 *N*- and *O*-glycans from human saliva glycoproteins.^412^ A library of diagnostic fragment ions was compiled and combined with other specific structural features from cross-ring and glycosidic cleavages. These reference values were subsequently used to characterize and differentiate potential isomers. The success of this study led to the release of UniCarb-DB, a curated open access database containing comprehensive LC MS/MS data of synthetic standards as well as N*-* and O*-*linked glycans released from glycoproteins.^413^ The deposited spectra have been evaluated by independent laboratories and were annotated with glycosidic and cross-ring fragmentation ions, retention times, and associated experimental metadata descriptions. Also IM-MS-derived CCS values of glycoconjugates and their fragments were implemented with UniCarb under the name GlycoMob.^414^ A similar, albeit much less extensive, library of LC and MS/MS data of human milk oligosaccharides was recently released within the NIST framework (https://chemdata.nist.gov/glycan/spectra).^415^

Another important aspect toward routine use is the automation of a reliable spectral assignment and structural identification, ideally with minimal human intervention. The recently reported software tool Glycoforest 1.0 is a first step toward this goal. Glycoforest uses a *de novo* algorithm that is fed with manually annotated tandem mass spectra, either prepared in-house or obtained externally for example from UniCarb-DB. Unknown structures are identified based on scores that reflect a matching and connection of the fragment spectra. Recently, the freely available software Skyline^416^ was furthermore used to identify fragments that are diagnostic for the discrimination of *N*- and *O*-glycan isomers.

Regardless whether used as a reference in publicly available databases or for structural identifications, the quality of the underlying raw data is absolutely crucial. This includes not only obvious measures in MS such as *m*/*z* resolution, mass accuracy, etc., but also less obvious metadata about data acquisition, processing, and storage. In order to ensure common quality standards in databases and publications, the MIRAGE (Minimum Information Required for a Glycomics Experiment) project, a community initiative coordinated by the Beilstein-Institute, was formed in 2011.^417^ Since then, a variety of specific, method-oriented guidelines—covering MS,^418^ LC,^419^ and bioinformatics^420^—have been established.

### 5.6 Fucose Migration

**Figure 25:**
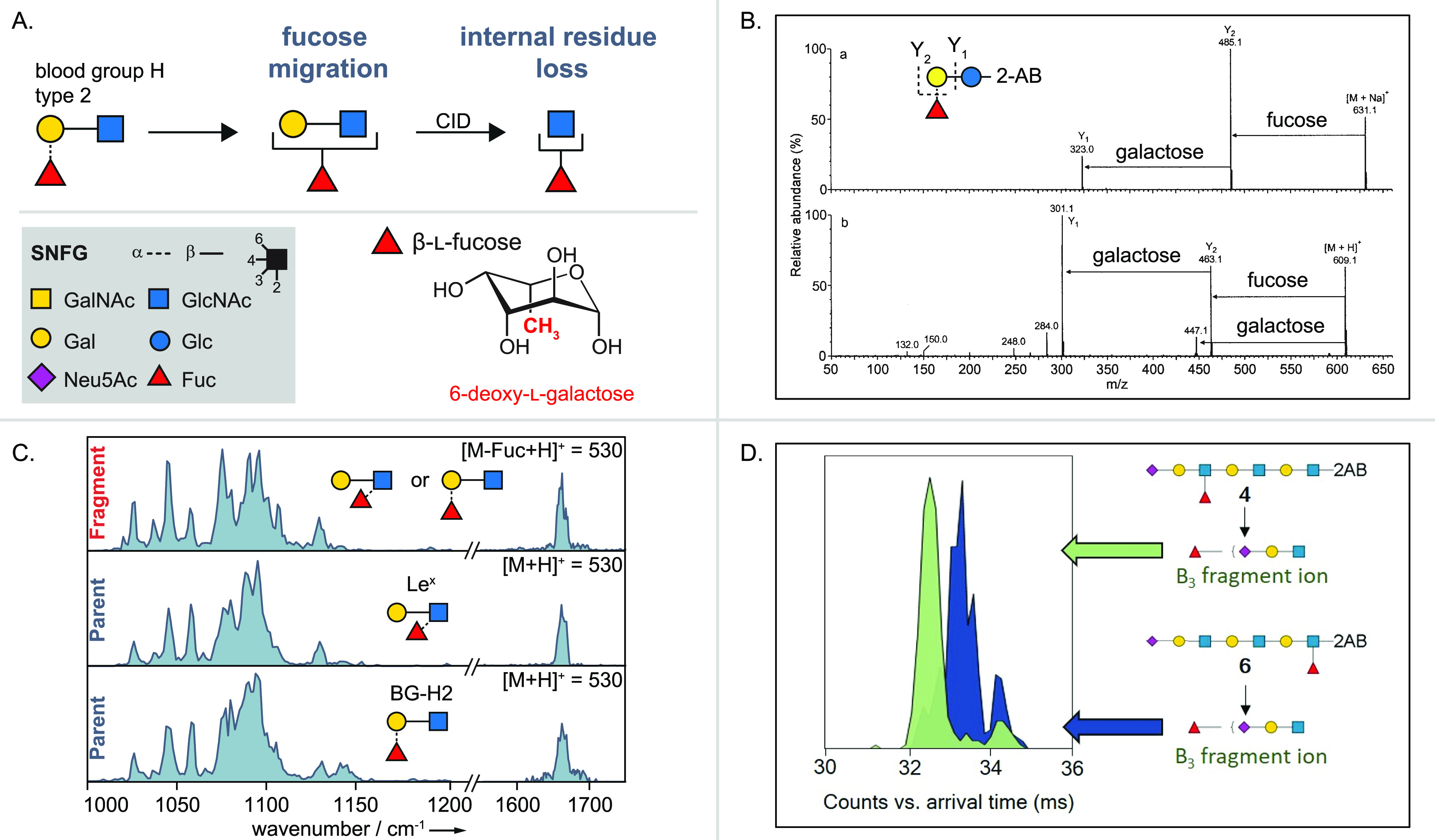
From early tandem MS experiments to IR spectroscopy and ion mobility experiments investigating fucose migration and internal residue loss. (A) Schematic example of fucose migration and internal residue loss for a trisaccharide (upper panel) in the symbol nomenclature for glycans (SNFG) (lower panel, left) and chemical representation of β-l-fucose, a 6-deoxy-l-galactose (lower panel, right). (B) Tandem MS experiments showing unexpected *m*/*z*-fragments from internal residue loss. Figure adapted with permission from ref (66). Copyright 2002 American Chemical Society. (C) IR spectroscopy experiments showing fucose migration in the trisaccharides Le^*x*^ and BG-H2 as in-source fragmentation products of a tetrasaccharide (upper panel) and as intact parent ions (middle and lower panel). Figure reprinted with permission from ref (205). Copyright 2018 Wiley-VCH. (D) IMS experiments distinguishing two fragments with identical *m*/*z* ratio but at least one with a rearranged fucose monosaccharide from their ATD. Figure reprinted with permission from ref (425). Copyright 2019 Wiley-VCH.

A reoccurring challenge in tandem MS of fucosylated glycans is fucose migration, an intramolecular rearrangement reaction, which can lead to erroneous sequence assignments (Figure 25).^421^ Owing to its importance in Lewis and blood group epitopes, fucose migration is discussed in the present section dedicated to *O*-glycans. However, this rearrangement reaction has been observed in various other glycan classes as well and may be regarded as a universal phenomenon in the MS-based analysis of fucosylated oligosaccharides. During migration, a fucose (6-deoxy-l-galactose) residue is transferred from the nonreducing end of a glycan to an adjacent or remote site within the same molecule. In fragmentation experiments using CID, fucose migration may be accompanied by internal residue loss (IRL). Unexpected *m*/*z* fragments indirectly indicate the occurrence of the reaction. The topic is of relevance not only for *O*-glycans but also for all classes of glycans that potentially contain fucose monosaccharides. Besides fucose, rearrangement reactions have been observed for xylose,^422,423^ rhamnose,^423,424^ and glucuronic acid,^423^ with distinct masses, but also mannose^328^ is able to migrate and more challenging to detect.

Several studies based on tandem MS, IMS,^425^ and IR spectroscopy^205,426^ have been published investigating the reaction mechanism and driving forces, the kinetics and energetics, and, of utmost concern, the destination of the migrating monosaccharide. The reaction is charge-induced, and therefore mobile protons or at least poor charge fixation is a necessity. The free hydroxyl groups within the oligosaccharides display a hydrogen bonding network of vicinal and *syn*-diaxial hydrogen bonds in which proton or charge transfer can take place.^426^ Both short-^66^ and long-range^427^ migration reactions are possible. For both cases, the close proximity in space of the migrating residue and the destination within the chain are important yet difficult to predict. The rearrangement reaction has been observed for 1,2-, 1,3-, 1,4-, and 1,6-linked migrating monosaccharides. Considering the time scale of the reaction, experiments using MALDI-TOF/TOF-MS show that the reaction for the investigated ions is faster than microseconds.^327^ The abundance of ion signals resulting from IRL is collision-energy-dependent. When plotting the relative abundance of an ion against the collision energy, the ion from a simple loss of a terminal fucose and the ion from IRL with migration of fucose show the same curve shape, indicating a similar mechanism.^66^

The exact mechanism of fucose migration remains unresolved to date, yet in the following, different approaches are reviewed. In oligosaccharides with a reducing-end modification of 2-aminobenzamide, the attack from the nitrogen in the linker with a transfer of the migrating residue and a subsequent glycosidic bond cleavage of the terminal residue has been proposed.^66^ The mechanism combines a migration reaction, which is independent of an internal loss, with a subsequent fragmentation. A similar reaction pathway but with migration to a remote hydroxyl group within the oligosaccharide has been suggested.^427^ Generally, functional groups other than the amine linker are plausible for the destination of migration since internal residue loss is observable in oligosaccharides with a methylated amine linker. In glycans containing sialic acids, it has been proposed^428^ that the oxygen of the amide group of a sialic acid attacks the anomeric center of fucose, and a new bond forms, leading to an imine group with the proton located at the reducing end of the chain.

In the case of rhamnose migration, a 6-deoxy-l-mannose, the following detailed mechanism has been suggested.^424^ The ring oxygen atom is protonated with subsequent cleavage of the adjacent C-1–O bond, resulting in a carbenium ion at the anomeric center of the migrating group. In 6-deoxy monosaccharides, the ring oxygen has a slightly higher proton affinity than in other monosaccharides. Then, the oxygen of the flavonoide residue linked to the reducing end of the diglycoside attacks the carbenium ion at the anomeric center, and a semirigid internal residue is eliminated from the oligosaccharide.

A very effective way to prevent the rearrangement reaction in mass spectrometry experiments is to measure metal adducts or deprotonated ions.^429^ Furthermore, reducing-end modifications such as procainamide labeling^430^ or a modification with a free radical precursor and a fixed charge on a pyridine moiety^271^ successfully suppress the reaction. All approaches aim at either demobilizing the proton at locations of high proton affinity or generally avoiding the presence of protons. Permethylation and peracetylation, on the other hand, do not in all cases prevent the rearrangement.^64,427,431^

## 6 Glycosaminoglycans

### 6.1 Structure and Analytical Challenges

Glycosaminoglycans (GAGs) are unbranched, highly acidic polysaccharides (kDa to MDa range), expressed by essentially all animal cells.^432^ Although also present intracellularly, GAGs are much more prominent on cell surfaces and in the extracellular matrix (ECM). They interact with a diverse set of soluble and membrane proteins, as well as ECM components: cytokines and chemokines, growth factors and their receptors, morphogens, blood coagulation factors, lipoproteins, integrins, and collagens.^433−437^ In addition to these endogenous binding partners, GAGs may also interact with viral and microbial proteins.^438,439^ Thus, they participate in various physio- and pathophysiological processes, such as embryonic and neural development, angiogenesis, hemostasis, inflammation, cancer progression, and infection.^13,14,440−444^

**Figure 26:**
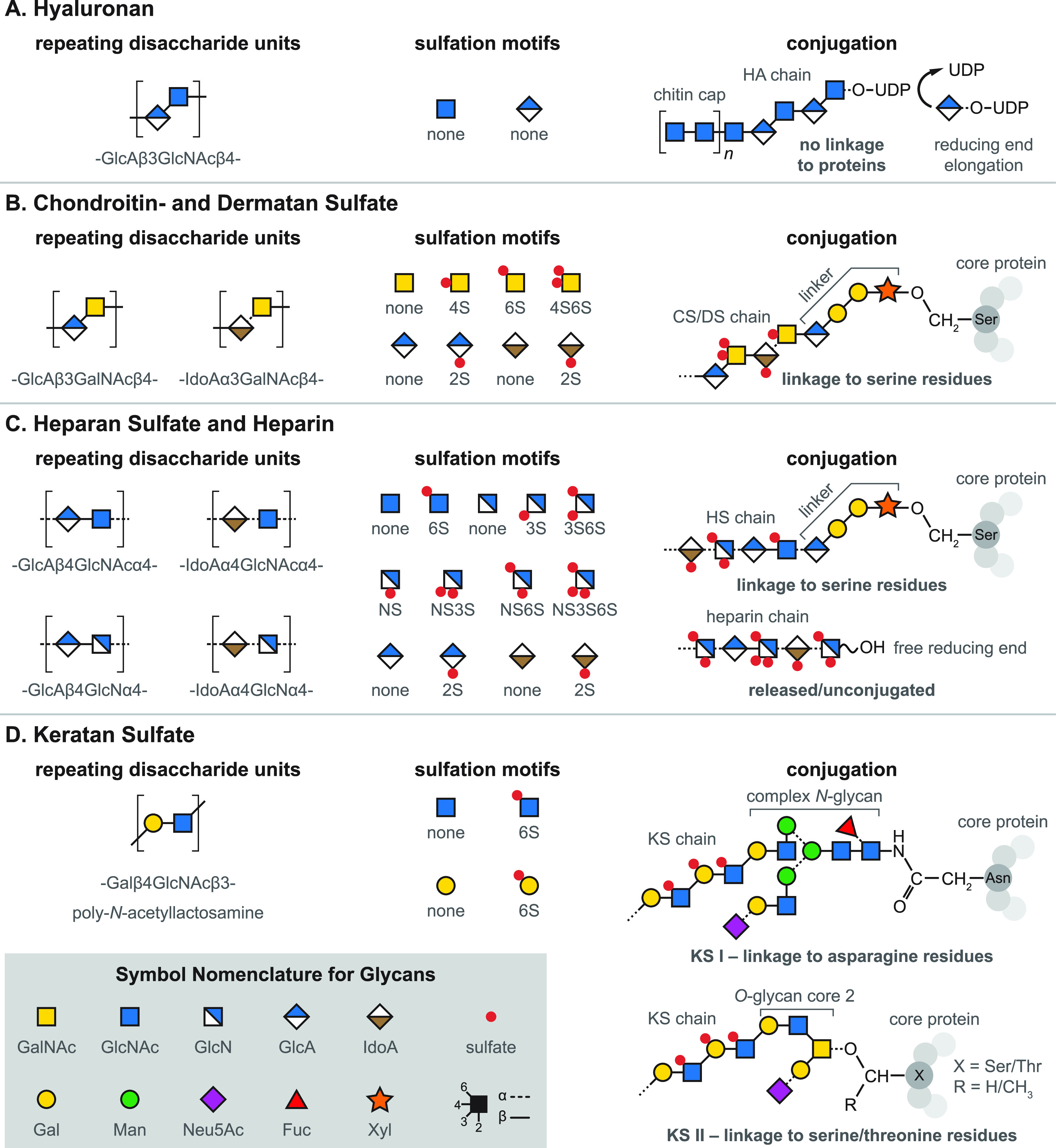
General structure of glycosaminoglycans. Overview of the characteristic disaccharide units, sulfation motifs, and potential protein linkages found in the four main glycosaminoglycan families. (A) Repetitive hyaluronan chains are not modified further by sulfation or epimerization. Uniquely, biosynthesis starts with the formation of a chitin cap and proceeds toward the reducing end. (B) Chondroitin and dermatan sulfate display a variety of sulfation motifs. The chains are linked to serine residues of proteoglycan core proteins through a specific tetrasaccharide linker. (C) Heparan sulfate and heparin represent the most diverse family of glycosaminoglycans. The heparin chain depicted corresponds to the antithrombin III binding sequence, mimicked by the synthetic anticoagulant fondaparinux. Discovery of additional sulfation motifs in the future cannot be ruled out. (D) Keratan sulfate contains galactose instead of hexuronic acid. The chains may be linked to both asparagine and serine/threonine residues of core proteins.

Albeit a structurally heterogeneous class of complex carbohydrates, some common, distinct features of GAGs render them a unique, well-recognizable group within the glycome. All GAGs possess a linear sequence formed by repeating disaccharide units, where an (occasionally deacetylated) *N*-acetylhexosamine alternates with hexuronic acids or galactose. Based on the structure of these disaccharides, four families of GAGs are distinguished traditionally: hyaluronan, chondroitin sulfate jointly with dermatan sulfate, heparan sulfate together with heparin, and keratan sulfate, as portrayed in Figure 26.^432,445^

Hyaluronan or hyaluronic acid (HA) is an extremely high molecular weight polysaccharide that consists of alternating *N*-acetyl-d-glucosamine (GlcNAc) and d-glucuronic acid (GlcA) residues, forming a poly(GlcAβ3-GlcNAcβ4) chain of several thousand disaccharide units (Figure 26A).^446^ HA emerged relatively late in the evolution of animals and appears to be an exception among GAGs in many aspects. It stands out as the only GAG where the repetitive copolymeric chain is not modified further by sulfation or epimerization. In addition, HA is not linked covalently to proteins, in stark contrast to other GAGs that form specific glycoproteins, so-called proteoglycans, whose functions are determined principally by their GAG constituents.

Chondroitin sulfate (CS) and dermatan sulfate (DS) are closely related galactosaminoglycans, often referred to as a single family and discussed in conjunction, owing to substantial similarities in structure, biosynthesis, and function (Figure 26B).^447^ The main difference between the two concerns their hexuronic acid residues. CS contains exclusively GlcA, alternating with *N*-acetyl-d-galactosamine (GalNAc) to build poly(GlcAβ3-GalNAcβ4) chains. In contrast, DS contains not only GlcA but also its C5 epimer, l-iduronic acid (IdoA), to varying extents. It leads to a more complex polymer backbone where two basic disaccharide building blocks vary across the sequence: (GlcAβ3-GalNAcβ4) and (IdoAα3-GalNAcβ4). It is, however, also popular to distinguish GlcA-containing CS disaccharides and IdoA-containing DS disaccharides categorically while referring to longer sequences containing both kinds of hexuronic acid as hybrid CS/DS chains. CS and DS chains may contain over 100 disaccharide units, and unlike HA, they are extensively sulfated. GlcA and IdoA may carry 2-*O*-sulfation, while GalNAc can be sulfated at the 4-*O* and 6-*O* positions. The resulting sulfation motifs give rise to several isomeric building blocks and a complex sulfation pattern across the chains that influences molecular recognition and may encode functional information.^448^ Typical GAG epitopes found within longer chains range from tetra- to decasaccharides (dp4–dp10). Elucidating the “sulfation code” of such bioactive protein-binding sequences is a major challenge in the analysis of all sulfated GAGs.

Heparan sulfate (HS) and heparin form the structurally most complex GAG family (Figure 26C). During their biosynthesis, the initial poly(GlcAβ4-GlcNAcα4) chain undergoes extensive modifications: epimerization of GlcA to IdoA, 2-*O*-sulfation of the hexuronic acids, *N*-deacetylation/*N*-sulfation, and 6-*O*- and the rare 3-*O*-sulfation of GlcNAc may all occur, affecting a varying number of residues.^449−451^ The result is a heterogeneous copolymer of enormous complexity, with extremely high density of ionizable functional groups.^452,453^ Like CS and DS, HS is linked to serine residues of specific core proteins via a xylose-containing tetrasaccharide. The sulfation and epimerization pattern of GAG chains in these proteoglycans show temporal and spatial variation across tissues. In general, the composition of HS chains found on a certain kind of proteoglycan, but in different cell types, shows higher variability than the chains found on different proteoglycans within the same cell. Although sharing the same set of disaccharide building blocks and basic sulfation motifs, important differences exist between HS and heparin.^434,454^ HS is expressed by virtually all animal cells, whereas heparin is produced by only a few cell types, most prominently connective tissue mastocytes. HS is attached to core proteins localized on cell surfaces and in the ECM. Heparin, on the other hand, is stored intracellularly in secretory granules, attached to its cytoplasmic core protein, serglycin. HS chains typically consist of 50–250 disaccharide units, while heparin chains are significantly shorter with an average molecular weight of 12–15 kDa. In HS, regions showing extensive sulfation and epimerization are clustered along the chain (NS domains), separated by largely unmodified regions (NA domains). Heparin lacks such domain structure, serving as a single extended NS region: most of its GlcA residues undergo epimerization to IdoA, and the chains are more heavily sulfated (around 2.5 sulfates per disaccharide) than in HS (roughly one sulfate per disaccharide on average). Heparin is the largest biopharmaceutical in production and widely used as an anticoagulant in unconjugated form.

Keratan sulfate (KS) is unique among GAGs as the chains lack hexuronic acid, containing instead d-galactose (Gal).^455,456^ In KS chains, up to 50 repeating (GalAβ4–GlcNAcβ3) disaccharide units form the linear poly-*N*-acetyllactosamine backbone, whose strong acidic character stems from sulfation. Sulfate groups can be installed at the 6-*O*-position of both Gal and GlcNAc residues, with sulfated Gal occurring mainly adjacent to sulfated GlcNAc. In addition to *O*-mannose (KS III, not shown) and *O*-GalNAc (KS II) linkages, the chains may also be linked to asparagine residues (KS I) of core proteins in KS proteoglycans. Although the complex-type *N*-glycan linker may possess multiple antennae, KS chains themselves are not branched (Figure 26D).

In general, sulfated GAGs represent an immense structural complexity and are among the most challenging biopolymers to characterize. Obtaining information on the sequence of even the simplest full-length chains is a formidable task.^457−459^ Complexity and the associated challenges stem from four chief aspects of GAG structure: high degree of polymerization combined with size polydispersity, sequence microheterogeneity, high negative charge density, and the potentially isomeric building blocks. Being highly polydisperse, the length of GAG chains found on a certain proteoglycan at a given position is not uniform. Because of their microheterogeneity, GAGs cannot be characterized by a single, well-defined sequence, in contrast to biopolymers with template-driven biosynthesis, such as proteins or coding DNA. The dense sulfation of GAGs complicates their MS analysis due to Coulomb repulsion, sulfate loss, and the formation of multiple adducts. Finally, epimerization and sulfation at various positions lead to a large number of isomeric building blocks, difficult to distinguish by MS-based methods relying ultimately on the measurement of *m*/*z* ratios.

**Figure 27:**
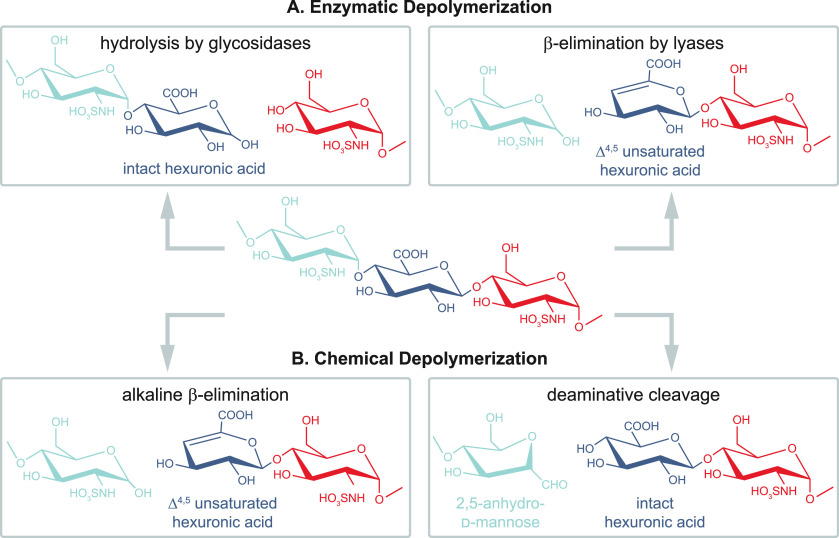
Common glycosaminoglycan depolymerization strategies shown through the example of heparan sulfate/heparin. (A, left) Enzymatic depolymerization of GAG chains may be performed using glycosidases, resulting in hydrolytic cleavage that preserves the hexuronic acid stereochemistry. To obtain oligosaccharide fragments covering the full sequence, enzymes with endolytic activity are necessary. Heparanases are *endo*-β-glucuronidases cleaving at the reducing end of GlcA residues in moderately sulfated HS/heparin chains. (A, right) Prokaryotic lyases, such as heparinase I–III, act via a β-eliminative mechanism, leading to Δ^4,5^-unsaturated uronic acid residues at the new nonreducing end. Consequently, stereochemical information is lost in the process. (B, left) Benzyl esterification with alkaline β-elimination may be applied for the depolymerization of GAGs, mimicking lyase activity. (B, right) Deaminative cleavage preserves hexuronic acid stereochemical information at the cleavage site but alters the structure of the glucosamine through the formation of 2,5-anhydromannose. The reaction is blocked in the presence of *N*-acetyl groups on glucosamines, making prior deacetylation necessary.

Due to the sheer size of full-length GAG polysaccharides, (partial) enzymatic or chemical depolymerization of the chains (Figure 27) is crucial for obtaining smaller oligosaccharides tractable by state-of-the-art MS methods.^453,460−462^ Thus, strategies to characterize and sequence GAGs tend to follow a bottom-up approach. The complexity of the mixture resulting from depolymerization, along with the inherent polydispersity and sequence microheterogeneity, imply that extensive multistep separations are indispensable in GAG analysis, as addressed in excellent reviews.^453,463−465^ Depolymerization, combined with chromatographic and electrophoretic separations, provides the link between the full-length GAG chains and the shorter oligosaccharides (dp < 12) compatible with MS analysis. Instead of focusing on sample preparation, condensed-phase separations, or disaccharide profiling, herein we review developments in the methodology and instrumentation of MS-based techniques in the context of oligosaccharide analysis and sequencing. These developments include novel ion activation methods, the hyphenation of IMS to MS, and action spectroscopy of mass-selected GAG ions. In the past 15 years, the above inventions significantly increased the amount and specificity of structural information obtainable on GAGs at the oligosaccharide level. They successfully tackle challenges arising from dense sulfation and isomerism, two aspects of GAGs that have impeded their analysis using traditional MS techniques.

### 6.2 Electron-Based Dissociation Methods in GAG Analysis

**Figure 28:**
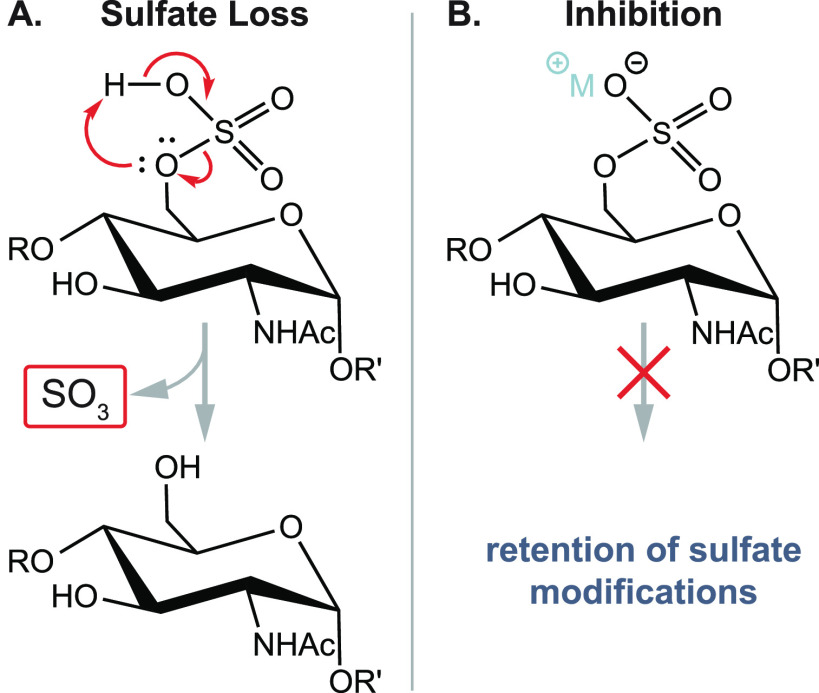
Sulfate equivalent loss of glycosaminoglycans in the gas phase. (A) Sulfate loss in the form of neutral SO_3_ upon ion heating. Protonated sites are more prone to undergo decomposition. (B) Deprotonation of sulfate groups inhibits the undesirable reaction. Quaternary ammonium or metal ions (M^+^) may neutralize the charge of deprotonated sulfate groups, reducing intramolecular Coulomb repulsion and facilitating the removal of protons.

Owing to their acidity, GAGs have mainly been analyzed in negative ion mode by ESI-MS. They exhibit high ionization efficiencies, with sulfates and—to a lesser extent—carboxylates carrying the negative charges.^465^ Mass spectra of GAGs are highly complex, as even a single, well-defined oligosaccharide can give rise to a multitude of ions: various charge states may be simultaneously present, each with a distribution of adducts due to H^+^/metal ion exchange. A major challenge in the MS analysis of highly sulfated species is the undesirable decomposition of sulfate modifications, appearing in the form of SO_3_ neutral loss (79.96 Da).^466^ Sulfate loss hampers compositional analysis and the localization of sulfate modifications. This unimolecular reaction has a low activation barrier in the gas phase and affects protonated sulfate groups. Removal of the proton from sulfates successfully inhibits the process and the resulting loss of information, as depicted in Figure 28. Exchanging the neutralizing proton to an aprotic tetraalkylammonium/metal cation is a common strategy to reduce intramolecular Coulomb repulsion between charged sulfate groups, thereby facilitating deprotonation in densely sulfated species. Although ESI is among the softest ionization methods, sulfate loss products often occur as in-source fragments, even without additional ion activation. Therefore, applying the softest possible source conditions is essential when analyzing highly sulfated compounds. As slow-heating methods favor dissociation channels with the lowest barriers, sulfate loss products dominate CID and IRMPD product ion spectra. Both of these traditional dissociation methods yield abundant glycosidic bond cleavages, while cross-ring fragments—often crucial for the precise localization of sulfate modifications—are scarce.^465^ Various attempts were made to overcome these shortcomings, facilitate cross-ring cleavages, and reduce sulfate losses in CID mass spectra. Although metal ion adduction,^467−469^ derivatization,^470−472^ or positive ion mode analysis^473^ proved to be successful in many aspects, inherent characteristics of slow-heating methods limit their utility for GAG analysis.

**Figure 29:**
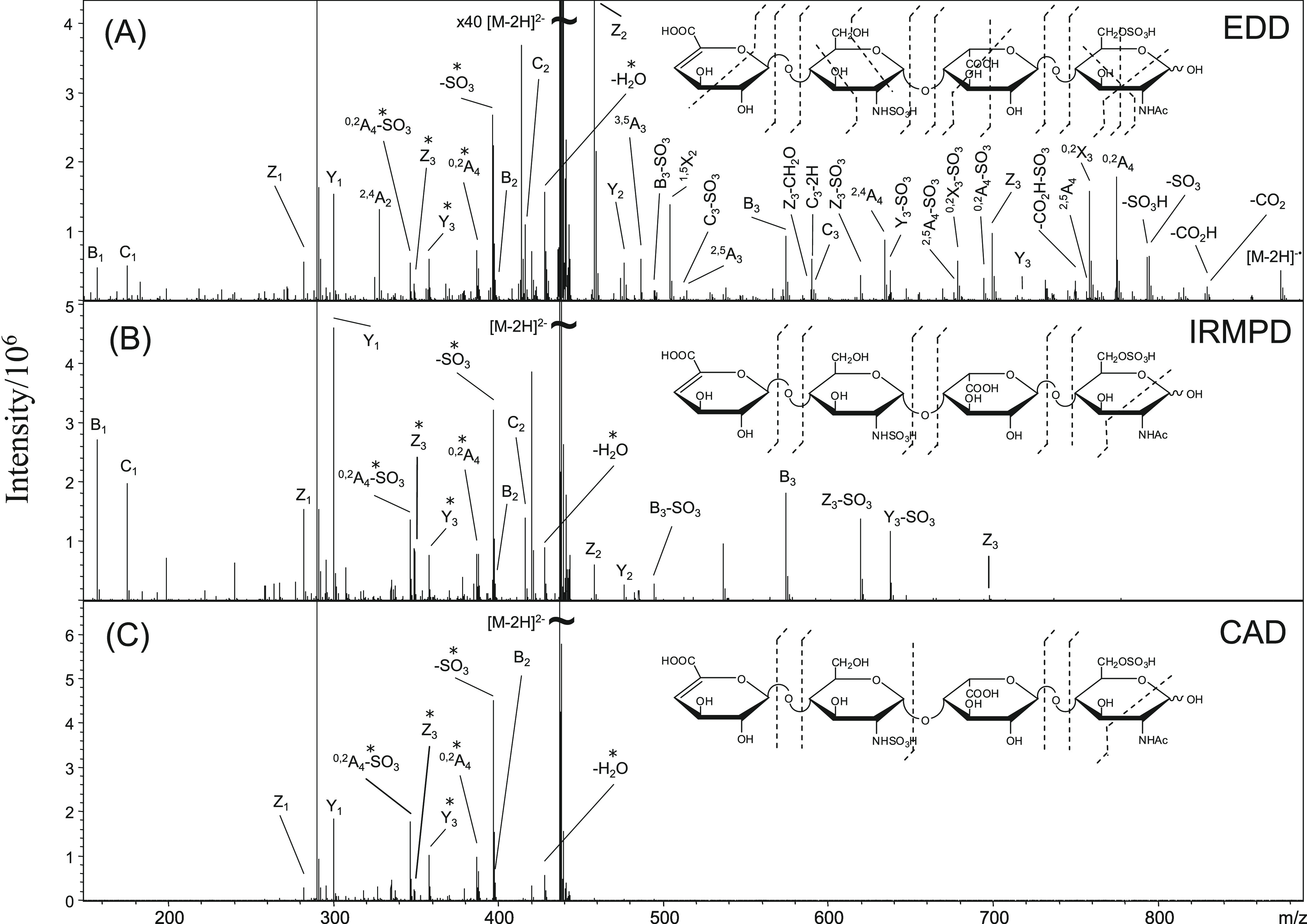
Electron detachment dissociation (EDD) vs slow-heating fragmentation of glycosaminoglycans. (A) EDD tandem mass spectrum and corresponding fragmentation pattern of a synthetic heparan sulfate tetrasaccharide as [M – 2H]^2–^. Tandem mass spectra and fragmentation patterns resulting from (B) infrared multiple photon dissociation (IRMPD) and (C) collision-induced dissociation (CID) of the same precursor ion. Doubly charged product ions are indicated with an asterisk. Figure reprinted with permission from ref (86). Copyright 2007 American Society for Mass Spectrometry.

In a landmark study, Wolff et al. employed EDD for the first time for GAG analysis, comparing the EDD fragmentation pattern of four modestly sulfated HS tetrasaccharide dianions to those obtained by CID and IRMPD.^86^ EDD may be regarded as the negative ion counterpart of ECD, a nonergodic fragmentation process developed for polycations.^79^ In EDD, multiply charged anions are irradiated by electrons of moderate kinetic energy (15–20 eV), causing electronic excitation and electron detachment, initiating radical-driven dissociation pathways. EDD product ion spectra of the HS tetrasaccharides were informative, highly reproducible, and rich in A- and X-type cross-ring fragments; yielded a full set of glycosidic cleavages for all analytes; and exhibited much more favorable sulfate retention than CID or IRMPD (see Figure 29). Thus, this fragmentation technique overcame many challenges associated with the tandem MS analysis of GAG oligosaccharides. A drawback of EDD is the dependency on expensive FTICR-MS platforms, as trapping molecular ions and electrons simultaneously in the presence of radiofrequency (RF) fields, such as in linear ion traps and Paul traps, is not straightforward.^474−479^ In addition, electrostatic repulsion between polyanions and electrons of moderate kinetic energy makes the process rather inefficient, with consequently long interaction periods required.

The capabilities of EDD were further explored for the analysis of more heavily sulfated HS species^480^ and for larger DS oligosaccharides (dp4–dp10).^481^ A systematic analysis concerning the influence of charge state and cation adduction on the EDD fragmentation pattern of DS oligosaccharides revealed that electron detachment from carboxylates is preferred over sulfates for thermodynamic reasons, provided the carboxylates are not protonated.^482^ Deprotonation of carboxyl groups increases sulfate retention upon EDD but requires high precursor charge states or cation adduction due to the higher proton affinity of carboxylates over sulfates.

Another fruitful application of EDD concerns the stereochemistry of hexuronic acid residues. Distinguishing GlcA from its C5 epimer IdoA in HS/heparin or hybrid CS/DS chains is an important task in GAG research.^483^ However, it is extremely difficult to accomplish by MS methods that ultimately reduce structural information to *m*/*z* ratios. In a study by the Amster group, pairs of diastereomeric HS-related tetrasaccharides were used as model compounds: each species carried GlcA or IdoA in the second position and a Δ^4,5^ unsaturated uronic acid residue at the nonreducing end. EDD provided diagnostic fragments, enabling the distinction of GlcA-containing oligosaccharides from their counterparts carrying IdoA. According to the proposed hypothesis, the formation of these fragments involves hydrogen atom transfer to the carboxyl radical from neighboring carbons or hydroxyl groups. The site of hydrogen abstraction, which will determine subsequent reaction steps and the nature of fragment ions formed, depends on key interatomic distances and thereby on the configuration of C5. In less unambiguous cases, when no diagnostic fragments could be identified, EDD was combined with principal component analysis (PCA). Multivariate statistical analysis facilitated both the distinction of diastereomeric HS and CS/DS tetrasaccharides and the stereochemical assignment of certain hexuronic acid residues.^484−487^ The possibilities of assigning the configuration of hexuronic acid units farther away from the reducing end, or determining the stereochemistry of multiple residues simultaneously in longer chains, are intriguing questions that may inspire future research.

Following the introduction of EDD to the field, the family of ExD methods applied for GAG oligosaccharides has been extended by EID^266^ and NETD.^89^ EID is performed by irradiating singly charged anions with electrons of moderate kinetic energy (6–20 eV), inducing electronic excitation. Electron detachment from monoanions leads to neutral species, invisible by MS. Similarly to EDD, EID produces both even- and odd-electron fragment ions and cross-ring cleavages that affect primarily hexuronic acid residues.^266^ However, it seems to be less sensitive to hexuronic acid stereochemistry and, lacking clear advantages over EDD, has not gained widespread popularity in the field. NETD, in contrast, earned popularity in GAG research, owing to its compatibility with a variety of MS platforms, the rich fragmentation patterns, and the short interaction periods it requires for efficient fragmentation.^488^ NETD represents the negative ion mode counterpart of ETD: instead of transferring electrons to positive analytes with radical anions as in the latter, NETD utilizes radical cations to abstract electrons from polyanions, initiating radical-driven dissociation pathways.^489^ Common sources of the reactive radical cations are fluoranthene and Xe, but other gases may also be applied.^465^ Although NETD is based on ion–ion reactions and not on direct ion–electron interactions, the resulting dissociation pathways and fragmentation patterns closely resemble those observed in other ExD techniques, justifying the inclusion of NETD into this family of ion activation methods.

Huang and Yu et al. compared NETD and EDD on an FTICR-MS platform, using highly sulfated HS/heparin model oligosaccharides up to dp6.^490^ Despite remarkable advances in the chemical,^491−499^ enzymatic,^500,501^ and chemoenzymatic^502−508^ synthesis of GAGs, the accessibility of standards with well-defined structures is not comparable to that found in the fields of peptide and nucleic acid research. One of the few commercially available standards is fondaparinux (Arixtra), a fully synthetic anticoagulant mimicking the antithrombin III binding sequence of heparin. This pentasaccharide, carrying 8 sulfate modifications, showed comparable fragmentation patterns in EDD and NETD, with a full set of glycosidic cleavages and plentiful cross-ring fragments, enabling MS-based sequence assignment. In general, NETD proved to be a more efficient dissociation process for highly charged species, while EDD was better suited for the analysis of dianions. Interestingly, NETD led to fewer sulfate loss products, both in number and in abundance. This phenomenon may be related to the absence of direct electronic excitation when using cation radicals instead of ∼20 eV electrons.

**Figure 30:**
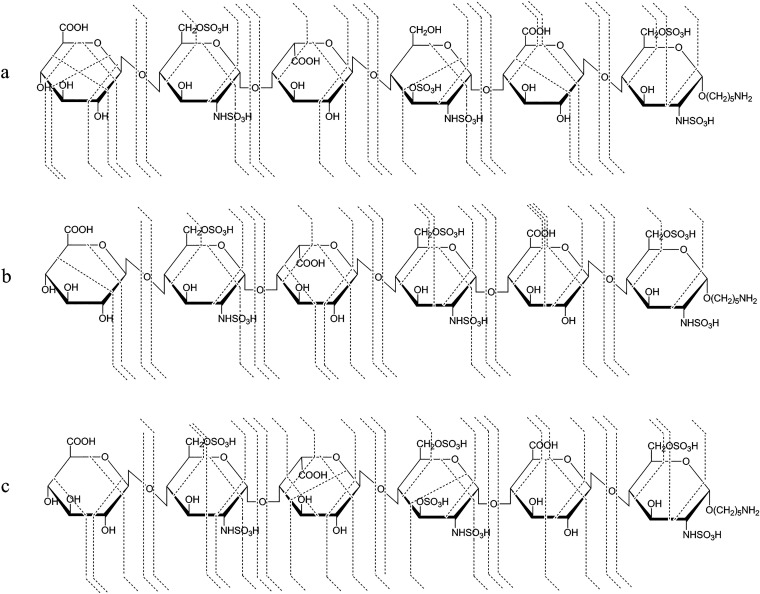
Negative electron transfer dissociation (NETD) fragmentation patterns of synthetic heparin/heparan sulfate hexasaccharides. (a) GlcA-GlcNS6S-IdoA-GlcNS3S-GlcAGlcNS6S; (b) GlcA-GlcNS6S-IdoA-GlcNS6S-GlcA-GlcNS6S; and (c) GlcA-GlcNS6S-IdoA-GlcNS3S6S-GlcA-GlcNS6S. The highly sulfated glycans were measured as [M – H]^5–^ polyanions. Note the diagnostic cross-ring fragments enabling the localization of sulfate groups within the glucosamine residues. Reprinted with permission from ref (509). Copyright 2018 American Society for Mass Spectrometry.

Zaia and co-workers systematically studied the capabilities of NETD to distinguish the rare 3-*O*-sulfation from the common 6-*O*-sulfate modification in HS/heparin oligosaccharides.^509^ Previously, NETD did not allow for unambiguous localization of sulfate modifications (4-*O*- vs 6-*O*-sulfation) in a CS oligosaccharide, due to the lack of diagnostic product ions, e.g., cross-ring fragments with a bond cleavage between C4 and C5 of GalNAc residues.^510^ In contrast, diagnostic fragments were generated by NETD for 3-*O*-sulfated HS species, enabling the assignment of 3-*O*- vs 6-*O*-sulfation position in GlcNS units, as highlighted in Figure 30.

Taking advantage of its short interaction periods, MS with NETD has been successfully coupled in an online fashion to HILIC by Wu and co-workers.^90^ A library of 15 HS oligosaccharide standards, containing di- and tetrasaccharides with varying degrees of sulfation, including many isomeric species, was used to demonstrate the potential of the workflow for sequencing. Although NETD fragment ion spectra alone did not enable the assignment of hexuronic acid stereochemistry, the diastereomers could be successfully separated using HILIC. Strong structure–retention relationships were revealed, with IdoA-containing chains eluting earlier than their GlcA-containing counterparts. In accordance with previous results, 3-*O*- and 6-*O*-sulfation motifs could be unambiguously assigned by NETD MS/MS on the chromatographic time scale. Similarly to the HILIC coupling, hyphenation of NETD MS/MS to capillary zone electrophoresis (CZE) has been recently accomplished.^511^ The 50–100 ms interaction periods required for efficient NETD of GAGs enabled sufficiently frequent sampling of the narrow (30 s) CZE peaks. Although hexuronic acid isomers could be separated by CZE, their identification based on the NETD fragmentation patterns remains an unresolved challenge.

Finally, in relation to ExD techniques, it is worth mentioning charge-transfer dissociation (CTD), an activation method recently demonstrated to yield informative fragment ion spectra for HS, CS, and DS oligosaccharides.^512^ In CTD, ions are collided with high-energy (several keV) He^+^ ions. As He has an exceptionally high ionization energy (24.6 eV), He^+^ ions abstract electrons from the analytes. Electron transfer is accompanied by rapid excitation of the oxidized radical product ion, promoting radical-driven dissociation pathways. Like NETD, CTD is based on ion–ion reactions and may be readily implemented into various MS platforms. Although providing abundant cross-ring cleavages in general, CTD did not deliver diagnostic fragments, enabling the unambiguous assignment of sulfate positions (4-*O* vs 6-*O*) in GalNAc residues of CS/DS oligosaccharides. This intriguing aspect of GAG structural diversity represents an important challenge in the field, likely to inspire future research and developments.

### 6.3 Ultraviolet Photodissociation Mass Spectrometry of GAGs

Although not as widespread in GAG analysis as the various ExD methods, UVPD MS has become increasingly popular in recent years and is expected to further expand in the near future, owing to the commercialization of the technique. An advantage of UVPD is its compatibility with linear and 3D quadrupole ion traps,^32^ opposed to EDD that is difficult to implement in the above-mentioned platforms and is mainly performed using expensive FTICR-MS (Penning traps). To date, UVPD on GAGs has been performed exclusively in negative ion mode. In general, UVPD fragmentation patterns of GAG anions are characterized by (1) retention of sulfate modifications to an extent comparable to that observed in ExD methods, (2) extensive glycosidic bond cleavages, resulting in good sequence coverage, and (3) abundant cross-ring fragments that help verify backbone linkage positions, localize sulfate modifications, and distinguish isomers. Many of these favorable characteristics stem from the fact that absorption of energetic (>3 eV) UV photons leads to fast energy deposition and electronic excitation, in contrast to the gradual vibrational excitation in slow-heating CID and IRMPD. Direct dissociation from excited electronic states is not the only fragmentation form associated with the UV photoexcitation of isolated GAG anions. EPD from multiply charged ions enables radical-driven dissociation pathways, while internal conversion in combination with IVR leads to thermal fragments, similar to those observed upon CID. The capabilities and potential of UVPD MS in GAG analysis are demonstrated through selected works below.

A series of pioneering experimental and theoretical works on UVPD MS of GAGs were carried out by Racaud et al. In a proof-of-principle study, UVPD was performed in a linear ion trap using a tunable optical parametric oscillator (OPO) laser between 220 and 290 nm (4.3 to 5.6 eV).^513^ By plotting the fragmentation yield as a function of the irradiation wavelength, the authors recorded gas-phase UV action spectra of oligosaccharide ions for the first time. One nonsulfated and two singly sulfated HS/heparin-derived disaccharides served as model compounds, investigated as singly deprotonated ions. Fragmentation yield increased linearly with the laser power, indicating that UVPD of the investigated species is a single-photon process. UVPD fragmentation patterns were recorded at 240 nm, the absorption maximum of the sulfated species, and compared to those obtained by CID. In general, UVPD led to more abundant cross-ring cleavages that helped localize sulfate modifications. Some of these X- and A-type fragments were specific to UVPD and likely result from direct dissociation from excited electronic states. The unspecific UVPD fragments observed also upon CID, on the other hand, were probably generated following internal conversion and the redistribution of vibrational energy. As the ions carried at maximum one deprotonated sulfate group, SO_3_ loss channels were not dominant.

In a follow-up publication, the strategy of inducing fragmentation of HS/heparin-derived anions by UV photoexcitation was further expanded.^514^ At 220 nm irradiation wavelength, two competing processes were observed for di- and tetrasaccharide dianions: UVPD and EPD. Electron detachment led to charge-reduced, oxidized radical species that were further subjected to CID. The resulting hybrid dissociation method that merges UV irradiation with collisional activation was previously termed activated (a-)EPD by the authors. By opening up new, radical-driven dissociation pathways in GAG anions, a-EPD gave rise to informative fragments distinct from those observed upon UVPD. In addition, the relative contributions of UVPD and EPD could be systematically tuned by Na^+^ adduction. While EPD was suppressed when all carboxyl groups were protonated, it became more dominant when the proton on one or more carboxyls was exchanged to Na^+^, leaving the precursor ion charge state unaltered. Thus, EPD in GAG ions is strongly linked to the presence of deprotonated carboxylates.

**Figure 31:**
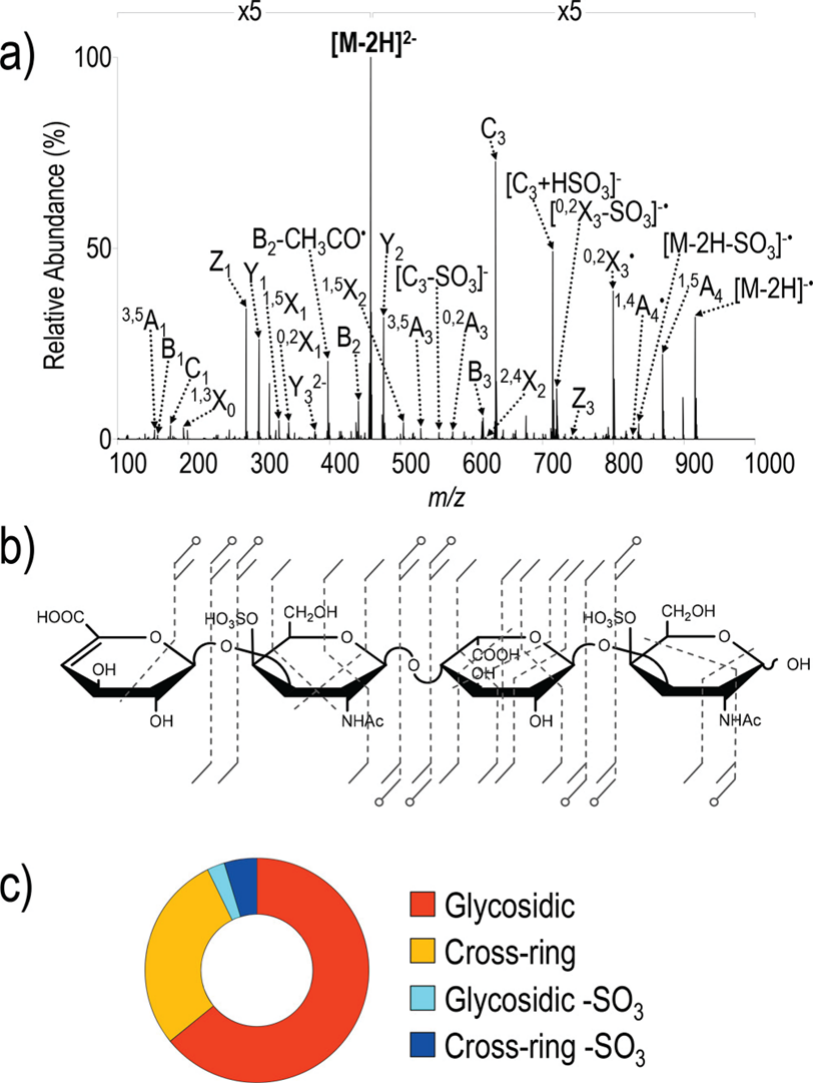
Ultraviolet photodissociation (UVPD) tandem MS of a dermatan sulfate tetrasaccharide at 193 nm. (a) Product ion spectrum of the doubly deprotonated oligosaccharide (*m*/*z* 458). (b) The corresponding UVPD fragmentation pattern and structural assignment. (c) Donut plot depicting the distribution of photofragments based on summed abundances of fragment types. Reproduced with permission from ref (515). Copyright 2019 American Chemical Society.

In a joint study by the Amster and Brodbelt groups, the fragmentation of various GAG oligosaccharide anions was investigated in UVPD experiments, employing a 193 nm ArF excimer laser on a Fourier transform mass spectrometry (FTMS) platform.^515^ UVPD was compared to high-energy collisional dissociation (HCD), EDD, and NETD. The model compounds included moderately sulfated HS and DS oligosaccharides (dp4 and dp10) and the highly sulfated pentasaccharide fondaparinux. HCD generated mainly glycosidic fragments and abundant sulfate loss products. In contrast, EDD, NETD, and UVPD led to more informative product ion spectra with abundant sulfate-retaining fragments, including cross-ring cleavage products. Besides providing a complete set of glycosidic cleavages and thus full sequence coverage for all model species, UVPD exhibited the highest abundance of cross-ring fragments. Many of these informative fragments were observed exclusively upon UVPD, including diagnostic ^2,4^X- and ^1,4^A-type fragments enabling unambiguous assignment of 4-*O*-sulfation in a DS tetrasaccharide, highlighted in Figure 31. To the best of our knowledge, such an achievement was not reported previously for ExD methods. UVPD experiments also revealed charge-reduced EPD products and the dependence of fragmentation patterns on precursor ion charge state and cation adduction. Moreover, the data indicated occasional sulfate transfer between monosaccharide units, a form of mass spectrometric rearrangement worthy of further investigation. Finally, the authors assessed the capabilities of UVPD using a more user-friendly solid-state Nd:YAG laser at 213 nm. The fragmentation pattern of the model DS tetrasaccharide at 213 nm was similar to that observed employing the 193 nm excimer laser and the same MS instrument, with discrepancies likely stemming from the difference in photon energies (5.8 vs 6.4 eV). However, an order of magnitude longer irradiation periods were needed at 213 nm, due to decreased fragmentation efficiency caused by the significantly lower pulse energy of the solid-state laser. This observation underlines the importance of bright UV sources, necessary to achieve sufficiently intense fragment ion peaks in UVPD MS within an interaction period comparable to that in NETD.

Building on the above findings, the authors systematically studied the influence of key experimental parameters on the fragmentation of CS and DS tetrasaccharides upon UVPD.^516^ Importantly, the ionization state of the precursor anions appeared to have a greater impact on the fragmentation patterns than the photon energy or the number of laser pulses employed. Upon choosing the suitable precursor ions, where the number of deprotonated groups either equaled the number of sulfates or exceeded it by one, UVPD at both 193 and 213 nm led to significant sulfate retention and enabled the assignment of 4-*O*-sulfation by generating diagnostic ^1,4^A-, ^2,4^A-, and ^2,4^X-type fragments.

Recently, UVPD MS was performed on fondaparinux ions in negative ion mode, using a commercial FTMS setup equipped with a 213 nm solid-state Nd:YAG laser.^517^ In the product ion spectrum of the doubly deprotonated fondaparinux precursor, a Y_3_/C_3_ internal glycosidic fragment was observed at *m*/*z* 417.94. This [GlcNS3S6S – H]^−^ ion corresponds to the monosaccharide residue located in the middle of the pentasaccharide sequence, carrying the rare 3-*O*-sulfate modification that is found in some specific protein binding sequences. The characteristic triply sulfated monosaccharide residue is essential for antithrombin III binding and the resulting anticoagulant effect of heparin and related pharmaceuticals, but little is known about its other biological functions. Searching for exotic structural elements in the hope of finding specific protein binding sites along the glycan chain is an important aspect of GAG research.^518^ Based on their results, the authors envisaged an LC-MS/MS screening method using UVPD to detect HS/heparin sequences containing the rare GlcNS3S6S motif. Although such a method would have far-reaching applications in biomedical research and drug discovery, it is yet to be clarified whether the [GlcNS3S6S – H]^−^ fragment ion is specific to the doubly deprotonated fondaparinux or appears more generally among the UVPD fragments of HS/heparin oligosaccharides carrying the GlcNS3S6S motif, thereby enabling the proposed screening strategy.

### 6.4 Ion Mobility–Mass Spectrometry in GAG Analysis

Since the commercialization of the first IM-MS instruments, gas-phase electrophoretic separations have contributed significantly to GAG sequencing approaches. They proved to be especially useful for separating GlcA- vs IdoA-containing stereoisomers, complementing tandem MS approaches. Employing a TWIM-MS instrument, Leary and co-workers demonstrated the separation of two HS hexasaccharide epimers as dianions.^519^ Although CCS values were not reported, the separation of stereoisomers differing merely in the configuration of a single carbon clearly showed the potential of IMS in GAG analysis. In a related systematic study, six synthetic HS octasaccharide stereoisomers—containing GlcA/IdoA residues in different ratios and positions—were investigated as multiply deprotonated species by TWIM-MS using He buffer gas.^520^^TW^CCS_He_ values were determined using a calibration curve based on a set of oligonucleotide standards. Oligosaccharides containing IdoA at the reducing end had systematically higher CCS values than their GlcA-containing counterparts. Although extraordinary care must be taken when comparing solution and gas-phase glycan conformations, it is reasonable to assume that IdoA and GlcA residues adopt different ring puckers not only in solution but also in the gas phase, which provides a rationale for the observed sequence–CCS correlation.

Multiple studies demonstrated the separation of sulfation positional isomers for HS^521−523^ and CS/DS species.^524^ Interestingly, the separation of the isomeric CS/DS disaccharides ΔUA-GalNAc4S and ΔUA-GalNAc6S in N_2_ buffer gas was more efficient when analyzing them as triply sodiated, singly charged cations. As the mobilities of the isomers were very close to each other, baseline resolution required outstanding resolving power, provided by an atmospheric pressure DTIM-MS instrument.

An important aspect of coupling IMS to MS, especially to tandem MS, is the compatibility of time scales. FTICR-MS is a relatively slow mass analyzer, while ExD methods highly suited for GAG analysis often require interaction periods on the order of 100 ms, rendering comprehensive, nested IM-MS^2^ experiments unfeasible. Strategies to overcome this difficulty include the application of filtering IMS devices, such as FAIMS, or the storage of mobility-selected ions for longer time periods before fragmentation and mass analysis. Kailemia et al. coupled FAIMS to FTICR-MS, which enabled the postionization separation of epimeric HS tetrasaccharides in combination with their selective MS/MS analysis employing EDD.^163^ FAIMS also separated members of a CS dp4–dp10 homologue series, where the species differed in both mass and charge but yielded overlapping peaks on the *m*/*z* scale. This charge state separation underlines the advantage of IMS as an additional, orthogonal separation step that reduces stress on subsequent MS analysis, resulting in less congested mass spectra.

**Figure 32:**
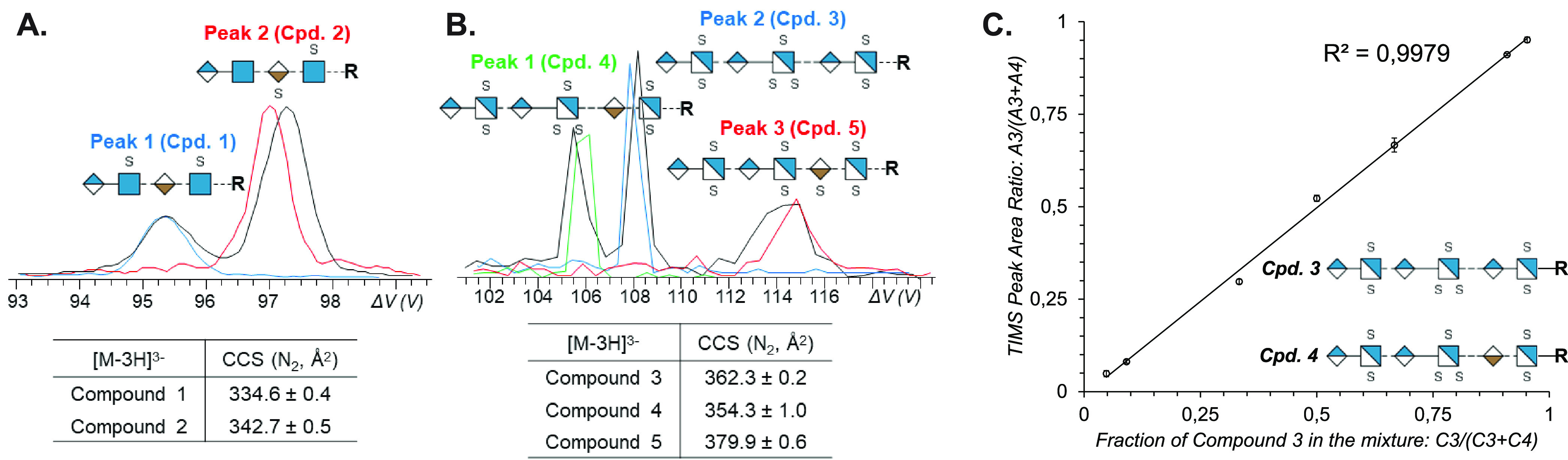
Gated trapped ion mobility separation of heparin/heparan sulfate oligosaccharides. (A) Arrival time distributions (ATDs) of two isomeric tetrasaccharides (blue and red traces) and that of their mixture (black trace). (B) ATDs of three highly sulfated hexasaccharide isomers (blue, green, and red traces) and that of their mixture (black trace). Collision cross sections of each compound (measured as triply deprotonated species) are listed below. R stands for an aminopentyl linker. (C) Relative quantification of the two hexasaccharide stereoisomers (compounds 3 and 4) enabled by trapped ion mobility spectrometry. The peak area ratio, A3/(A3 + A4), was averaged over three technical replicates and plotted against the ratio of the concentration of compound 3 over the total concentration, C3/(C3 + C4). Error bars represent the standard deviations of three measurements. Figure adapted with permission from ref (525). Copyright 2019 American Chemical Society.

Lin and co-workers combined TIMS with NETD MS/MS on an FTICR-MS instrument, analyzing a set of highly sulfated synthetic HS/heparin oligosaccharides (dp4–dp6).^525^ As TIM separations and NETD MS/MS take place on comparable time scales, their combination requires decoupling fragmentation and *m*/*z* analysis from the preceding mobility separation. This was achieved using a gated-TIMS strategy.^526^ Mobility- and *m*/*z*-selected ions emerging from the TIMS funnel and the downstream quadrupole mass filter were first trapped in a hexapole storage cell, which enabled the accumulation of ions from multiple TIMS cycles. Then, following multiple rounds of storage cell filling and potential NETD in the cell, the ions were released into the high-resolution mass analyzer. FTMS analysis and ion accumulation in the hexapole cell can be performed in parallel, increasing the duty cycle of the workflow. In contrast to prolonged residence times in TIMS funnels that may lead to significant rf heating, extensive storage times in the hexapole cell were not accompanied by notable ion activation: sulfate losses were negligible even after several hundred filling cycles. Sulfation position isomers and epimers differing only in one hexuronic acid residue could be successfully separated in N_2_ buffer gas, and ^TIMS^CCS_N2_ values were obtained using perfluoroalkyl phosphazine ions to establish the calibration curves. Baseline resolution of two hexasaccharide epimers by TIMS allowed for their relative quantification (Figure 32), while NETD in combination with FTICR-MS provided diagnostic cross-ring fragments and excellent mass resolution, enabling the localization of sulfate modifications. Finally, an interesting aspect of the TIMS NETD MS/MS coupling concerns selection of the optimal charge state. While NETD works better for highly charged ions in general, isomer separations in TIMS were often more efficient when selecting lower charge states, making the choice of precursor ions all the more important.

**Figure 33:**
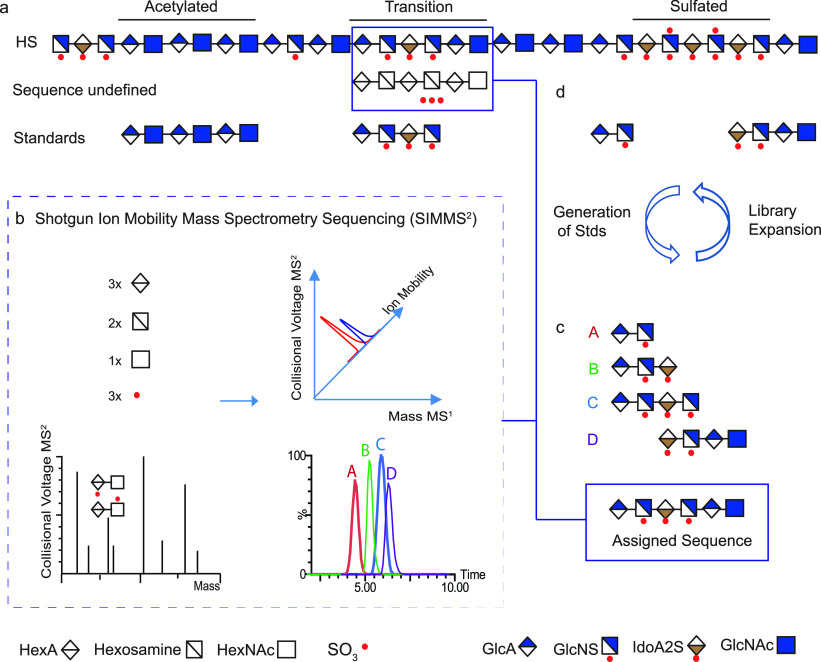
Graphical overview of the SIMMS^2^ strategy for *de novo* glycosaminoglycan sequencing. (a) Characteristic domain structure of heparan sulfate (HS) chains and a matching set of shorter HS oligosaccharide standards. (b) MS and tandem MS provide *m*/*z* values for intact and fragment ions, while ion mobility spectrometry allows for the determination of collision cross sections (CCSs). (c) Through comparison of unknowns with database elements, the library containing *m*/*z* values and CCSs of ions generated from known standards enables the determination of unknown HS sequences. (d) Illustration of the iterative loop process for expanding the CCS data set, enabling continuous development of the library-based SIMMS^2^ strategy. Figure reprinted from ref (527). Copyright 2020 Miller et al. (Creative Commons Attribution 4.0 International License).

Recently, Miller et al. introduced a fragment-based shotgun IM-MS sequencing (SIMMS^2^) approach, utilizing the unique pre-IMS fragmentation capabilities of a Q-DTIMS-ToF platform.^527^ First, a library of ^DT^CCS_He_ values was created using the stepped-field method and 36 HS oligosaccharide standards (dp2–dp10) of well-defined structure, including species carrying 3-*O*-sulfation. CCS values were determined for both intact and CID fragment ions generated from each of the 36 standards, resulting in a library containing sequence–CCS–*m*/*z* trios in numbers far exceeding the number of standard compounds used. This strategy also circumvents certain issues arising from potential reducing-end modification mismatches, by utilizing internal and A-, B-, and C-type fragments. To demonstrate the speed and potential of SIMMS^2^, an unknown hexasaccharide was rapidly sequenced by comparing the CCS values of its fragments to CCSs in the library of known structures. The known sequences, in this case, stemmed from two dp4 standards sharing common structural elements with the unknown hexasaccharide. SIMMS^2^ is currently limited mainly by IMS resolving power, the uncertainty of CCS measurements, and the availability of standards. Improvements are expected in all three aspects in the future, decreasing potential degeneracy in the library (i.e., one CCS value corresponding to multiple structures) and thereby reducing the possibility of structural misassignments. As the method is based on an ever-expanding library of CCS values, it may help circumvent the meticulous analysis of fragment ion spectra that relies heavily on expertise and could represent a step toward high-throughput GAG sequencing. A graphical overview of the SIMMS^2^ workflow is shown in Figure 33.

Because the present review is focused on glycan sequencing and structure elucidation, many excellent studies dealing with GAG–protein interactions were not addressed above.^528−532^ This decision reflects only the scope of the review and not the importance of these interactions or that of the respective studies.

### 6.5 GAG Analysis by Gas-Phase Ion Spectroscopy

As a relatively new field augmenting the GAG sequencing toolbox, GAG analysis by gas-phase ion spectroscopy in the UV and IR range emerged only throughout the past decade.^34,533^ As already mentioned in Section 6.3, the Dugourd group published the first experimental, gas-phase UV spectrum of sulfated disaccharide anions from 220 to 290 nm.^513^ The exact gas-phase molecular structure of the set was further investigated using density functional theory (DFT). The experimental spectra were found to be in reasonable agreement with the calculated spectra and revealed that UV spectroscopy is sensitive to the modes of the sulfate groups present.^213^

The first gas-phase IR spectrum from 3400 to 3700 cm^–1^, using IRMPD spectroscopy in an FT-ICR cell of d-glucuronic and l-iduronic acid monosaccharides, was published by Polfer and co-workers.^534^ They used rubidium to ionize the monosaccharides as positively charged ions and showed that the method is sensitive to the epimerization at C5. Similar results were obtained for the same monosaccharides investigated as anions.^535^

Compagnon and co-workers^188^ used IRMPD spectroscopy from 3200 to 3700 cm^–1^ to record the spectrum of a sulfated monosaccharide, i.e., glucosamine 6-sulfate, as a protonated cation. In their publication, they describe the potential of gas-phase spectroscopy coupled to MS as a tool to differentiate sulfated from phosphated saccharides based on their vibrational modes. Harmonic and anharmonic (VPT2 and finite temperature molecular dynamics) frequency simulations were used to predict the wavenumber of certain OH stretches in the sulfate and phosphate functional groups. Glucosamine 6-sulfate was later used as a model to test the accuracy of a large number of frequency simulation methods.^536^ Methods which are satisfying at higher wavenumbers are shown to fail from 500 and 1700 cm^–1^ as the anharmonic nature of the sulfates challenges the calculations. The best match between experimental and theoretical spectra from 500 to 1700 cm^–1^ was obtained using the computationally most demanding method. DFT simulations were further improved for anionic monosaccharide ions and compared to the experimental spectra.^537^

**Figure 34:**
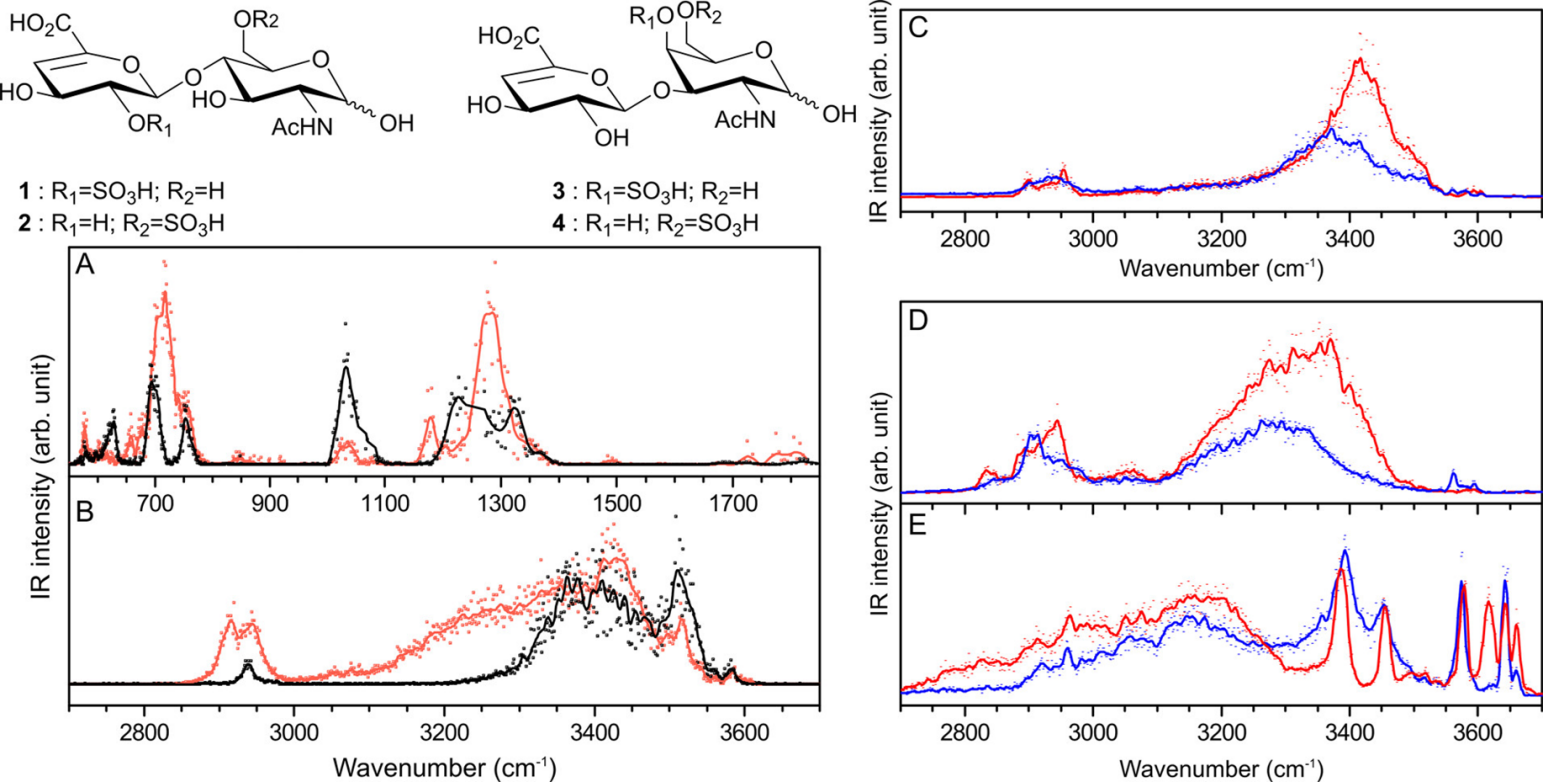
IRMPD spectra of heparan sulfate (HS) and chondroitin sulfate (CS) disaccharides. Scheme: Δ^4,5^ unsaturated disaccharides derived from HS (left) and CS (right). (A, B) IRMPD spectra of deprotonated Hp II-A (red: [Hp II-A – H]^−^) and Hp III-A (black: [Hp III-A – H]^−^) in the 550–1850 and 2700–3700 cm^–1^ spectral ranges. Right panels: IRMPD spectra of CS-A (red) and CS-C (blue) in different charge states. (C) Singly deprotonated [CS-A – H]^−^ and [CS-C – H]^−^. (D) Doubly deprotonated [CS-A – 2H]^2–^ and [CS-C – 2H]^2–^. (E) NH_4_^+^ cationic complexes [CS-A + NH_4_]^+^ and [CS-C – NH_4_]^+^. Figure reproduced with permission from ref (537). Copyright 2017 American Chemical Society.

Disaccharides are the smallest building blocks in GAGs, and consequently, the use of IR spectroscopy advanced most recently to characterize larger fragments. Rizzo and co-workers^538^ published IR spectra from 3200 to 3700 cm^–1^ using messenger-tagging spectroscopy coupled to IM separation of isomeric CS and HS disaccharides as positively charged sodium adducts. At cryogenic temperatures, the resolving power is sufficient to differentiate the five isomers only based on their IR fingerprints. Four isomeric CS and HS disaccharides were investigated from 2700 to 3700 cm^–1^ and two of the set also from 550 to 1850 cm^–1^ using IRMPD spectroscopy (see Figure 34).^537^ The authors explored the use of different charge states, species, and ionization modes on exemplary ions to gain resolving power in IRMPD spectroscopy. In another study, MS^*n*^ in a 3D ion trap analyzer was combined with IRMPD spectroscopy from 2800 to 3700 cm^–1^ for Y- and B-type fragment anions of CS isomers with *O*-sulfation at the C4 and C6 position.^539^ The two isomers cannot be differentiated by MS alone and are challenging in IMS. The resolving power of IRMPD reaches its limitations, yet the isomers can be distinguished by their spectroscopic fingerprints.

**Figure 35:**
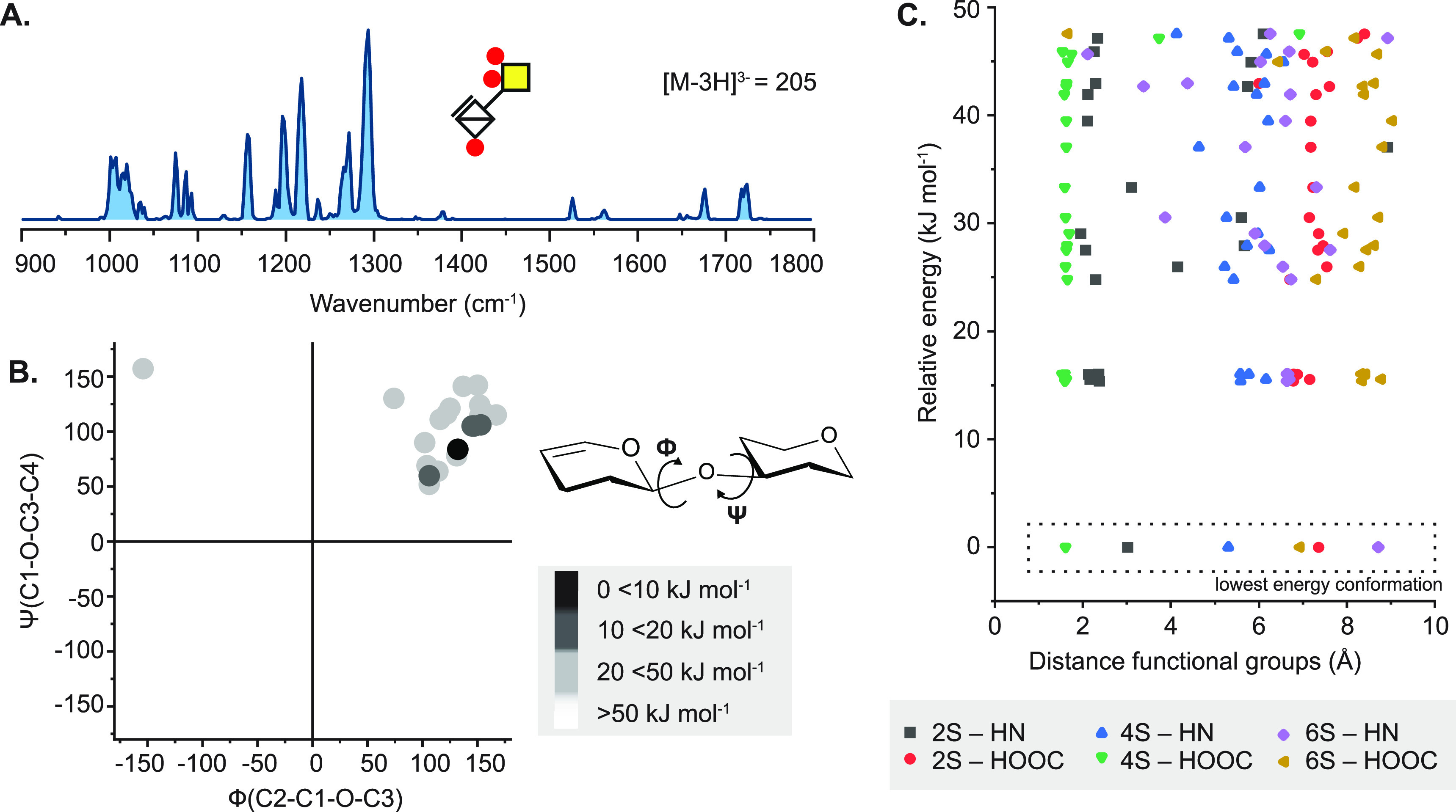
Conformation of chondroitin sulfate disaccharides in the gas phase revealed by IR spectroscopy and quantum chemical calculations. (A) Cryogenic IR spectra of a triply sulfated disaccharide investigated as a [M – 3H]^3–^ anion with *m*/*z* of 205. (B) Calculation of the dihedral angles at the glycosidic bond with respect to the relative energies of the conformers presented in a Ramachandran-type plot for the glycosidic linkage. (C) Calculation of the intramolecular distance of charged sulfates to carboxyl and amide groups in Å in conformers with relative energies Δ*E*_PBE_ < 50 kJ mol^–1^. In the case of the triply sulfated and triply charged disaccharide, the low-energy conformers present in the gas phase are similar to each other. The empty diamond symbol represents a Δ^4,5^-unsaturated hexuronic acid residue. Figure reproduced from ref (540). Copyright 2021 Lettow et al. (Creative Commons Attribution 4.0 International License).

Going larger, IR spectroscopy from 1000 to 1800 cm^–1^ was used to investigate an intact, highly sulfated pentasaccharide, i.e., the synthetic anticoagulant fondaparinux or Arixtra, as a protonated ion and sodium adduct.^204^ The pentasaccharide challenges most MS fragmentation methods due to the fragile nature of the eight sulfate groups. The IR spectra were recorded using both IRMPD spectroscopy and cryogenic IR spectroscopy in helium nanodroplets. The cryogenic temperatures are essential for the efficient IR spectroscopic study of larger GAGs. For a set of synthetic, sulfated HA derivatives, it has been shown that, even for tetrasaccharides, the IR signature of the sulfate groups exhibits a distinct pattern. The charge state, with respect to the number of equivalent, acidic functional groups, plays an important role in IR spectroscopy of anionic GAGs to prevent charge migration and further increase the spectral resolution. Using this high resolving power, the distinct IR signatures of four HS tetrasaccharide anions with varying configurations at C5 revealed a clear spectra–structure relationship, which was further rationalized by quantum chemical calculations.^233^ Structural motifs in the hydrogen bonding network could be deduced for either d-glucuronic or l-iduronic acid. In another study, the IR spectra in combination with quantum chemical calculations of CS/DS disaccharides from bacterial chondroitinase digestion (exclusively as Δ^4,5^ unsaturated uronic acids, SNFG symbol: white diamond) with all known motifs of sulfation revealed that the charge state at the sulfates, which are exclusively deprotonated, defines the size of the gas-phase conformational landscape and therewith the spectral signature (see Figure 35).^540^ In higher sulfated and charged CS/DS disaccharides, as in the triply sulfated disaccharide shown in Figure 35, less low-energy conformers are present than in lower charged CS/DS disaccharides. Quantum chemical calculations aid the understanding of the IR spectra of GAGs to evolve the method and its ability within the analytical toolkit.

## 7 Glycopeptides

The field of glycomics experienced a rapid growth in recent years, and many fundamental insights into structural properties of all glycan classes, including *N*- and *O*-glycans, GAGs, and glycolipids were gained. Especially MS-based *N*- and *O*-glycan analysis found its way as a routine measurement in clinical context due to the fact that alterations in their glycan profile are directly associated with various diseases such as rheumatoid arthritis,^541,542^ diabetes,^1,543^ and cancer.^544,545^ However, most of the information gained on *N*- and *O*-glycans emerged from experiments on the released glycoforms, while very little information is raised from glycoproteins directly. The traditional approach for glycan analysis is mostly separated from peptide/protein analysis to make the process simpler but comes at the cost of losing glycan site information.

This has drastic consequences for our understanding of the biological function of glycans. We lose the ability to study the prevalence for specific glycosylation sites on proteins and the influence of glycan location on biological processes.^546^ The assignment of specific glycan structures in the intact proteome could have a huge impact on our current understanding of structure–function relationships and will likely lead to a boost in finding potential disease biomarkers and therapeutic targets.^547^ Therefore, the field of glycoproteomics aims to merge glycomics and proteomics to obtain a comprehensive picture of both glycan and peptide identity, for any given glycoprotein in a cell or tissue. MS-based analytical techniques play an essential role in this process as they provide the necessary resolution to enable global glycoprotein analysis. MS often provides more detailed information compared to solution-based approaches, e.g., immunological assays, because it is possible to characterize and identify species on a molecular level.

### 7.1 Top-Down Analysis of Glycoproteins

The MS methods used in glycoproteomics are very similar to classical “bottom-up” and “top-down” proteomic approaches. While top-down glycoproteomics focuses on the direct analysis of intact glycoproteins via LC-MS/MS, bottom-up glycoproteomics first utilizes proteolytic enzymes to digest the glycoproteins and subsequently characterizes the glycopeptides by LC-MS/MS.

The advantage of top-down glycoproteomics is the information gained on the complete amino acid sequence, which includes all post-translational modifications. The charge state and the isotopic distribution of each precursor embody a very detailed but extremely complex set of information on the glycoprotein structure with minimal sample preparation. This information richness also has its downsides: low charge states (which often represent the native conformation of the analyte) result in very high glycoprotein precursor *m*/*z* and require suitable high-mass instruments with extraordinary resolution and/or complex detection systems^548^ to unravel the data.

On the other hand, high charge states (which are often the result of denaturing, non-native conditions) lead to low precursor *m*/*z* with multiple charge states that are readily detectable by most mass spectrometers. The wide distribution of charge states, however, can significantly dilute signal sensitivity and leads to overlapping of different glycoprotein precursors, resulting in problems with identification. This situation is aggravated due to the macro- and microheterogeneity of glycosylation sites. Their inherent structural diversity leads to a diverse mixture of differently glycosylated proteins, which represents a major challenge for the comprehensive analysis of glycoproteins via intact glycoproteomics.^549^ Therefore, the complex application of top-down analysis still hampers its routine use in therapeutic and diagnostic approaches despite the potential advantages in gained information.

### 7.2 Bottom-Up Analysis of Glycoproteins

#### 7.2.1 Sample Preparation and Condensed-Phase Separations

**Figure 36:**
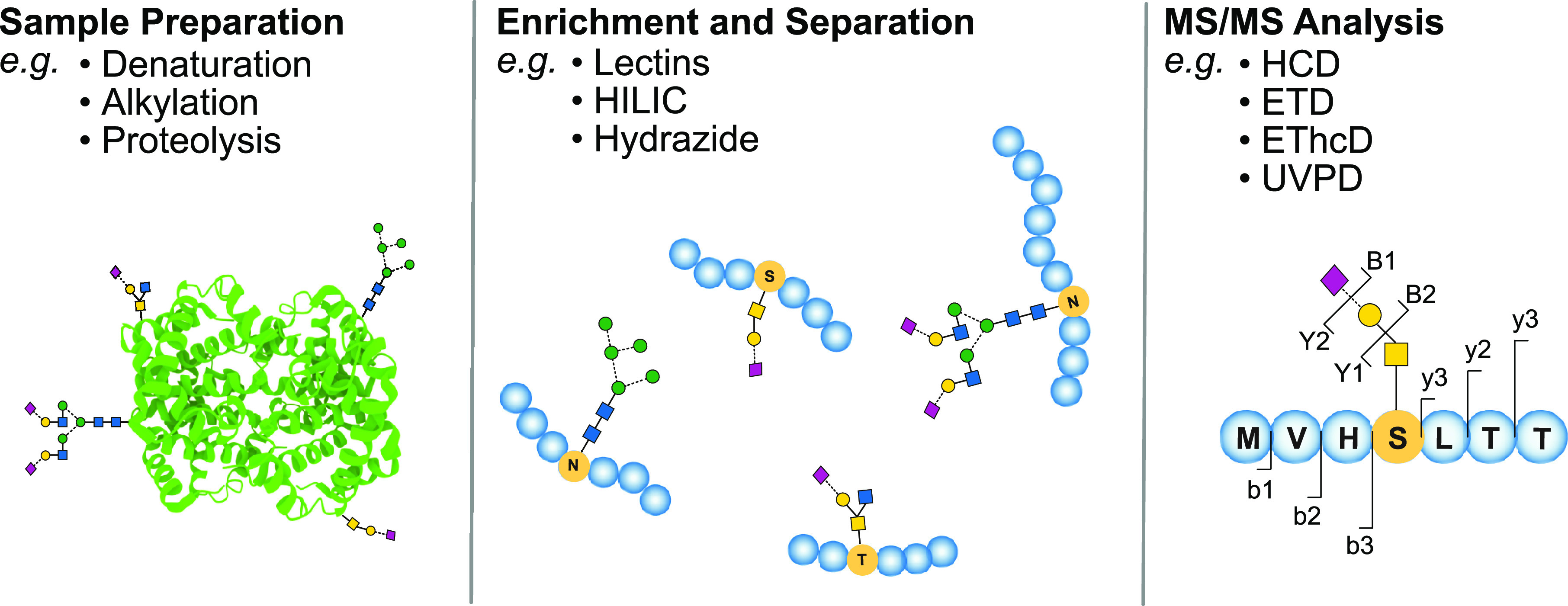
General scheme of bottom-up glycoproteomics workflows. Glycoproteins are extracted and enriched from biological samples. The glycoproteins are digested by proteolytic enzymes, and the resulting glycopeptides are enriched from the digestion mixture. The concentrated glycopeptides are separated via HPLC or CE to facilitate isomeric identification and subsequently analyzed via MS/MS. Complete glycopeptide characterization requires three elements: the sequencing of the peptide backbone, the sequencing of the glycan moiety, and the localization of the glycosylation site within the amino acid sequence.

Currently, it is more straightforward to digest glycoproteins with proteolytic enzymes into smaller glycopeptides to reduce the complexity and simplify the analysis by MS. Although the data set for glycopeptides needs to be combined and backtracked to obtain information on the intact glycoprotein, modern strategies facilitate this process and allow a high coverage of both a glycan and peptide moiety. This led to the rise of bottom-up approaches as a prominent technique for the analysis of glycosylated proteins already today. Bottom-up experiments follow a typical workflow that can be divided into sample preparation, enrichment/separation strategies, and MS analysis (Figure 36).

Sample preparation generally includes an optional enrichment of the glycoprotein from its complex matrix and the subsequent proteolytic digestion with a suitable enzyme. The proteolytic digestion mixture can be directly analyzed by MS, but the great variety of glycans attached to each glycan site (microheterogeneity) and their heterogeneous site occupancy (macroheterogeneity) results in low abundancies for each glycopeptide. Furthermore, glycopeptides generally show lower ionization efficiencies compared to nonglycosylated species; therefore, it can be necessary to separate the glycopeptides from their complex matrix before MS analysis. There are several enrichment strategies available. The most prominent strategies include for example lectin-based approaches, HILIC SPE workflows, metabolic labeling, or covalent conjugation to hydrazide resins.^4^

Although enrichment strategies can concentrate the glycopeptides from the digestion mixture, they cannot completely compensate for the low abundance of glycosylated species. Commonly used data-dependent acquisition (DDA) approaches for MS analysis are often struggling with these low abundancies as MS/MS acquisition is generally triggered by intensity thresholds. Targeted and semitargeted approaches are alternative approaches to facilitate data acquisition, but including all possible glycan structures in inclusion lists is a great challenge due to their large heterogeneity.^550^ A promising approach to overcome this problem was developed by Woo et al. in 2015, which combines metabolic labeling with an analysis of the isotope pattern. By introducing a bromide-containing azide or alkyne function into the glycan residue via metabolic labeling, the enrichment and proteolytic digestion could be combined into one coherent workflow called IsoTag.^551^ Furthermore, the subsequent analysis of the characteristic bromide isotopic pattern via MS enables a pattern-searching algorithm for both detection and validation of isotopically encoded species. This approach gained increasing attention as it enables us to identify glycopeptides in complex mixtures in MS experiments even at low concentrations.^547,552^

Due to the large variety of glycan structures and the possibility of multiple glycosylation sites on the glycopeptides, enrichment of the glycopeptides is often accompanied by an additional separation step before MS characterization. This allows the separation of isomeric structures and simplifies the identification of glycopeptides by MS. Traditionally, reversed-phase methods with C8 and C18 stationary phases are applied to peptides due to the hydrophobic character of the peptide backbone; however, the hydrophilic glycan moiety is not well retained, and therefore only small or no isomeric resolution of glycopeptides is achieved. This led to the application of more specific chromatographic methods such as PGC^553^ and HILIC,^554^ which have unique properties to resolve both peptides and glycopeptides, on an isomeric level. Furthermore, capillary electrophoresis showed its potential as a promising separation technique as it allows the separation of isomeric glycopeptides in very short time frames.^555,556^ Further details on sample preparation and separation strategies are summarized in recent review articles.^4,557,558^

#### 7.2.2 Tandem Mass Spectrometry for Glycopeptide Analysis

Complete characterization of glycoproteins requires not only sequencing of the protein backbone and glycan chains but also the localization of glycosylation sites within the amino acid sequence. This is especially difficult for *O*-glycans because, in contrast to *N*-glycosylation, there is no defined consensus peptide sequence for the various types of *O*-glycosylation. Furthermore, as the intact glycopeptide mass is generally not sufficient to characterize the components,^559^ it is necessary to fragment it via MS/MS. One of the major analytical challenges in the analysis of glycopeptides is the generation of informative fragments to fully identify a peptide and glycan moiety at the same time. In this regard, it is important to mention that peptide^560^ and glycan fragments^54^ are denoted using very similar nomenclatures. In order to avoid confusion, it is therefore common in glycoproteomics to denote peptide fragments using small letters (e.g., b/y) and glycan fragments using capital letters (e.g., B/Y).

Traditional slow-heating techniques such as CID are commonly used ion dissociation methods in glycoproteomics as they are built in many mass spectrometers due to their simple implementation. Low-energy CID predominantly leads to the dissociation of the weakest bonds, resulting mainly in glycosidic bond cleavages (B- and Y-fragments), and the loss of the labile sugar moieties from the peptide backbone. As such, it provides little information on the primary structure of peptide constituents, and the information regarding the sites of glycosylation is lost. High-energy CID further generates some peptide backbone fragments (b/y). The combination of low- and high-energy CID is mostly applied for sequencing the glycan and peptide moieties separately^561^ and represents a robust sequencing method that is well suited for a broad range of *N*- and *O*-glycosylated peptides.^562^ However, as it hardly can generate Y_1_ fragments, it is more commonly used for determining *N*-glycosylation, as the prediction of *N*-glycosylation sites is more straightforward due to the defined attachment points.

For a more detailed analysis of glycan attachment sites, higher-energy CID (HCD), which is implemented into modern Orbitrap instruments, is a promising alternative. In contrast to standard CID, typical HCD ion activation mainly results in peptide backbone fragmentation as well as fewer oxonium ions resulting from glycan fragmentation. Furthermore, b/y type fragments of the peptide backbone that still contain an *N*-acetylglucosamine (GlcNAc) moiety (Y_1_) are often generated, which provide clues to the location of glycosylation.^563^ Recent studies showed that, similar to standard CID, higher HCD collision energies predominantly generate b/y-fragments, while lower collision energies yield B/Y-fragments which are preferable for glycan characterization. The combination of multiple collision energies in one MS/MS run, termed stepped-energy HCD, is used for high-throughput application and is very popular in the studies of *N*-glycopeptides.^73,564^ In contrast to the defined amino acid sequence for *N*-glycosylation, *O*-glycosides generally have multiple serine or threonine residues that can serve as potential attachment points. The exact determination of *O*-glycosylation sites therefore often requires orthogonal approaches in addition to the popular HCD methods. The *O*-glycosidic bond connecting the peptide to the glycan is more labile than the amide bond of *N*-glycans and readily dissociates during vibrational activation in the mass spectrometer.

**Figure 37:**
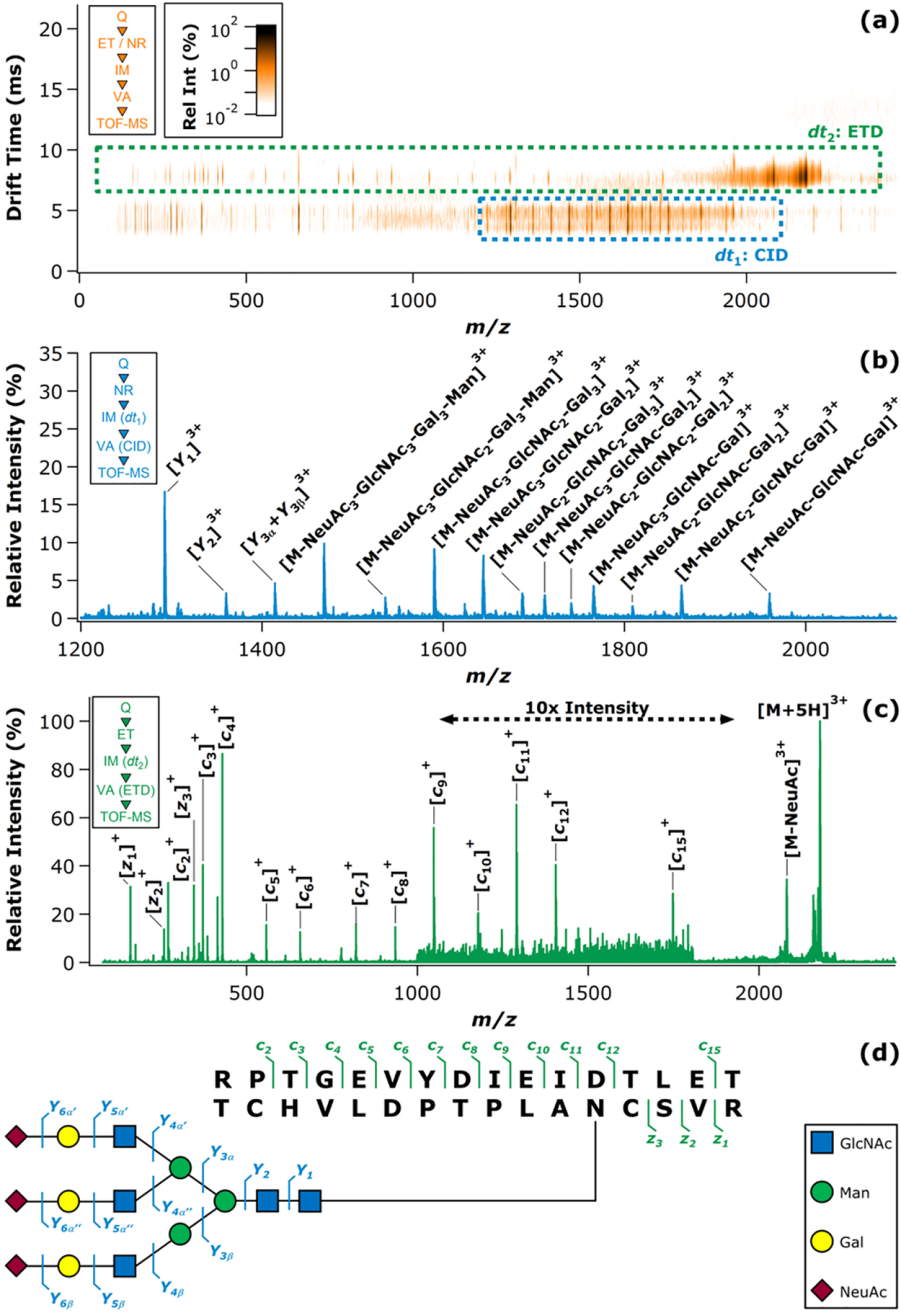
Ion-mobility-resolved parallel fragmentation of a triantennary, fully sialylated complex *N*-glycopeptide structure via collision-induced dissociation (CID) and electron transfer dissociation (ETD) in positive ion mode. (a) 2D plot of glycopeptide precursor intensity against *m*/*z* and drift time. Precursor activation via ETD results in charge reduction which allows separation of activated (dt2, green) and nonactivated precursor (dt1, blue) on the IMS level. (b) Tandem mass spectrum of the glycosylated species at dt1 after CID activation. (c) Tandem mass spectrum of the glycosylated species at dt2 after ETD activation. (d) Visual representation of the glycopeptide precursor and the resulting fragmentation pattern after CID (blue) and ETD (green) fragmentation. Figure reprinted with permission from ref (569). Copyright 2017 The Royal Society of Chemistry.

ExD methods such as ETD and ECD are more likely to generate the preferential dissociation of the peptide backbone before glycosidic cleavage.^565^ They produce predominantly c/z-fragments, whereas the glycosylation site remains intact. This allows for the identification of the peptide sequence and the localization of the modification site, which is especially helpful in O*-*glycoproteomics.^566,567^ Modern MS instruments can combine electron-based and vibrational activation (EThcD,^568^ ETD/CID^569^) into a hybrid approach that allows efficient glycopeptide characterization within a single MS/MS experiment. In principle, the hybrid fragmentation can be applied in a sequential or combinatorial manner. Typical combinatorial approaches perform ETD and apply CID on the undissociated charge-reduced species. Low-energy CID results in glycan fragmentation, while ETD allows cleavage of the peptide moiety. The information on both can be combined into a comprehensive data set.^570^ This method is relatively straightforward to apply and produces orthogonal tandem mass spectra to fully identify the glycopeptide (Figure 37). A relatively new method performs ETD and applies HCD on all ions, resulting from this process in a sequential way (EthcD). The hybrid fragmentation method generates considerably more fragment signals, which can be used for glycopeptide analysis and database matching. The requirements for a comprehensive and rapid characterization significantly differ for *N*- and *O*-glycopeptides and were recently summarized.^571^ Stepped-energy HCD and EthcD replaced CID in terms of popularity and are widely used for glycopeptide characterization. However, a general drawback of all ETD hybrid methods is the relatively low signal sensitivity for larger glycopeptides because they are commonly observed as lowly charged ions with high *m*/*z* values which typically produce less fragment ions with ETD.

Complementary alternatives to collisional and electron-based techniques are photofragmentation approaches by IRMPD or UVPD. IRMPD is not very common in glycoproteomics as it is technically demanding and typically only used in ion trap instruments, for example, as a fragmentation method in FTICR. Although IRMPD yields very similar fragmentation spectra to traditional CID, it has several advantages. One major advantage of IRMPD over traditional CID is that no collision gas is required for ion activation. Therefore, there is no degradation of the vacuum inside the instrument which speeds up the analysis and minimizes ion losses during measurement.^572^ Furthermore, the efficiency of CID fragmentation can be highly dependent on the precursor mass, while IRMPD can be easily applied for larger precursor ions and even intact proteins.^573^ IRMPD was successfully applied for the analysis of *N-* and *O*-glycopeptides^574,575^ but is currently limited by the complex instrumental demands.

Another dissociation method employing ion–photon interaction, UVPD, recently gained attention for the analysis of glycans and glycoconjugates.^32^ Here, the rapid absorption of energetic photons by molecular ions leads to the population of excited electronic states, opening up new dissociation pathways that lead to highly informative fragmentation patterns. Many of the fragment ions formed are usually not accessible by conventional ion activation methods, which enables a much more detailed structural assignment. UVPD efficiently generates fragment ions from both the peptide and glycan components, enabling the simultaneous analysis of the two constituents by tandem MS. Photofragments resulting solely from peptide bond cleavages (a/x-fragments) that still carry the sugar moieties also allow for the localization of the glycosylated sites. In addition, less abundant B-, Y-, C-, and Z-ions and cross-ring cleavages provided information on the connectivity of sugar moieties.^576^ UVPD was successfully applied on *N*-glycopeptides,^577^ but it could be even more helpful for the characterization of *O*-glycosylation. *O*-Glycopeptides are often very acidic and generally hard to ionize in positive ionization mode, which is traditionally used for glycopeptide analysis. UVPD generates extensive fragmentation for deprotonated species and showed very good sequence coverage for both the glycan and peptide moiety, in recent studies.^578^ Although photodissociation via UVPD was introduced more than 30 years ago, its application was limited by the low signal-to-noise ratio of mass spectra and difficult light sources.^32^ However, recent technical advances will facilitate the analysis of biomolecules via UVPD, and it is expected to take an important role in the future glycoproteomics field.

#### 7.2.3 Ion Mobility–Mass Spectrometry of Glycopeptides

In many cases glycans and glycopeptides have multiple coexisting isomers that exhibit an identical atomic composition and, as a result, an identical mass. Even with sophisticated MS/MS- and software-based approaches, it is often simply not possible to clearly assign a particular molecular structure by MS alone. A powerful technique that has the ability to compensate this shortcoming is IMS. The combination of IM-MS is a promising orthogonal tool for the analysis of biomolecules and glycoconjugates in particular.^579−581^ It showed great potential as a prefilter before MS detection and allows us to separate glycopeptides from nonglycosylated peptides in the gas phase. In this function it complements the enrichment/separation step to prevent overlapping of precursors and to increase identification confidence. This is especially important for *O*-glycosylated species as they are traditionally harder to enrich via conventional methods.^582^

Furthermore, IMS can be used to distinguish isomeric glycopeptides with identical peptide sequence but different glycosylation sites^583^ or different glycoforms^584^ before ion activation with ETD or CID.^585,586^ This combination can increase peptide sequencing coverage^587^ and glycan composition information.^582^ In addition to the orthogonal separation dimension produced by the combination of IMS and MS, the resulting drift times can be used to obtain CCS values, which can provide structural information on the glycopeptides as they represent the rotationally averaged surface area of the ions. CCS values can be used as additional identification parameters to complement retention time and mass information to increase identification confidence. Various databases for CCSs of glycopeptides have been created,^414,588^ and it could be feasible to predict the glycopeptide identity solely based on their CCSs in the near future, comparable with an approach for nonglycosylated peptides.^589^

For very similar isomers, the differences in CCS decrease with increasing glycopeptide size, and therefore IM separation becomes less efficient for larger glycoconjugates. This often complicates the differentiation of intact ions. The fragmentation of intact precursors, however, can lead to diagnostic fragments with distinct CCSs, from which information on the structure of the precursor can be deduced. Based on mono- and disaccharide fragments cleaved from glycopeptides, it is possible to identify glycan motifs independent of the peptide sequence.^353^ Larger fragments can also be used to identify the branching from complex *N*-glycopeptides which, as a result, can be used to deduce larger oligosaccharide structures.^379^

**Figure 38:**
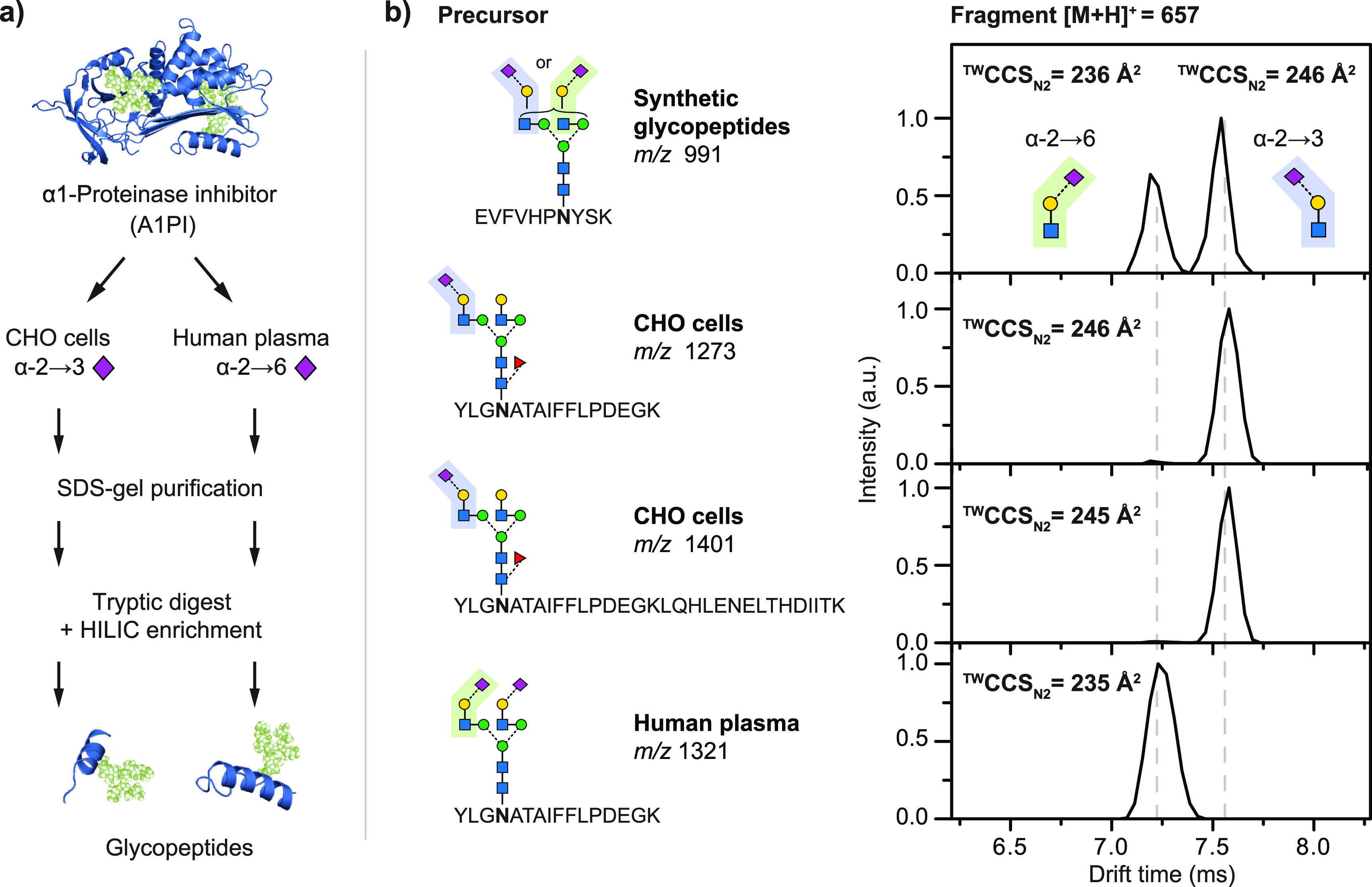
Ion mobility–mass spectrometry (IM-MS) workflow for regiochemistry analysis of *N*-acetylneuraminic acid (Neu5Ac) linkages in a1-proteinase inhibitor (A1PI). (A) A1PI isolated from human plasma and recombinantly expressed in Chinese hamster ovary (CHO) cells was purified and digested with trypsin, and the glycopeptides were HILIC-enriched. (B) Fragmentation of the obtained glycopeptides and subsequent IM-MS analysis of the characteristic B_3_-trisaccharide fragments (*m*/*z* 657) enabled the differentiation of α2,3- from α2,6-linked Neu5Ac. The observed fragment drift times and ^TW^CCS_N2_ are independent of the underlying precursor sequence. Reprinted from ref (590). Published by The Royal Society of Chemistry. Copyright 2016 Hinneburg et al. (Creative Commons Attribution 3.0 Unported License).

However, fragment CCS values not only are useful to reconstruct the structure of larger oligosaccharides but also have a great potential to be used for the rapid screening of common structural features. In many diagnostic applications, a full structural elucidation of the glycans and glycopeptides is not necessary, and it is often sufficient to identify specific glycan epitopes. Such reoccurring patterns in *N*- and *O*-glycosylation are often found in fucosylation and sialylation. Under low-energy CID conditions, glycan-only fragments are usually released from intact, cationized glycopeptides. This aspect is usually regarded as a major drawback of CID in glycoproteomics, as it precludes glycosylation site analysis; however, it turned out to be exceptionally useful for the IM-MS analysis of sialylation patterns (Figure 38). Upon CID, diagnostic antennary trisaccharide fragments, which showed characteristic drift times depending on the regiochemistry of the sialic acid linkage, were obtained. This enabled the rapid and reliable identification of the sialic acid connectivity based on fragment CCSs.^590,591^ A similar fragment-based approach was used for the analysis of fucosylated glycans via IM-MS; however, until now it was only applied for released glycans, and it still needs to be verified for fucosylated glycopeptide structures.^374^

#### 7.2.4 Spectroscopic Approaches for Glycopeptides

In recent years, IR-based spectroscopic approaches have been introduced for the analysis for many glycan classes. In contrast to MS and IMS, which produce mass- and surface-related information on a given species, IR ion spectroscopy is able to generate a picture of internal interactions and molecular conformations. While IRMPD is regularly used as slow-heating ion activation in various tandem MS approaches for glycopeptides, IR spectroscopy of glycopeptides is often hindered by the intense signals generated by the peptide backbone. IR spectra of peptide-related species are generally dominated by the amide I, II, and III bands. The shape and intensity of the amide I/II bands are characteristic for the globular folding of peptides/proteins and can be used to deduce information on their secondary structure.^592,593^ The intensity of all three absorption bands is usually very high due to the large number of aligned amide oscillators inside the peptide backbone. *N*-Acetylated glycans, e.g., GlcNAc and GalNAc structures, are overshadowed in these regions.^574^ Furthermore, also other distinct differences in the vibrational signature of glycans such as connectivity and configurational isomers usually appear above 1300 cm^–1^.^35^ The most pronounced spectral difference of oligosaccharides and peptides is found in the IR fingerprint region around 1100 cm^–1^^574^ and the hydrogen stretching region (3400–3750 cm^–1^). The comprehensive characterization of a glycan moiety on an intact glycopeptide is highly impeded as most of the informative frequency range is overshadowed by the peptide backbone. At that moment, the only feasible approach is the chemical/enzymatic release of the glycans and subsequent IR spectroscopy.^202^ Similar to IMS workflows, however, a potential solution to this problem could lie in fragmentation-based approaches. Prior fragmentation via slow-heating ion activation methods such as CID could cleave the glycan residue of the peptide backbone in the gas phase. IR ion spectroscopy could then be applied on the glycan component to deduce structural and conformational information.^35^

Another promising alternative could be ion spectroscopy in the UV range. Peptides and proteins can be readily excited due to the presence of aromatic side chains like phenylalanine, tryptophan, or tyrosine and can be used to generate fingerprints of the electronic structures of these chromophores. Neutral, deprotonated, and radical aromatic amino acids display strong bathochromic shifts in the UV spectra which can be associated with the reactivity and biological function of the respective peptide.^594,595^ The application on the structural characterization of glycopeptides, however, has yet to be implemented.

## 8 Glycolipids

### 8.1 Structure and Analytical Challenges

Glycolipids are important glycoconjugates and essential components of biological membranes, originating from the glycosylation of lipids.^596^ They occur in living organisms ranging from bacteria to humans and fulfill numerous vital functions, which make glycolipids indispensable for the development and differentiation of multicellular organisms.^597−599^ The amphiphilic molecules can easily insert their hydrophobic lipid tail into cell membranes, while the glycan headgroup points outward into the extracellular medium. This configuration enables glycolipids to mediate cellular interaction and signaling processes^600,601^ and to modulate the function of membrane proteins.^602^ Furthermore, glycolipids are required to ensure the permeability barrier function of skin.^603^ The impairment of β-Glc ceramide catabolism in Gaucher disease results in an increased skin permeability and a concomitant risk of dehydration. Gaucher disease^604^ and other lysosomal storage disorders^605,606^ also frequently affect neural functions. This finding illustrates the importance of glycolipids for the functioning of the nerve system, e.g., for the myelination of axons.^607^ Furthermore, several glycolipids bearing α-glycosidic bonds were found to play an important role in innate immunity.^608,609^ In this context of antibody–antigen interactions, seemingly small structural details, such as the stereochemistry of the glycosidic bond, can affect the antigenic activity of glycolipids tremendously.

**Figure 39:**
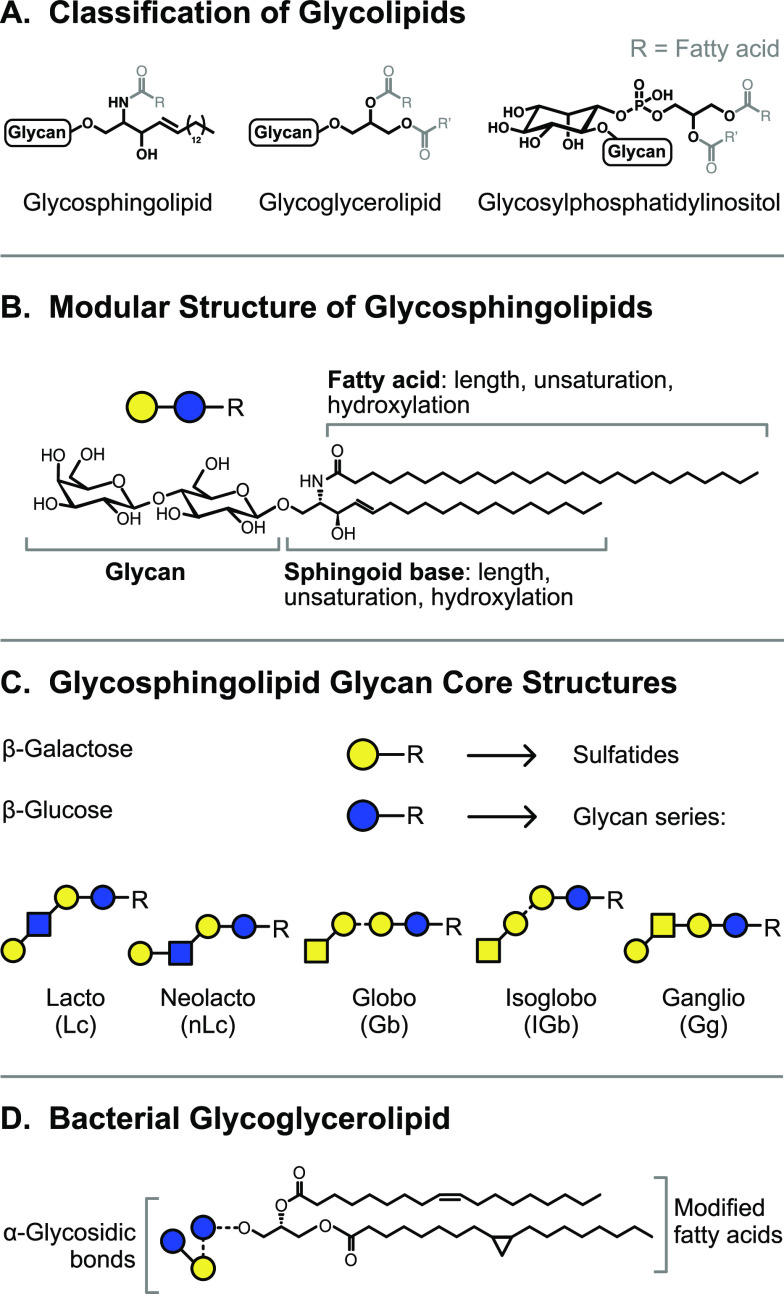
Structural diversity of glycolipids. (A) Glycolipids are classified according to the lipid core structure into three categories. (B) The most common glycolipids in mammals are glycosphingolipids, which are assembled in a modular fashion from a sphingoid base, a fatty acid, and a glycan headgroup. (C) The first monosaccharide attached to ceramides in mammals is either β-Gal or β-Glc. The latter can be elongated to yield common glycan core structures. (D) Bacterial glycoglycerolipids often contain building blocks that are uncommon for mammalian glycolipids, including α-linked monosaccharides and lipid chain branching or cyclopropane rings. The glycoglycerolipid shown here was isolated from *Lactobacillus plantarum*.

Glycolipids are divided into three categories according to the lipid class (Figure 39A): glycosphingolipids (GSLs), glycoglycerolipids, and glycosylphosphatidylinositols. However, the overwhelming majority of glycolipids in mammalian cells is constituted by GSLs.^596^ In GSLs, the glycan headgroup is attached to an amino alcohol, a so-called sphingoid base, which is mainly sphingosine in mammalian GSLs.^610^ In shorthand nomenclature, sphingosine is denoted as d18:1, indicating the number of hydroxyl groups (m = mono, d = di, t = tri), the total length of the hydrocarbon chain (18), and the number of C=C double bonds (1), separated by a colon. Other common sphingoid bases are phytosphingosine (t18:0) and sphinganine (d18:0).^611,612^ Before the attachment of a glycan to the sphingoid base, the latter is *N*-acetylated by a fatty acid to form a ceramide (Cer).^613^ Both lipid chains of the ceramide can vary in length, degree, and position of unsaturation and hydroxylation.^611^ Beyond the structural diversity of the individual building blocks, the structural richness of GSLs also results from the combinatorial, modular assembly of the two lipid chains and the glycan headgroup (Figure 39B).

Even though the number of imaginable glycan structures is countless, only few glycan core structures were identified in mammalian GSLs. Typically, either β-Glc or -Gal is attached as the first monosaccharide to ceramide in mammals. Gal can be sulfated to yield so-called sulfatides but is usually not extended by further monosaccharides.^596^ Glc, on the other hand, is often extended to yield typical glycan structures classified into the lacto, neolacto, globo, isoglobo, and ganglio series (Figure 39C).^610^ Based on their acidity, GSLs are further divided into neutral and acidic GSLs. The latter are either sulfatides or bear at least one sialic acid residue. Acidic glycolipids with sialic acids are historically designated as gangliosides and are mostly expressed in the central nerve system and, in particular, the brain.^614^ Gangliosides are labeled according to the historical nomenclature of Svennerholm,^615^ which is still employed today and therefore explained in the following. Notations such as GM3 or GD1 start by the letter G (=ganglioside), followed by M, D, or T (=mono, di, or tri), designating the number of sialic acid residues, and a number referring to the relative position of the ganglioside after migration on a TLC plate.

GSLs also occur in plants, fungi, and bacteria, which often exhibit uncommon sphingolipids with chain branching, hydroxylation, and cyclopropane rings.^612^ Glycoglycerolipids are also much more common in plants and microbes.^596^ In addition to lipid modifications, bacterial glycolipids can exhibit abundant α-linked glycans, as shown for a glycoglycerolipid extracted from a *Lactobacillus* species (Figure 39D).^616^ Bacteria also present another class of macromolecules composed of a glycan and lipid part, the so-called lipopolysaccharides. However, those are not classified as glycolipids but saccharolipids^617^ and will not be discussed herein. Glycosylphosphatidylinositols that serve as anchors for membrane proteins and are structurally very different from GSLs and glycoglycerolipids will not be addressed either. In view of the huge diversity of glycolipids found in flora and fauna, the following sections will be restricted to the analysis of mammalian GSLs.

The structural analysis of glycolipids poses several challenges arising from (1) the amphiphilicity, i.e., opposed properties of the glycan and lipid part, (2) isomerism in the glycan headgroup, further complicated by modifications such as sulfation, and (3) heterogeneity in the lipid chains, induced by varying chain lengths, unsaturation, and degree of hydroxylation. Recent progress in glycolipid analysis can be ascribed mainly to improvements in separation, innovation of ion activation methods, chemical derivatization strategies, and novel spectroscopic techniques.

Accurate profiling of complex GSLs usually requires the application of separation techniques prior to MS analysis.^618^ The amphiphilic character of GSLs challenges the choice of ideal conditions for chromatographic separation and has resulted in two different modes of operation in liquid chromatography.^619^ Separation on polar stationary phases resolves glycan headgroups, while different ceramide moieties coelute. Reversely, ceramide moieties can be separated on reversed-phase HPLC columns to the detriment of glycan headgroup separation. Recently, an LC-MS/MS method employing a chiral LC column and multichannel–multireaction monitoring was introduced to analyze both the glycan and ceramide.^620^ This experimental setup allows for the simultaneous analysis of neutral GSLs, gangliosides, and sulfatides with different ceramide moieties. The chiral column is also adapted to separate diastereoisomers such as Glc- and GalCer (d18:1/18:0), which were separated by more than half a minute. Chip-based chromatographic methods also hold much potential for glycolipid profiling. Gangliosides and sulfatides were identified and quantified using MS combined with a nano-HPLC chip, which provided reproducible retention times and allowed for efficient separation of isomeric glycolipids.^621^ Another approach for separate glycan profiling consists of enzymatic release of glycans from glycolipids and chromatographic analysis of released glycans, e.g., by porous graphitized carbon (PGC) chromatography.^622,623^

### 8.2 Ion Activation Methods for Glycolipids

Historically, glycolipids have been analyzed by CID MS/MS with or without prior chromatographic separation.^624^ CID of even-electron glycolipid ions does, however, not result in diagnostic fragments that allow for the identification of structural details. Typical fragments of neutral GSLs in positive ion mode result from the loss of water, neutral loss of the fatty acid, and neutral loss of the glycan headgroup due to cleavage of the relatively weak glycosidic bond.^625^ A universal fragment of sphingosine-containing GSLs searched for in precursor ion scans is doubly dehydrated sphingosine at *m*/*z* 264.^609^ CID does not induce intrachain fragmentation and therefore yields information neither on C=C bond positions in the lipid chain nor on the stereochemistry in the glycan headgroup (Figure 41A). However, relative intensities of fragments at different collision energies can differ between isomers, even though the fragments are not unique. Such an approach thus requires calibration with standards, as was shown for α- and β-GlcCer.^626^ Multistage MS can reveal further information that is not available by single CID MS/MS. MS^*n*^ of deprotonated ceramides in a linear ion trap allowed identification of different sphingoid bases, hydroxylation, and different isomeric structures.^91^

**Figure 40:**
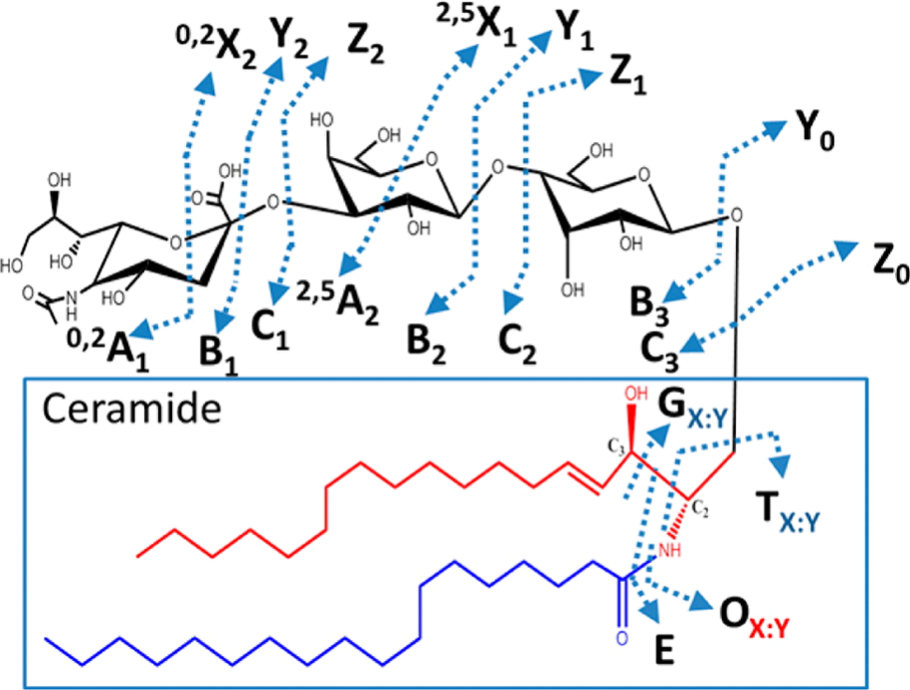
Illustration of fragment ions produced by ultraviolet photodissociation (UVPD) of GSLs. Contrary to collision-induced dissociation, UVPD induces cross-ring cleavage (A/X-ions) in addition to glycosidic bond cleavage. Furthermore, irradiation with 193 nm UV light causes several unique cleavages within the sphingoid base and the fatty acid of the ceramide tail. Figure reproduced with permission from ref (627). Copyright 2013 American Chemical Society.

Alternative activation methods besides CID have been applied to glycolipids even though their usage has remained restricted. For example, UVPD of glycolipids was shown to yield more diagnostic fragments than CID and HCD by inducing cross-ring cleavages in the glycan headgroup in addition to several C–N and C–C cleavages in the sphingoid base and fatty acid (Figure 40).^627^ Isobaric gangliosides were thus differentiated in an Orbitrap mass spectrometer by irradiation with 193 nm UV light. ExD methods including ECD, EDD, and EID were also tested for glycolipid analysis, and ECD was found to induce extensive fragmentation of the GM1 ganglioside, which enabled characterization of the glycan sequence and both lipid chains of the ceramide.^628^ In this respect, ECD largely outperformed other dissociation techniques typically applied to FTICR-MS, including IRMPD and EDD. EID was established as a useful technique for the analysis of small glycoconjugates including glycolipids and yielded informative fragments of the gangliosides GD1a and GD1b, contrary to CID.^87^ EID alone revealed only little information about the lipid tails in the ceramide but is to date the only tandem MS method allowing for the distinction of *cis*/*trans* double bond isomers in lipid chains.^629^

### 8.3 Derivatization Strategies

**Figure 41:**
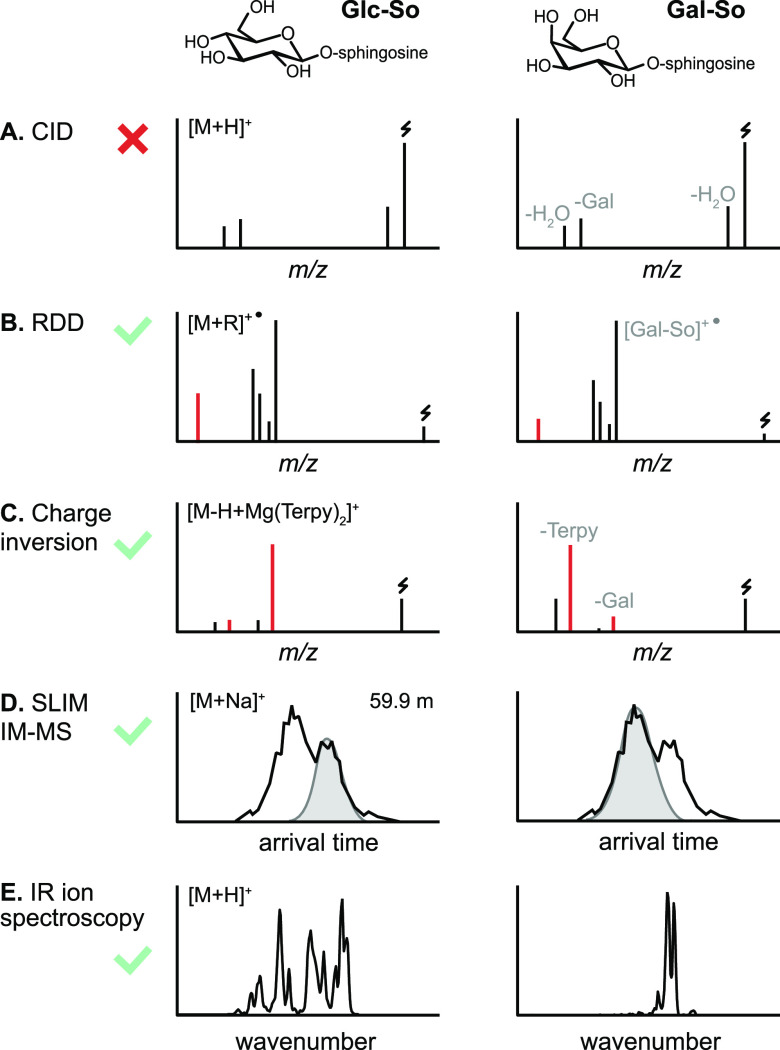
Comparison of the performance of several MS-based techniques in distinguishing between the diastereoisomeric glycolipids Glc- and Gal-sphingosine (So). (A) Classical CID induces cleavage of the glycosidic bond and concomitant loss of the stereochemical information. (B/C) RDD and charge inversion induce alternative fragmentation mechanisms and yield different relative intensities of fragments. (D) SLIM IM-MS can partially separate sodiated Glc and Gal sphingosine after four passes (ca. 60 m). (E) Gas-phase IR spectroscopy is sensitive toward stereochemical changes and yields diagnostic spectroscopic fingerprints for Glc and Gal epimers.

Besides different ion activation methods, chemical derivatization strategies can help to characterize glycolipid structures in combination with classical CID fragmentation. Glycan sequences have been investigated by permethylation since the implementation of the derivatization strategy 50 years ago.^630^ Permethylation was recently adapted to achieve relative quantification of glycolipids by differential isotope labeling and RPLC-MS/MS. The protocol is based on a comparison of ^12^C-permethylated samples with ^13^C-permethylated internal standards.^631^ The need for chromatographic separation to resolve glycolipid isomers can be circumvented by other, more specialized derivatization strategies. For example, lipid and glycolipid isomers form informative fragments upon radical-directed dissociation (RDD).^632−634^ RDD is based on covalent or noncovalent modification of the analyte by bifunctional molecules that contain both a radical initiator and a functional group that can be attached to the analyte. Upon irradiation of the lipid ion–molecule complex, a radical is formed, which is subsequently activated by CID to induce fragmentation within the lipid moiety. The fragmentation pattern reveals the position of C=C bonds, branching, and hydroxylation in the hydrocarbon chain.^632,634^ RDD was furthermore employed to quantitatively distinguish between Glc and Gal headgroups by noncovalent attachment of a modified crown ether bearing a photocleavable C–I bond (Figure 41B).^633^ The approach is only applicable to lyso-GSLs, which lack the fatty acid and therefore exhibit a primary amine. Glc and Gal ceramides do not interact with the crown ether but can alternatively be distinguished by their reactivity toward boronic acid.^633^ A different approach used to distinguish between Glc and Gal ceramides is based on gas-phase ion chemistry and MS/MS.^635^ Deprotonated GSLs form charge-inverted complexes with terpyridine–magnesium complex dications in a linear ion trap, which are subsequently fragmented by CID. The fragment spectra allow for distinction and relative quantification not only of Glc and Gal epimers (Figure 41C) but also of alpha versus beta anomeric linkages and provide further information on double bond positions in the fatty acyl chains.^636^ Complementary information about the lipid residues can be obtained by ozone-induced dissociation (OzID),^637^ which is based on the 1,3-dipolar cycloaddition of ozone to C=C bonds, followed by cleavage of the latter. OzID was applied to pinpoint C=C bonds in both the fatty acid and the sphingoid base of unsaturated GSLs based on their differential reactivity toward ozone.^638,639^ A similar approach is followed by the Paternò–Büchi reaction, which is a photochemical [2 + 2] cycloaddition between a C=C bond and a carbonyl group and leads to cleavage of C=C bonds upon activation by CID.^640^ The technique was applied to distinguish the C=C Δ15 and Δ17 regioisomers in the fatty acid of GalCer (d18:1/24:1) directly from tissue samples by MALDI-MS/MS.^641^ A comprehensive summary of advanced tandem mass spectrometry strategies for full structural characterization of lipids is provided in an excellent recent review.^642^

### 8.4 Ion Mobility–Mass Spectrometry of Glycolipids

The use of IMS in glycosphingolipidomics has been limited to very few studies in the past 15 years and remained largely restricted to the separation of gangliosides. MALDI-IM-MS was first used to separate all major brain gangliosides based on the number and position of sialic acids in the glycan headgroup in a drift tube setup.^643^ Even the structural isomers GD1a and GD1b, which differ by the position of a single sialic acid residue, were partially resolved in a mixture. Only recently was IMS coupled to desorption (D)ESI for MS imaging of gangliosides under ambient conditions using a commercial traveling wave instrument.^644^ DESI-IM-MS yielded complementary information to MALDI-IM-MS and offered clear trendlines enabling the separation of gangliosides from other lipid classes. The first workflow for the analysis of gangliosides by ESI-IM-MS(/MS) was implemented at the same time for the investigation of human brain gangliosides.^645^ The number of gangliosides detected in human hippocampi was increased up to 10-fold compared with MS analyses without prior IM separation,^646,647^ and the results confirmed a correlation between the degree of ganglioside sialylation and brain development. Atmospheric pressure DTIMS in combination with an optimized drift gas composition recently allowed for an enhanced separation of GD1a and GD1b isomers compared with previous DTIMS studies.^648^ In some cases, isomer resolution can be increased by the addition of coordinating cations. For instance, the separation of t18:0 GSL stereoisomers was shown to increase significantly by the addition of silver(I) ions in ESI-DTIMS.^239^ IM separation by traveling waves in SLIM^649^ lifted IMS resolution of isomeric lipids and glycolipids to new levels.^650,651^ The isomeric gangliosides GD1a and GD1b, which were only partially resolved by DTIMS, were baseline-separated in a mixture after a path length of only 1.25 m. Neutral GSLs could, however, not be separated to a sufficient degree. Sodiated Glc and Gal sphingosine were only partially separated after 60 m path length (Figure 41D), whereas no separation was achieved for the sodiated ceramide analogues Glc and GalCer. Recently, IMS was integrated into a workflow for automated GSL glycan identification.^652^ The HILIC–IM–MS workflow combines glucose units, CCS, and *m*/*z* information for automated structural assignment of glycans from procainamide-labeled GSLs and allowed differentiation between breast cancer subtypes based on the GSL profiles. The integration of IMS into workflows for GSL identification, for example, as potential tumor markers,^653^ is expected to increase in the future.

### 8.5 Gas-Phase Infrared Spectroscopy of Glycolipids

**Figure 42:**
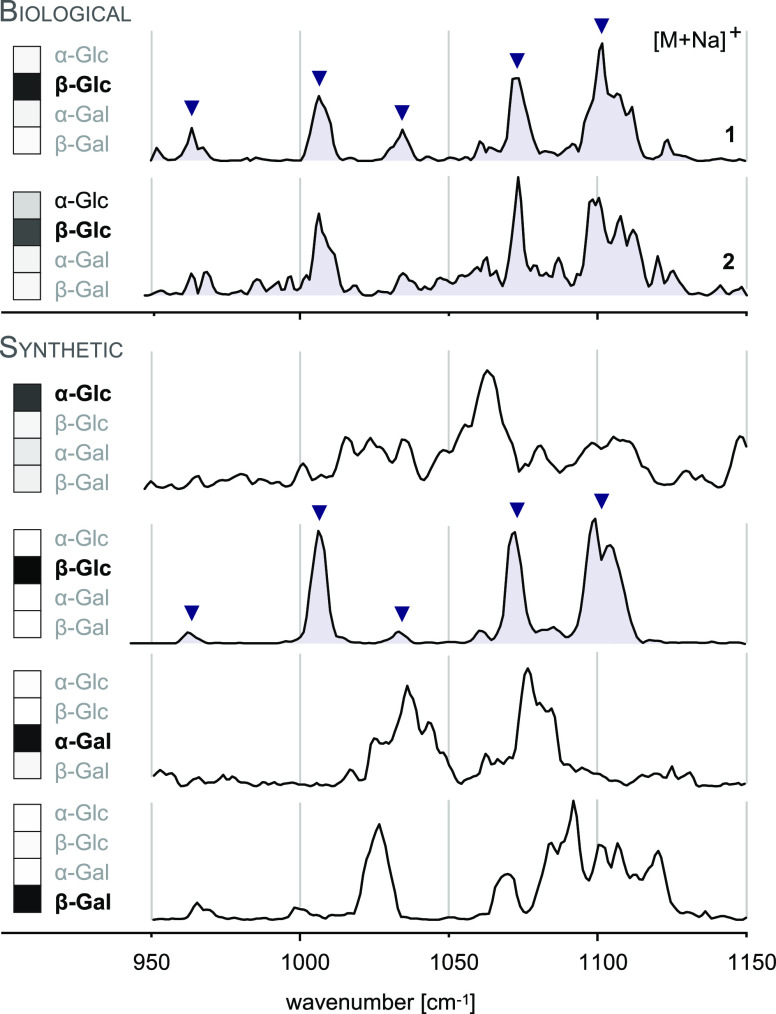
Spectral deconvolution of biological glycolipid mixtures into single isomers. IR spectra of biological mixtures from α-galactosidase (1) and α-glucosidase (2) knockout mice were deconvoluted to identify contributing isomers. IR spectra of the synthetic isomers α- and β-Glc and GalCer are required for isomer assignment. The main isomer in both biological samples is β-GlcCer, but α-glucosidase deficiency also leads to a measurable increase in α-GlcCer. Figure reprinted from ref (239). Copyright 2021 Kirschbaum et al. (Creative Commons Attribution 4.0 International License).

Recently, the first investigation of glycolipids by cryogenic gas-phase IR spectroscopy based on an MS detection scheme demonstrated that isomeric glycan headgroups can be distinguished by unique spectroscopic fingerprints in the mid-IR regime.^239^ The IR spectra not only are informative of Glc/Gal epimers (Figure 41E) but also reveal the anomeric configuration, which plays a crucial role in antibody–antigen interactions during natural killer T cell activation.^609^ The lipid residue, too, was found to influence the spectral signature and leads to a clear distinction between GSLs and glycoglycerolipids. Most importantly, biological mixtures of naturally coexisting GSL isomers were analyzed with the help of reference spectra obtained from synthetic standards (Figure 42). Spectral deconvolution by non-negative matrix factorization provided evidence of an increase in α-GlcCer in α-glucosidase-deficient mice. Regarding the power of high-resolution gas-phase IR spectroscopy to identify a range of different isomers and determine their relative abundance in isomeric mixtures, the development of commercial benchtop systems is highly desirable for future glycolipid analysis. First applications of UVPD spectroscopy are equally to be expected in the future.

## 9 Outlook

Even though no automated *de novo* glycan-sequencing method exists to date, significant progress has been made in the growing field of glycomics in the past decade. As outlined in this review, the analysis of mammalian glycans has profited enormously from recent developments of advanced MS-based techniques. Novel ion activation techniques exploiting ion–photon, ion–electron, and ion–ion interactions have given rise to previously inaccessible glycan fragments. Innovative derivatization strategies have not only improved detectability in MS experiments but also often led to more informative fragmentation patterns when combined with suitable dissociation methods. Hyphenation of ion mobility spectrometry to MS has enabled the postionization separation of isomeric species, and collision cross sections have been efficiently used to facilitate the identification and structural assignment of various glycans. Finally, gas-phase ion spectroscopy in the UV and IR range has provided information on the electronic and vibrational structure of mass-selected carbohydrate ions. When combined with advanced quantum chemical calculations, these analytical techniques reveal invaluable details on the underlying structure of the analytes, such as charge migration, conformational constraints, or intramolecular interactions in glycans in the gas phase.

The implementation of advanced MS-based techniques into multidimensional glycomics workflows holds great promise for the future. Democratization of MS-based technologies within the coming years is to be expected, which will render recently developed techniques accessible to the broader glycobiology community. The next decade will likely witness an increased implementation of high-energy ion activation techniques into LC-MS workflows. Advantages of novel fragmentation methods tailored specifically for the unique challenges posed by glycans or benefits stemming from increased resolution of ion mobility separations are evident. The commercialization of action spectroscopy would open a vast and largely unexplored field to nonexperts. Similarly to the situation of UVPD a couple of years ago, the key to this will lie in the commercial availability of affordable, easy-to-use light sources. Tunable benchtop laser systems are currently developing at a rapid pace, and significant progress can be expected in the near future. In addition, ion soft landing in combination with single-molecule surface analysis techniques has just demonstrated its immense potential for glycan analysis and awaits further exploration.^654,655^ Although several challenges are still waiting to be tackled, such as the integration of action spectroscopy into fast LC-MS methods, the importance of basic research to provide innovative solutions has become clearly apparent. In particular, the targeted development of techniques that consider the structure of carbohydrates and the unique challenges arising from them—instead of adopting methods from the MS-based proteomics toolbox—needs to be promoted.

## ABBREVIATIONS

MS: mass spectrometry
IMS: ion mobility spectrometry
DTIMS: drift tube ion mobility spectrometry
FAIMS: field asymmetric ion mobility spectrometry
TIMS: trapped ion mobility spectrometry
TWIMS: traveling wave ion mobility spectrometry
IM-MS: ion mobility–mass spectrometry
CID: collision-induced dissociation
HCD: higher-energy collisional dissociation
ExD: electron-based or electron-mediated dissociation
ECD: electron capture dissociation
EDD: electron detachment dissociation
EED: electronic excitation dissociation
EID: electron-induced dissociation
ETD: electron transfer dissociation
ETnoD: electron transfer with no dissociation
NETD: negative electron transfer dissociation
CTD: charge-transfer dissociation
RDD: radical-directed dissociation
EPD: electron photodetachment
a-EPD: activated electron photodetachment
IRMPD: infrared multiple photon dissociation
UVPD: ultraviolet photodissociation
DFT: density functional theory
MD: molecular dynamics
